# Defaunation is known to have pervasive, negative effects on tropical forests, but this is not the whole story

**DOI:** 10.1371/journal.pone.0290717

**Published:** 2023-08-31

**Authors:** Gust Boiten, Steffi Dekegel, Nikki Tagg, Jacob Willie

**Affiliations:** 1 Department of Biology, University of Antwerp, Antwerp, Belgium; 2 Centre for Research and Conservation, Royal Zoological Society of Antwerp, Antwerp, Belgium; 3 Earth and Life Institute, Université Catholique de Louvain, Louvain-la-Neuve, Belgium; 4 Born Free Foundation, Horsham, United Kingdom; Feroze Gandhi Degree College, INDIA

## Abstract

Ecosystem functioning and integrity are affected by the loss of large-bodied animals, and comprehending when and how ecosystems are affected is an important goal of defaunation ecology. Despite considerable investigation, our understanding is incomplete. Previous research is biased towards the study of seed dispersal in the Neotropics. This study examined whether and how defaunation affects stem density, species diversity, species composition, spatial distribution, and dispersal mode composition of young understorey plants in an Afrotropical setting. Rectangular plots along transects and wedge-shaped plots under focal trees of five mammal-dispersed species were used to compare three sites representing a defaunation gradient in the Dja faunal reserve in Cameroon. Results showed no change in stem density. Woody plant diversity was highest in the most defaunated site, and compositional differences were noted. Under focal trees, the overall abundance of both seedlings and juveniles was similar. The most defaunated site had the highest number of seedlings far from parent trees. More juvenile stems occurred near parent trees in the least defaunated site. This surprising trend might result from fruit dispersal by small, surviving animals and humans more easily collecting fruits, for food or medicinal purposes, in defaunated, more accessible sites. Negligible or no differences in the abundance of animal-dispersed species and other dispersal modes emerged. This study highlights the roles of extant taxa as surrogate providers of ecological services in defaunated Afrotropical forests. Hence, functional compensation is a serious possibility. Additionally, conceptual models of defaunation consequences that exclude the role of humans may not reflect real-world situations. Overall, these investigations suggest that tropical forests, especially those where ecological niches are less partitioned, may be more resilient to defaunation pressures than is often assumed. Effectively conserving extant, and perhaps less iconic, animal species provides hope for defaunated forests.

## 1. Introduction

Defaunation, defined as the decrease and annihilation of wild animals from natural systems, firstly affects large animal species [1–3]. The depletion of large-bodied herbivores translates to changes in plant-animal interactions and effects on the reproductive cycle of animal-dispersed plants [4, 5]. In a scenario of ecological distortion, all keystone mutualists become locally or ecologically extinct, and resulting drastic disruptions to ecological processes impede plant reproduction and the regeneration of plant communities [6, 7]. On the flip side, when some animal species are depleted in contexts where surviving animal species are functionally similar to extinct species, their ecological functions are continually available through compensatory services provided by the surviving species [8–10]. This scenario can be regarded as functional compensation.

Considerable effort has been devoted to investigating the effects of defaunation. Pioneer researchers hinted that forests deprived of fauna suffer negative ecological consequences [11, 12]. Subsequently, a growing body of empirical studies have highlighted the pervasive effects of defaunation. These include reduced dispersal of seeds and increased density of seeds and seedlings under parent plants [13, 14] and altered patterns of herbivory and seed predation [15, 16], with knock-on effects on plant community dynamics and structure, carbon sequestration, and plant biomass [7, 17, 18]. Although many papers have painted a bleak picture of the effects of defaunation, some studies paying special attention to the role of extant taxa have provided a more nuanced perspective. For example, seed scatter-hoarding by rodents has been reported to move seeds from source trees to places where stems of the dispersed species are rare and seeds and seedlings are more likely to survive and establish due to favourable conditions [19–21]. Even though seeds are cached to be eaten later, some eventually escape predation and grow [22]. Hence, results showing that food scatter-hoarding successfully disperses seeds and significantly contributes to plant regeneration [e.g. 9, 23, 24] are no surprise. It should be noted, however, that previous defaunation studies have mainly investigated seed dispersal [25], a function performed by a large array of vertebrates in tropical forests [22]. These studies largely focused on Neotropical systems [26]—where functional redundancy among frugivores is relatively low [27]—thus resulting in a literature plagued by topic-, geography-, and taxonomy-related biases -which limits the understanding of wild animal ecology and defaunation [3, 25, 26]. Indeed, in diverse Afrotropical systems, studies of the consequences of defaunation for plants are relatively rare. For example, research on the effects of defaunation on the understorey community structure, demographic structure, and functional trait composition is still relatively limited. In sum, what is currently documented regarding the consequences of defaunation for plants is not the whole story, and more empirical studies and robust data are therefore required.

A study of the effects of defaunation on understorey plants in Afrotropical forests can strengthen the body of empirical evidence from contexts other than the Neotropics and help correct for biases in defaunation studies, therefore providing a more comprehensive overview of defaunation consequences. In the Afrotropics, the relatively high functional overlap among vertebrates [27] and the continued occurrence of these species in varying abundances and differing guilds [7, 28] offer more possibilities for assessing how extant animal species shape ecological patterns and whether these patterns may point to functional compensation or ecological distortion. Thus, resulting insights can lend support to ideas regarding the ecological consequences of defaunation. The study can also underscore the role animals that are still in existence in forests may play in the maintenance of these ecosystems, thus guiding decisions as to whether special conservation efforts should be devoted to certain animal species persisting in defaunated areas.

In this study, the impact of defaunation on understorey plant communities is investigated, by comparing three sites along an Afrotropical defaunation gradient. Specifically, we address the following questions: i) How does defaunation influence understorey stem density, species diversity, and species composition? ii) To what extent does defaunation affect the abundance and spatial distribution of stems of different ages? iii) Is the composition of dispersal modes impacted by defaunation? As more than 80% of the woody species in Afrotropical forests depend on animals to disperse their seeds [29–31], a reduction in the abundance of mammalian dispersers is expected to result in a reduced stem density [32, 33]. According to the Janzen-Connell model, the lack of dispersal results in increased seed and seedling mortality and reduced recruitment [34–37]. Following this reasoning, we expect a reduced species diversity, as recruitment for most mammal-dispersed species becomes more difficult. Ultimately, this can result in changes in the species composition. Recruitment patterns under parent trees have been shown to be influenced by mammalian disperser abundance in several species. At sites with high levels of defaunation, reduced dispersal is expected to result in seedling aggregation close to the focal trees, while stronger dispersal at sites with a high mammal abundance leads to a higher number of seedlings and juveniles further away from the focal trees [13, 26, 38]. Based on the Janzen-Connell model, the survival of stems close to parent trees is expected to be low [34–37], resulting in an overall lower abundance of conspecific juveniles under focal trees at sites with reduced mammal abundances. As the defaunation of large and medium-sized mammals mainly affects recruitment of zoochorous species, we anticipate a reduced abundance of mammal-dispersed plants at sites impacted by higher levels of defaunation, making room for species with other dispersal modes [5, 39–42].

## 2. Material and methods

### 2.1 Study sites

**Fig 1:**
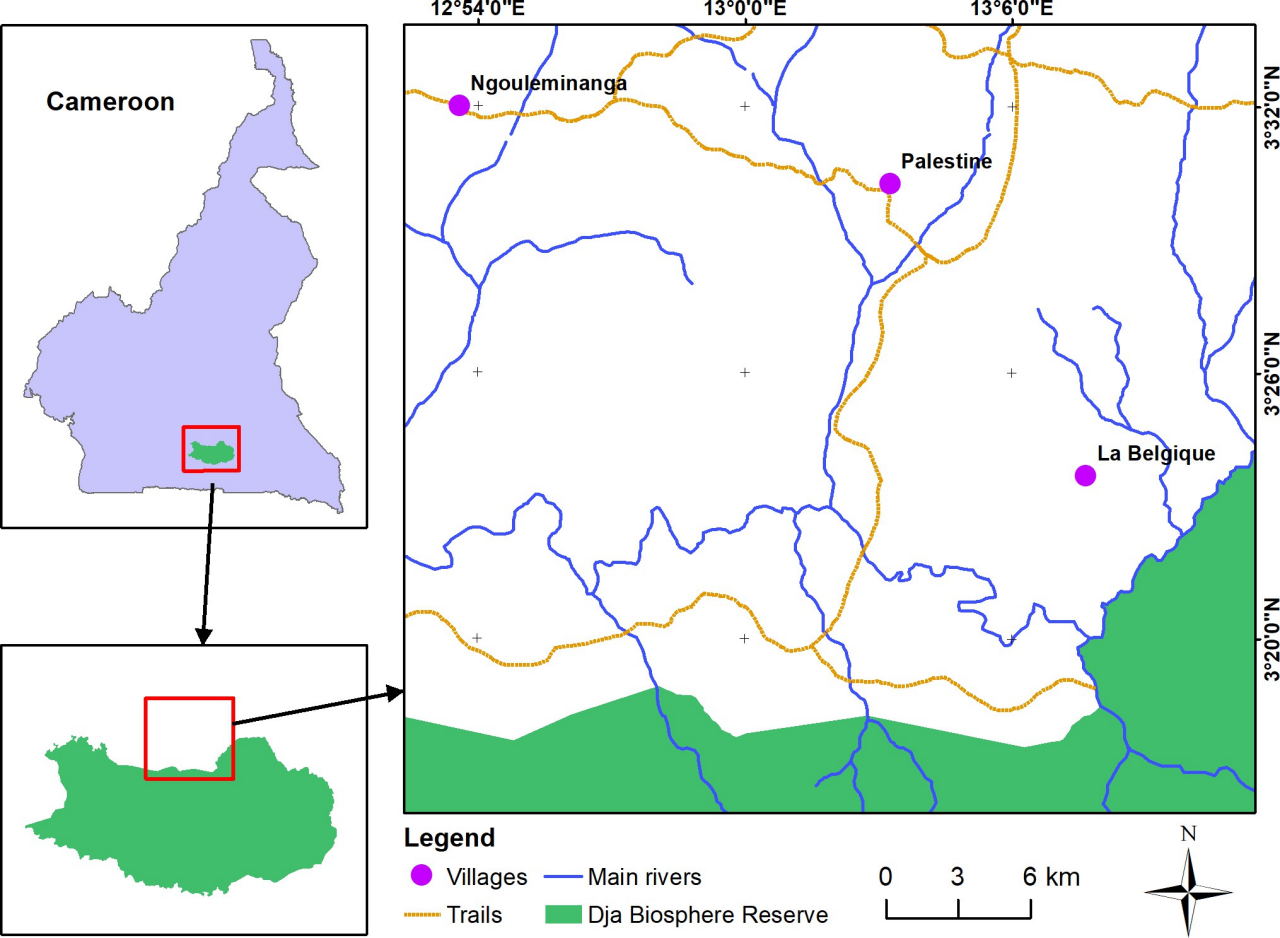
Map of the study area and location of the study sites (purple dots) in the northern buffer zone of the Dja Faunal Reserve, southeast Cameroon. Data source for the base files: https://cmr.forest-atlas.org/ [45].

Data collection took place at three sites in continuous forest in the northern buffer zone of the Dja Faunal Reserve in Cameroon: La Belgique (LB), Palestine (PA) and Ngouleminanga (NG) (Fig 1). The area has a humid equatorial climate with an average rainfall of approximately 1570mm [43] and experiences four seasons. The year starts (November to February) with an ongoing long dry season, followed by a long rainy season between March and June, a second short dry season from July to August, and a rainy season from September to November [44]. Temperatures remain relatively stable throughout the year. Measures between April 2009 and March 2010 showed temperatures between 19.8°C and 27.0°C on average [43].

Ngouleminanga and Palestine are the most accessible sites and therefore more subject to human activity, while La Belgique is more remote and less disturbed (Dekegel, Unpublished data).

### 2.2. Mammal abundance sampling designs

Mammal abundance data were collected by S. Dekegel over a 6-week period, in July and August 2017, in a larger area that includes the three study sites and three additional sites. Detailed information regarding wildlife in this landscape is available in S1 File.

To facilitate mammal abundance data collection 4 transects were opened in La Belgique, 7 transects in Palestine and 5 transects in Ngouleminanga (total length: LB = 24.00km; PA = 22.95km; NG = 25.95km). Three survey teams walked each transect once at a speed of approximately 1 km/h. The first team focused on collecting indirect observations. The second team did great ape nest surveys on the same transect, while the third team recorded all direct observations of wildlife along a different transect. Local guides were hired from neighbouring villages and had extensive hunting experience, knowledge in local animal signs, and experience in observing mammals in the forest.

#### 2.2.1. Direct wildlife observations

Any direct observation of mammals was recorded along the transects following line-transect distance sampling techniques. For each individual or group encountered, the direct distance from the observer to an individual or group centre was estimated visually and the sighting angle from the transect line was determined using an orientation compass. Researchers occasionally left the transect to confirm group size, but all individuals or groups were initially detected from the transect line.

#### 2.2.2. Indirect wildlife observations

All indirect signs of animal activity were recorded using the fixed-width strip-transect technique. Evidence of animal presence such as feeding remains, vocalization, sleeping site, dung, footprints, and tracks were recorded within a distance of 1 m on each side of the transect line.

#### 2.2.3. Great ape nest surveys

Gorilla and chimpanzee nests were surveyed using the standing crop nest count methodology. This method is based on line-transect distance sampling and is a widely used approach to estimate great ape population sizes [46, 47].

When a nest was detected, an area of 30m radius was searched for additional nests. All nests of similar age category located within 20 m or 30 m, for gorillas and chimpanzees respectively, were considered as belonging to the same group [48]. Additional nest characteristics and evidence such as hairs, odour, knuckle prints, and faeces were used to reliably confirm nest builder identity.

### 2.3. Vegetation sampling designs

At each site five transects were opened in 2019 to facilitate vegetation data collection. The transects at each site were parallel to each other, with approximately 600 metres in-between. In La Belgique, the transects reached the desired length of 5km and totalled 25km. However, large rivers limited the accessibility in Palestine and Ngouleminanga, resulting in shorter transects (total length Palestine: 14km; Ngouleminanga: 18km) and smaller sampling sizes.

#### 2.3.1. Conspecific density and demographics near focal trees

Five focal tree species were selected based on their dispersal mechanisms: *Klainedoxa gabonensis*, *Tetrapleura tetraptera*, *Chrysophyllum lacourtianum*, *Duboscia macrocarpa*, and *Antrocaryon klaineanum*. All species are mainly dispersed by mammals and depend on endozoochory by large frugivores [e.g. 49–54] (Table A in S1 Table).

Based on the methodology of Cordeiro and Howe (2003), wedge-shaped plots were used to count conspecific stems under focal trees [13]. With increasing distance from the base of the focal tree, the dispersal area under the canopy strongly increases, resulting in lower seed densities further from the tree. The wedge shape of these plots takes into account this change in dispersal area and seed/seedling density away from a focal tree, preventing census bias [38].

**Fig 2:**
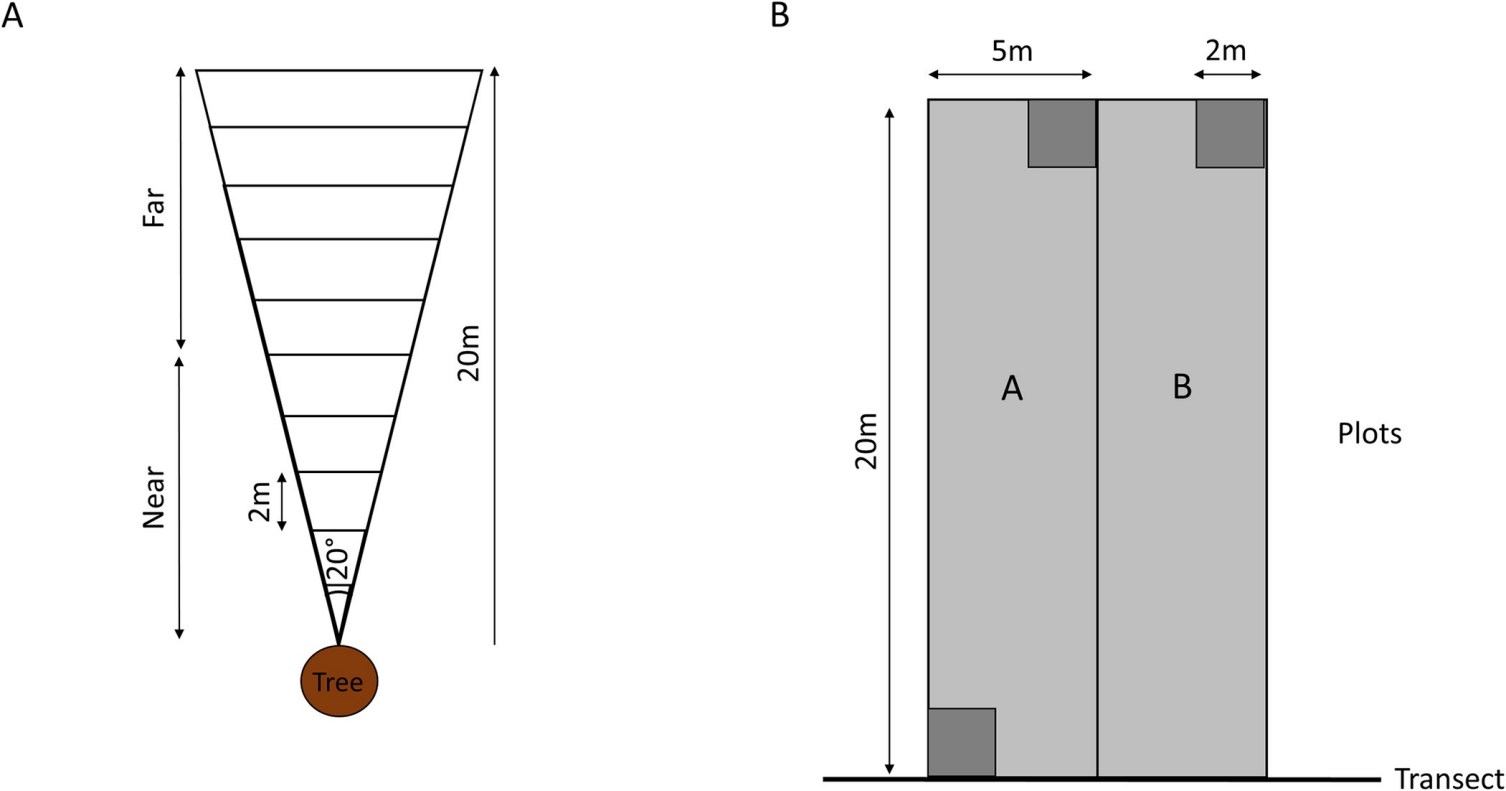
Vegetation sampling designs. Schematic representation of (A) wedge-shaped plots under focal trees and (B) rectangular plots along the transects.

Wedges were placed under reproductive individuals of the five study species at each site. The search for these focal trees was not restricted to the trees in direct proximity of the transects. Under every focal tree a single wedge was placed. Wedges were placed in random directions under focal trees, but placement on trails was avoided. To increase the probability that the recorded stems originated from the focal trees, wedges were only placed under focal trees without any mature conspecifics in their surroundings (>50m radius). The wedge-shaped plots, with a length of 20m and an angle of 20° at the base of the tree, were constructed using ropes. At every two-metre interval they were divided into subplots, resulting in ten subplots located at increasing distances from the base of the tree. However, during analyses, data from the five subplots closest to the base of the tree (0-10m) were pooled together to form a main subplot regarded as ‘Near’, while the remaining five subplots (10-20m) were pooled together and regarded as ‘Far’ (Fig 2A).

In the wedges all conspecific stems up to 1m in height were identified and counted. Counted stems were assigned to one of two classes based on their height. Class 1 consisted of stems smaller than 15cm and Class 2 included stems with a height from 15 up to 100cm. Class 1 stems can be considered as seedlings that germinated less than a year before identification, while Class 2 stems were juveniles older than 1 year, but the exact age was unknown.

Originally it was planned to collect data from eight focal trees of each study species at the three sites. Difficulties with finding the trees and limited time for data collection prevented reaching the desired number of trees at all sites, leading to smaller sample sizes in Palestine and Ngouleminanga (Table 1).

**Table 1:** Number of focal trees of the five selected species sampled at each study site.

Species	Family	La Belgique	Palestine	Ngouleminanga
*Klainedoxa gabonensis*	Irvingiaceae	8	5	5
*Tetrapleura tetraptera*	Mimosaceae	8	7	8
*Chrysophyllum lacourtianum*	Sapotaceae	8	1	3
*Duboscia macrocarpa*	Tiliaceae	8	1	8
*Antrocaryon klaineanum*	Anacardiaceae	8	4	8
Total		40	18	32

#### 2.3.2. Understorey and overstorey composition

The understorey and overstorey vegetation were sampled using rectangular plots that were placed along the five transects at each study site (Fig 2B). At each kilometre mark (starting at 0km), a plot of 10x20m was set up, always on the same side throughout the whole transect. These plots were divided into two subplots of 5x20m, Plot A and Plot B, referred to as “large plots”. In the bottom left and the top right corner of Plot A, and in the top right corner of Plot B, smaller 2x2m plots were set up. These plots are referred to as “small plots”. In large plots (which also encompasses the nested small plots) all woody stems taller than 1m were identified, their circumference was measured, and their height was estimated. In the small plots all woody stems up to one metre in height and all herbaceous stems were identified. Species identification at all sites was done by the same local botanical guide, who provided the plant names in the local language, Badjué. These were later translated into their scientific names based on an existing list of known species in the area (created by the “Projet Grands Singes [PGS]”). This list was created over many years by sending specimens for each newly recorded species to the national herbarium of Cameroon for confirmation of identification. Plants that were not yet present in this list were indicated as unknown species followed by the morphospecies of the plant and a number (for example “Unidentified_liana_2”). In the small plots, a total of 185 species (23 at genus level; 10 unidentified), and in the large plots, a total of 216 species (22 at genus level; 10 unidentified) were recorded. Data were collected at the three sites in July–September 2019.

### 2.4. Statistical analyses

Statistical comparisons were performed using R version 4.0.2 [55]. All vegetation and mammal abundance data were non-normally distributed. Comparisons between the three study sites were therefore done using the non-parametric Kruskal-Wallis test with the kruskal.test function. When this resulted in significant differences (α < 0.05), it was followed up by a pairwise comparison with the pairwise Wilcoxon rank-sum test using the pairwise.wilcox.test function in R. In case strong outliers were present, the median test from the ‘coin’ package [56] was used instead and followed up by a pairwise median test using the pairwiseMedianTest function from the ‘rcompanion’ package [57]. In case the shape assumption of these tests was not met, the Kruskal-Wallis test was used to compare the mean ranks instead of the median. The Holm correction was used to correct for multiple testing [58].

Data from the large plots were used as characterisation of the study sites and served as a control, to check whether the effects found in the understorey were not the result of differences in the vegetation structure of the adult assemblages. The results for these analyses are presented in the S2 File.

#### 2.4.1. Mammal abundance

To compare the mammal abundance between sites, transects within each site were divided into 600 m segments and every other segment was included in the analysis to obtain independent observation units [59].

The species encounter rate (number of signs per km) was used as index of abundance and was calculated for each 600 m segment. Analyses were conducted on species level and on taxonomic guild level. Species were pooled into seven taxonomical guilds: 1) even-toed ungulates, 2) rodents, 3) carnivores, 4) pangolins, 5) elephants, 6) great apes, and 7) Old World monkeys. Indirect wildlife data were used for the first five guilds whereas nest counts where used to estimate great ape abundance. Individual great ape nests were used as a measurement instead of nest sites to avoid possible bias arising from grouping individual nests into nest sites. Direct observations were used to generate abundance estimates for arboreal monkeys based on the total number of individuals observed per segment [59–61]. As accurate density estimations require a minimum of 60–80 observations for each species, species densities (individuals/km) could not be calculated since the number of observations for each species was not high enough.

#### 2.4.2. Understorey and overstorey plant community structure

*2*.*4*.*2*.*1*. *Stem density*. The stem densities of herbaceous and woody stems were calculated for each plot and compared between sites.

Importance values were calculated for all species in the large plots. This value was calculated by summing the relative frequency, relative density, and relative dominance for each species at each study site [62, 63].

*2*.*4*.*2*.*2*. *Species composition*. To compare the understorey and overstorey species composition between the three study sites the multi-response permutation procedure (MRPP), with the Sorensen distance measure, was used. This was done by running the mrpp() function from the ‘vegan’ package in R. The MRPP was used to analyse the species composition of herbaceous and small woody stems at the three sites. The MRPP provides a chance-corrected within group (site) agreement value (A) and a p-value [64]. To increase the robustness of this analysis, species that were present in less than 5% of the plots were removed from the MRPP dataset, leaving 18 out of 39 herbaceous species and 35 out of 146 woody species. To compare the three sites, separate analyses were done for each pairwise combination of the sites (Pairwise comparisons: LB-NG, LB-PA and NG-PA). To adjust for multiple comparisons the Holm correction was used on the P-values [58].

*2*.*4*.*2*.*3*. *Species diversity*. The program EstimateS 9.1.0 [65] was used to calculate the Shannon diversity index and extrapolated rarefaction curves for the data from both small and large plots. Rarefaction and extrapolation in EstimateS use the Bernouilli product model [66], a method that takes differences in sampling sizes into account.

#### 2.4.3. Conspecific density and demographics near focal trees

Using data from the wedges, general densities of Class 1 and 2 stems under focal trees were calculated and compared between sites. A second analysis was done, where the recorded stems were separated into two groups, stems <10m (near) and 10–20m (far) away from the base of the focal trees. The number of Class 1 and Class 2 stems in each section were compared between sites. These analyses were done for all focal tree species pooled together to identify general patterns and separately to examine species-specific differences.

When the sampling size in Palestine was too small (n = 1) a Wilcoxon-Mann-Whitney U test was performed, using the wilcoxon.test function, to only compare stem densities between La Belgique and Ngouleminanga. Tree productivity was assumed to be similar at the three study sites because the sampled trees did not differ significantly in size between sites (Table B in S1 Table).

#### 2.4.4. Functional trait composition

The dispersal mode composition was compared between the three study sites. The dispersal modes of 94 out of 185 species in the small plots and 130 out of 216 species in the large plots were identified from literature (Table C in S1 Table). Six modes of dispersal were differentiated: bird, mammal, wind, water, explosion, and drop dispersal. A single species could be assigned multiple dispersal modes. At the three sites similar proportions of species with each dispersal mode were identified (Table D in S1 Table), making a good comparison possible. Table E in S1 Table shows the total number of species with identified dispersal modes per site and plot type. The abundance of stems (per plot) with each dispersal mode was compared between the sites. This analysis was done separately for woody stems < 1m and herbaceous stems in the small plots, and with the complete dataset (all stems larger > 1m) and only the largest stems (trees ≥10cm and lianas ≥5cm in diameter) in the large plots.

### 2.5. Ethics statements

Research permits for the study areas were provided by the Cameroon Ministry for Scientific Research and Innovation. This study is based on observational data and no endangered or protected plant species were collected. Additional information regarding the ethical, cultural, and scientific considerations specific to inclusivity in global research is included in the Supporting Information (S1 Checklist).

## 3. Results

### 3.1. Mammal abundance

According to the mammal abundance data (Tables 1 and 2 in S1 File) the three study sites formed a defaunation gradient. The overall mammal abundance (abundance/km: LB = 107.00 SE6.24; PA = 57.72 SE5.77; NG = 23.65 SE1.85) based on indirect observations differed significantly between all three sites (LB-PA: P-value <0.01; LB-NG: P-value <0.001; PA-NG: P-value <0.001). La Belgique had the most intact mammal community, with large bodied species including forest elephants (*Loxodonta cyclotis*), western lowland gorillas (*Gorilla gorilla gorilla*), and central chimpanzees (*Pan troglodytes troglodytes*) still present. Furthermore, this site had a relatively high abundance of large and small ungulates and old-world monkeys (Cercopithecidae). Palestine had an intermediate mammal abundance along the gradient, while Ngouleminanga had the most depleted mammal community. There were no indications of the presence of forest elephant, western lowland gorilla, and central chimpanzee populations found in both Palestine and Ngouleminanga. While the abundance of large ungulates was similar in La Belgique and Palestine, this group had a strongly reduced abundance in Ngouleminanga. Small ungulates had a gradually decreased abundance along the gradient, while the abundance of monkeys did not differ significantly between the sites. Finally, rodents had a higher abundance in Palestine compared to the other sites. Overall, rodents were equally abundant in La Belgique and Ngouleminanga, although the abundance of the forest giant pouched rat (*Cricetomys emini*) was higher in Ngouleminanga. The contribution of small ungulates and rodents to the total mammalian species assemblage was increased in Palestine and Ngouleminanga.

### 3.2. Understorey plant community structure

#### 3.2.1. Stem density

**Fig 3:**
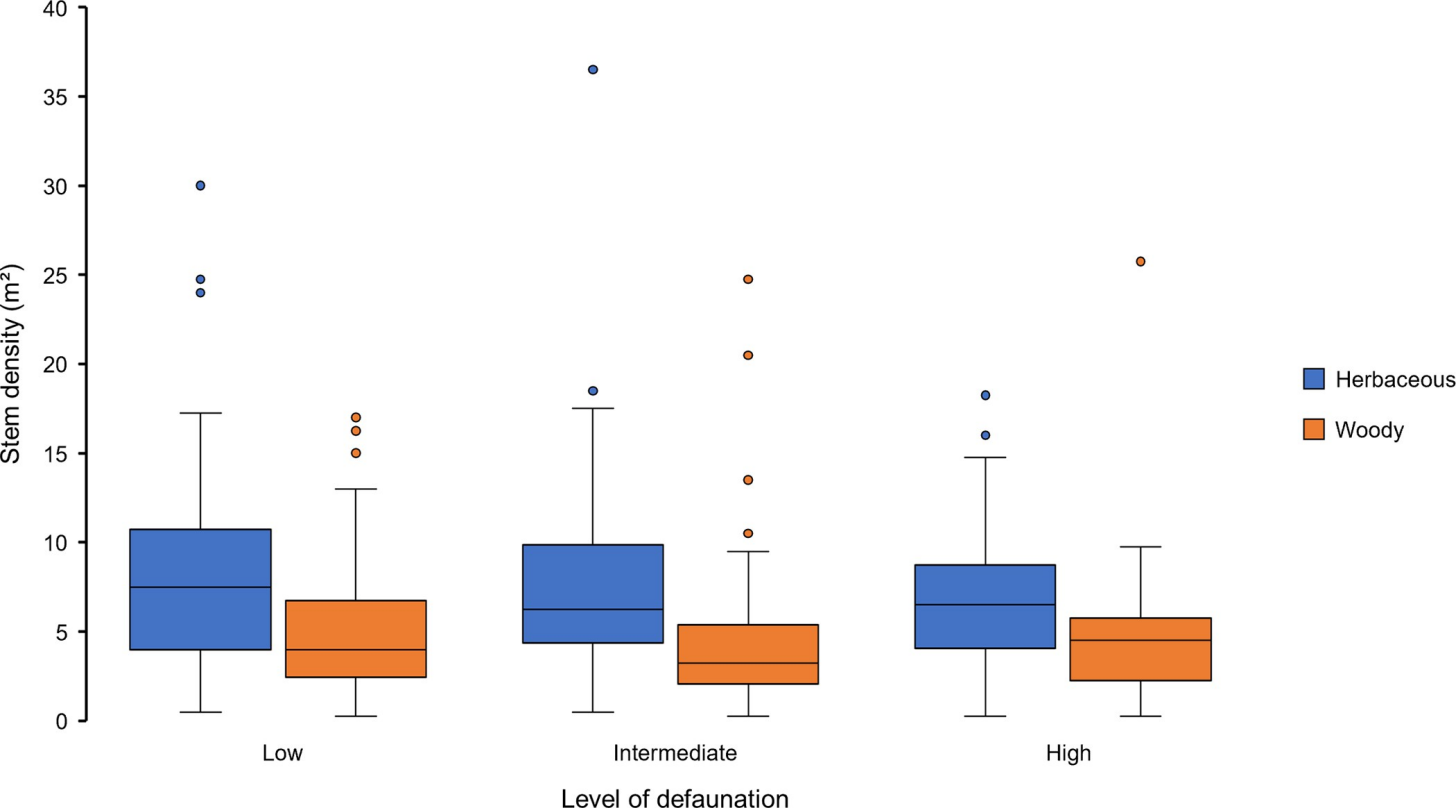
Boxplots presenting the density data for woody stems ≤ 1m (orange) and herbaceous stems (blue) at the three study sites. The three study sites: La Belgique (low), Palestine (intermediate), and Ngouleminanga (high).

The stem density of the understorey vegetation was compared between the study sites and did not show any clear differences along the defaunation gradient. In the small plots, both small woody stems and herbaceous stems occurred at similar densities at all study sites (Table F in S1 Table; Fig 3).

#### 3.2.2. Species composition

To assess whether the species composition of the understorey vegetation varied between the study sites, the MRPP was performed (Table 2). This analysis indicated that the species compositions of small woody stems (≤ 1m) were more homogenous within sites than would be expected by chance (P-value <0.05). A pairwise analysis indicated that the species composition of small woody stems differed between La Belgique (the least defaunated site) and Ngouleminanga (the most defaunated site), but differences with Palestine (the intermediate site) were not significant. Furthermore, the species composition of herbaceous vegetation was found to differ significantly between all sites. However, all within-site agreement values for both woody and herbaceous stems approached zero, indicating that homogeneity within sites was low and was similar to the expected heterogeneity. So, even though the MRPP indicated statistically significant differences in understorey species composition between the study sites, the low level of within site homogeneity suggests that the species compositions had much overlap among the study sites.

**Table 2:** Results of the multiple response permutation procedure (MRPP) analyses to compare the understorey species composition between the study sites.

**Small plots: Woody stems ≤1m**		
**Sites**	**A**	**P-value**
All sites	0.0068	0.004 **
La Belgique—Palestine	0.0037	0.070
La Belgique—Ngouleminanga	0.0081	0.012 *
Palestine—Ngouleminanga	0.0024	0.11
**Small plots: Herbaceous stems**		
**Sites**	**A**	**P-value**
All sites	0.0083	0.001 **
La Belgique—Palestine	0.0092	0.003 **
La Belgique—Ngouleminanga	0.0046	0.022 *
Palestine—Ngouleminanga	0.0049	0.026 *

The results for woody (≤1m) and herbaceous stems are presented separately. The chance-corrected within group agreement (A) describes the homogeneity within sites; the maximum value of 1 indicates that all stems are identical within sites [64]. The three study sites: La Belgique (least defaunated), Palestine (intermediate), and Ngouleminanga (most defaunated). Significant differences between sites are indicated

* = P<0.05

** = P < 0.01

*** = P < 0.001.

#### 3.2.3. Species diversity

The EstimateS analysis revealed that the species diversity of the understorey vegetation differed between the study sites. Understorey diversity for woody stems showed the strongest difference between Ngouleminanga and the other sites, with the highest Shannon (H) diversity in Ngouleminanga (H = 3.43). La Belgique (H = 2.81) and Palestine (H = 2.92) showed a more similar level of diversity. Herbaceous stem diversity was highest in Ngouleminanga (H = 2.9) site, slightly lower in La Belgique (H = 2.84), and lowest in Palestine (H = 2.5).

The extrapolated rarefaction curves, for woody stems (Fig A in S1 Fig), indicated differences in species richness between the study sites. Ngouleminanga and La Belgique had comparable levels of species richness with much overlap in their curves. Palestine had a lower species richness compared to La Belgique, with no more overlap at the end of the curve. Compared to Ngouleminanga, the species richness in Palestine was slightly lower but the confidence intervals started to overlap again at the end of the extrapolated curves. The curves for herbaceous stems greatly overlapped for all sites (Fig B in S1 Fig).

### 3.3. Conspecific density and demographics near focal trees

Recruitment patterns under the five focal tree species were compared between the three study sites. The general densities of stems under the focal trees were similar at all sites, for most focal tree species. A general analysis of all five species pooled together showed no significant differences between the study sites for both seedlings (Class 1) and juveniles (Class 2). However, testing the species separately revealed differences in stem densities for two focal tree species. Post-hoc pairwise comparisons indicated that Ngouleminanga had a higher density of *K*. *gabonensis* Class 1 stems, compared to La Belgique (P-value = 0.012), while Class 2 stems were at a higher density in La Belgique compared to Ngouleminanga (P-value = 0.0093). In Ngouleminanga, no Class 2 stems were recorded under *K*. *gabonensis* focal trees. Further, the density of Class 1 stems under *D*. *macrocarpa* was higher in La Belgique compared to Ngouleminanga. The stem density under *C*. *lacourtianum* focal trees showed a similar trend as *K*. *gabonensis*, with a higher density of Class 1 stems in Ngouleminanga compared to La Belgique and a higher density of Class 2 stems in La Belgique compared to Ngouleminga. However, this difference was statistically non-significant. The other focal tree species had similar stem densities at all sites (Table G in S1 Table).

The distribution of seedlings (Class 1) and juvenile stems (Class 2) under focal trees was assessed by comparing stem densities near (<10m) to and far (10–20m) from the focal trees. Both general and species-specific differences were identified between the study sites. The results from this analysis are presented in Table 3, and the percentage distribution of stems can be found in Table H in S1 Table. With all species pooled together, the abundance of Class 1 stems far from focal trees and Class 2 stems near focal trees differed between sites. Pairwise comparison revealed that Ngouleminanga had a higher number of Class 1 stems far from the focal trees compared to La Belgique (P-value = 0.0072). Class 2 stems, on the other hand, were present in higher numbers near focal trees in La Belgique compared to the other sites (P-value = 0.016). However, when analysed separately, species-specific differences in recruitment patterns emerged. Detailed information regarding species-specific differences is available in S3 File.

**Table 3:** Comparison of the number of stems <10m (Near) and 10–20m (Far) from the focal trees between sites.

Class 1		Median abundance			Test (df = 2)	
Species	Distance	La Belgique	Palestine	Ngouleminanga	χ^2^	P-value
All together	Near	4	3.5	2.5	2.51	0.29
	Far	3	3	6.5	9.36	0.0093 **
*T*. *tetraptera*	Near	4.5	3	1	13.19	0.0014 **
	Far	5	3	1.5	1.20	0.55
*C*. *lacourtianum*	Near	2	11	2	(U) 13.50	0.83
	Far	1	8	7	(U) 0.50	0.022 *
*K*. *gabonensis*	Near	1	3	3	3.12	0.21
	Far	1	1	7	6.18	0.046 *
*D*. *macrocarpa*	Near	20	12	5	(U) 59.00	0.0051 **
	Far	30	11	8.5	(U) 40.50	0.40
*A*. *klaineanum*	Near	4.5	14	2	2.43	0.30
	Far	4	26	6	0.53	0.77
**Class 2**		**Median abundance**			**Test (df = 2)**	
**Species**	**Distance**	**La Belgique**	**Palestine**	**Ngouleminanga**	**χ^2^**	**P-value**
All together	Near	1	1	0	8.71	0.013 *
	Far	1	1	0	3.09	0.21
*T*. *tetraptera*	Near	0	1	0	1.90	0.39
	Far	0.5	1	2	5.72	0.057
*C*. *lacourtianum*	Near	2.5	0	1	(U) 21.00	0.068
	Far	1	1	0	(U) 20.00	0.10
*K*. *gabonensis*	Near	1.5	1	0	8.34	0.015 *
	Far	1	0	0	6.13	0.047 *
*D*. *macrocarpa*	Near	0	0	0	(U) 36.00	0.38
	Far	0	0	0	NA	NA
*A*. *klaineanum*	Near	1	1	1	0.50	0.77
	Far	1.5	1	0.5	0.41	0.81

The results for Class 1 stems (juveniles <15cm, top) and Class 2 stems (juveniles 15–100cm, bottom) are presented separately. The median abundance, significance, and test statistic (χ^2^) are shown. As the sample sizes for *C*. *lacourtianum* and *D*. *macrocarpa* were too small in Palestine (n = 1), the Wilcoxon-Mann-Whitney U test was used to compare the number of stems between La Belgique and Ngouleminanga. For these species the U statistic is presented instead of the χ^2^. The three study sites: La Belgique (least defaunated), Palestine (intermediate), and Ngouleminanga (most defaunated). Significant differences between sites are indicated

* = P<0.05

** = P < 0.01

*** = P < 0.001.

### 3.4. Understorey functional trait composition

#### 3.4.1. Dispersal mode composition

The dispersal mode composition of the understorey vegetation was compared between the three study sites. Differences in the abundance of dispersal modes for small woody stems (≤1m) were only found regarding wind dispersal (Table 4). Although the median test showed a significant difference, the post-hoc pairwise test was not significant (LB-NG P-value = 0.082; PA-NG P-value = 0.082) (Fig C in S1 Fig).

**Table 4:** Comparison of dispersal modes for small woody plants (≤1m) between the sites.

	La Belgique		Palestine		Ngouleminanga		Test (df = 2)	
Dispersal mode	Median abund.	CI	Median abund.	CI	Median abund.	CI	χ^2^	P-value
Bird	3	2; 4	3	3; 4	2	2; 4	0.77	0.68
Mammal	2	1; 3	1.5	1; 2	2	1; 3	0.69	0.71
Wind	0	0; 0	0	0; 0.5	1	0; 1	6.28	0.043 *
Water	0	0; 0	0	0; 0	0	0; 0	0.77	0.68
Explosion	0	0; 0	0	0; 0	0	0; 0	1.56	0.46
Drop	1	1; 2	1	0.5; 2	1	0; 2	1.08	0.58

The median abundance, 95% percentile confidence interval (CI), significance, and test statistic (χ^2^) are shown. The three study sites: La Belgique (least defaunated), Palestine (intermediate), and Ngouleminanga (most defaunated). Significant differences between sites are indicated

* = P<0.05

** = P < 0.01

*** = P < 0.001.

There was a significant difference in the abundance of mammal- and bird-dispersed herbaceous stems between the study sites (Table 5; Fig D in S1 Fig). Post-hoc pairwise comparisons revealed that the median abundance of mammal-dispersed herbs was higher at La Belgique (least defaunated) compared to Palestine (intermediate) (P-value = 0.014). However, the data showed only a small difference and a lot of variation and overlap, therefore this effect cannot be considered practically significant. Bird-dispersed stems were slightly more abundant in Palestine compared to the other sites, with mean ranks of 103.24 in La Belgique, 120.55 in Palestine, and 98.90 in Ngouleminanga (most defaunated). However, post-hoc pairwise comparisons were not significant (LB-PA P-value = 0.092; PA-NG P-value = 0.078). Furthermore, this difference was influenced by a single outlier.

**Table 5:** Comparison of dispersal mode abundances of herbaceous stems between the sites.

	La Belgique		Palestine		Ngouleminanga		Test (df = 2)	
Dispersal mode	Median abund.	CI	Median abund.	CI	Median abund.	CI	χ^2^	P-value
Bird	0	0; 0	0	0; 3	0	0; 0	6.13	0.047 *
Mammal	6	5; 8	4	3; 5	5	3; 7	8.11	0.017 *
Wind	0	0; 0	0	0; 0	0	0; 0	4.08	0.13
Drop	1	0; 2	2	0; 3	2	0; 3	1.14	0.57

The median abundance, 95% percentile confidence interval (CI), significance, and test statistic (χ^2^) are shown. For abundances of bird-dispersed stems, the shape assumption of the median test was not met; therefore, the mean ranks were compared using the Kruskal-Wallis test. The three study sites: La Belgique (least defaunated), Palestine (intermediate), and Ngouleminanga (most defaunated). Significant differences between sites are indicated

* = P<0.05

** = P < 0.01

*** = P < 0.001.

## 4. Discussion

The density of small understorey stems remained unchanged with the reduction in mammal abundance. Species diversity of woody stems was highest at the site with the strongest level of defaunation. The intermediate site had the lowest species richness and diversity for herbaceous species. The difference in woody species composition increased gradually along the defaunation gradient. Herbaceous species composition varied between all sites but did not follow the gradient, as the intermediate site differed most from the other sites. The lack of change in stem density may mean that mortality and patterns of seedling clustering are comparable across sites, possibly due to similar extents of processes such as seed dispersal, seed predation, folivory, and stem damage. This finding does not support the prediction that decreased abundance of mammalian dispersers would lead to a low stem density, at least not in the timeframe considered in this study. It is however possible that defaunation occurred relatively recently and that stronger ecological changes have yet to occur. Conflicting results were obtained in previous studies, some of which reported an increase in stem density as a result of defaunation [67, 68], while others showed a decrease [32, 33]. The change in stem density reported in previous studies, unlike this study, may result from an alteration of the animal-mediated processes underpinning the life cycle of plants. The relatively high woody plant diversity in the most defaunated site probably accounts for its different woody species composition, but the composition of this understorey woody community was very similar to the adult assemblage. This means that trends in the diversity and composition of younger plants may be explained by the structure of the mature vegetation rather than, contrary to expectation, a reduction in the abundance of mammalian dispersers. Chaves et al. (2015) reported similar results, with a strong relationship between the adult and seedling assemblages and no clear overall impact from defaunation pressures [69]. However, these findings contradict the studies that explained a lower species richness, diversity, and evenness by a reduction of large and medium-sized mammals [32, 70, 71]. These studies did not take the adult assemblages into account and might have provided imprecise evaluations of the impact of defaunation. Drivers such as water availability, temperature, light, and soil fertility may also affect plant community structure and should therefore be measured in future defaunation studies. However, the present study may still be regarded as an assessment of defaunation effects because all study sites fall under the same climate given their geographic locations, habitat composition is similar across sites in the region [59], and soil chemical fertility does not vary among habitat patches [72]. Hence, it can be inferred that, within the considered timeframe, defaunation events do not necessarily translate to changes in plant density, species diversity, or species composition, as also evidenced by extended botanical data in elephant-depleted sites [73, 74]. However, it is possible that the defaunation gradient was not strong enough. Even though La Belgique was the least defaunated site, it is not untouched and overall mammal abundances were low.

The overall abundance of both seedlings and juveniles under parent trees did not differ along the defaunation gradient. The highest number of seedlings far from parent trees was noted in the most defaunated site. Regarding juveniles, the highest number of stems near parent trees was noted in the least defaunated site. The consistency of seedling and juvenile abundance signifies that rates of survival and recruitment are similar in all sites, probably because factors such as dispersal, herbivory, stem damage, attacks from host-specific enemies, and resource competition affect all sites in a similar way. This result does not confirm the proposition that the smallest number of conspecific juveniles under focal trees would be found in the most defaunated site. Further, these results do not corroborate the findings of Cordeiro & Howe (2003) who documented a high abundance of seedlings under parent trees in sites with reduced disperser abundance [13]. In Cordeiro & Howe’s study, reduced disperser abundance had severe effects [75], but this may not be the case in the present study given that seedlings were equally present in all sites regardless of the defaunation level. The highest seedling abundance far from parent trees in the most defaunated site hints that seed dispersal continues unabated despite the decrease in disperser abundance. For example, *K*. *gabonensis* is dispersed by elephants, gorillas, chimpanzees, and other frugivorous mammals, like monkeys and duikers, who feed on the mesocarp of the fruits [52–54, 76–78]. Unlike elephants, small frugivores do not swallow the fruit whole, but they often carry it in their mouth for some distance before eating the mesocarp [76]. Despite the scarcity of large animals in the most defaunated sites, species of monkeys and duikers persist (Table 1 in S1 File). Therefore, this seedling distribution may be due to an increased contribution to seed dispersal by the remaining dispersers. Additionally, given that fruits of study species are also used by humans for consumption and/or medical purposes [50, 51, 76, 79–83], it is likely that locals collect fruit from under the parent trees especially in the more accessible, most defaunated sites, thus also contributing to observed patterns. Furthermore, higher levels of seed predation at more defaunated sites might have impacted the observed patterns, although mainly the intermediate site had a higher abundance of some seed predators (rodents) (Tables 1 and 2 in S1 File). These factors may also explain why, compared to other sites, more juvenile stems occurred near parent trees in the least defaunated site. These outcomes are not in line with projections of higher occurrences of seedlings near parent trees at highly defaunated sites and higher seedling and juvenile abundance far from parent trees in less defaunated sites. A study conducted in Tanzania reported higher seedling and juvenile abundances near parent trees when disperser abundances were reduced. Additionally, the percentage of stems far from focal trees was four times higher when disperser abundance was high [13]. Again, these patterns result from a reduced disperser abundance [75], and this constraint did not appear to restrict dispersal in the present study. This study has limitations, and to overcome them, future studies should use genetic methods to confirm parental links between young stems and adult trees under which they grow [38, 84] and assess dispersal of stems over longer distances. They should also account for abiotic means of dispersal as confounding variables. Furthermore, future studies should measure defaunation more robustly, and try to include an untouched site as a control. Nevertheless, in agreement with Jansen et al. (2012) and, Hawthorne and Parren (2000), these investigations indicate that in defaunated areas, some failures in ecological functions, which would have otherwise compromised plant reproduction, may be counterbalanced to some extend by beneficial activities by small, extant animals [9, 73]. Even though this is likely timescale and species dependent, this study upholds the notion that systems where ecological niches are less partitioned are more resilient to defaunation pressures [27].

Among sites, differences in the abundance of mammal- and bird-dispersed species for small woody stems and herbaceous plants were small and inconsequential as minor or no differences in the occurrence of other dispersal modes were noted. These results denote that the recruitment of zoochorous species is not lagging behind in more defaunated sites despite the reduction in disperser abundance. A plausible scenario is that dispersal, growth, and survival for young stems of zoochorous species are similarly successful in all sites, and this possibility may explain the observed patterns. Bearing in mind the limitations mentioned before, these findings do not support the statement that a reduction in large- and medium-sized mammals should translate to a decreased abundance of mammal-dispersed plants and a change in dispersal trait composition. Results from this study corroborate the findings of Chaves et al. (2015) in the Lancandona rainforest, Mexico, where no differences in the dispersal mode composition were found between forest fragments occupied or unoccupied by primates [69]. However, most research in the tropics suggests a clear decrease in the abundance of large-seeded mammal- and bird-dispersed species and often an increased abundance of other dispersal modes as a result of defaunation [5, 32, 33, 67, 71]. Additionally, several studies have shown that species with small or large seeds can be differently affected by defaunation [33, 39, 41, 67, 70, 85, 86]. Hence, methodological differences may partly explain the contrasting results, given that this study did not differentiate between small- and large-seeded species dispersed by mammals. Another methodological issue in the present study is that dispersal modes were only identified for a relatively small proportion of species encountered in the plots, due to the limited literature about these species. To get a more precise picture of plant trait response and composition for African tropical forest species, future studies should factor in differences in seed size and comprehensively identify plant functional traits. These limitations, however, do not invalidate observed trends for zoochorous species along the defaunation gradient because the overall abundance of young stems under parent trees of five mammal-dispersed species did not vary along the gradient. Consequently, one can gather that defaunation is not always accompanied by a modification of functional trait occurrence within communities of young understorey plants.

## 5. Conclusion

To sum up, there was no clear indication that understorey stem density, species diversity, or species composition were influenced by the differing levels of defaunation of the study sites. In spite of defaunation, the abundance of seedling and juvenile stems remained unaffected, and their spatial distribution was not compromised. The differing levels of defaunation did not appear to change the occurrence of dispersal modes. This study has contributed to the understanding of defaunation consequences by assessing the community structure, demographic structure, and functional trait composition of understorey plants in relatively biodiverse, Afrotropical sites and taking into account the role of extant taxa. Moreover, this study suggests that human activity may potentially function as an ecological force shaping processes underpinning the reproductive cycle of plants in defaunated forests, although the wider-reaching and longer-term impact of this role should be further studied and considered. In this study, greater defaunation did not result in a clear-cut change of the structure and traits of plant communities, suggesting that ecological functions of extinct fauna were compensated within the timeframe considered and among the levels of defaunation provided by the study sites. Functional compensation, therefore, is not an unlikely outcome of contemporary defaunation, and careful attention should be given to this possibility, particularly in areas with a high diversity of animal species and less partitioned niches. Ultimately, the rate of decrease in animal population size and the identity of extant animal species determine how defaunation will play out. The study also suggests that including the role of humans in conceptual models of plant community dynamics for defaunated areas may modify ecological outcomes expected in classical scenarios (like, for example, the Janzen-Connell model). Hence, forecasts of defaunation consequences that are based solely on extinct animals do not reflect what is happening in the real world in many wild places especially given increasing levels of human encroachment. Finally, the findings underline that the role played by small- and medium-sized animals persisting in defaunated areas is non-negligible and should not be overlooked. To maintain ecological function of defaunated forests, it may be important to consider the effective conservation of extant, and perhaps less iconic, animal species.

## Supporting information

S1 Checklist Inclusivity in global research. (DOCX)

S1 File Mammal abundance. (DOCX)

S2 File Site characteristics. (DOCX)

S3 File Conspecific density and demographics near focal trees–species-specific differences. (DOCX)

S1 Fig Additional figures. (DOCX)

S1 Table Additional tables. (DOCX)

S1 Data (XLSX)

S2 Data (XLSX)
