# Correction: Defaunation is known to have pervasive, negative effects on tropical forests, but this is not the whole story

**DOI:** 10.1371/journal.pone.0314411

**Published:** 2024-11-19

**Authors:** Gust Boiten, Steffi Dekegel, Nikki Tagg, Jacob Willie

There are errors in the Author Contributions. The correct contributions are:

**Conceptualization:** Nikki Tagg, Jacob Willie.

**Data curation:** Gust Boiten, Steffi Dekegel.

**Formal analysis:** Gust Boiten, Steffi Dekegel.

**Funding acquisition:** Nikki Tagg, Jacob Willie.

**Investigation:** Gust Boiten, Steffi Dekegel.

**Methodology:** Nikki Tagg, Jacob Willie.

**Project administration:** Jacob Willie.

**Resources:** Nikki Tagg.

**Software:** Gust Boiten, Jacob Willie.

**Supervision:** Nikki Tagg, Jacob Willie.

**Validation:** Gust Boiten, Jacob Willie.

**Visualization:** Gust Boiten.

**Writing–original draft:** Gust Boiten, Steffi Dekegel.

**Writing–review & editing:** Gust Boiten, Jacob Willie.
