# Supplementary material for: Defaunation is known to have pervasive, negative effects on tropical forests, but this is not the whole story
Source: PLoS One. 2023 Aug 31;18(8):e0290717. doi: 10.1371/journal.pone.0290717 (PMC10470957; doi:10.1371/journal.pone.0290717)
Supplement: S1 File — (DOCX) [file pone.0290717.s002.docx]

# Supplementary information 1: Mammal abundance.

## Mammal abundance sampling designs

Mammal abundance data was collected at six forest sites in the north-eastern part of the Dja Conservation Complex (Fig. 1) which consists of the core Dja Biosphere Reserve together with community forests, agricultural land, logging concessions, mines, and rural settlements on its periphery [1]. The six localities differ in terms of land-use type and level of active management and include two non-protected forest sites with no active management (Koum and Ngouleminanga), two non-protected forest sites but actively managed by Project Grand Singes (La Belgique and Palestine), and two officially protected forest sites (Schouam and Ndengue).

Figure 1: Location of the six study sites Koum, Ngouleminanga, La Palestine, La Belgique, Ndengue and Schouam in the north-eastern part Dja Conservation Complex, southeast Cameroon. Insert graph shows the location of the Dja Biosphere Reserve in Cameroon. Data source for the base files: https://cmr.forest-atlas.org/ [2–4].

Within each site, parallel transects of 6 km were cut with a distance of 500 m between transects. Five transects were opened in Koum, Ngouleminanga, La Palestine, Ndengue, and Schouam with a constant compass bearing of 180°, 140°, 325°, 315°, and 270°, respectively. Four transects of 6 km were opened in La Belgique with a constant compass bearing of 45°. Due to geographical barriers, it was not possible to finish every 6 km transects, especially in La Palestine. Therefore, two additional transects were cut in La Palestine in order to have approximately the same amount of total transect length in each site (Fig. 1). As a result, a total of 22.90 km transects were surveyed in Koum, 25.95 km in Ngouleminanga, 22.95 km in La Palestine, 24.00 km in La Belgique, 25.90 km in Ndengue, and 24.05 km in Schouam. All transects were opened perpendicular to major water courses to ensure that all habitat types were sampled. They were marked with wooden survey sticks at 50 m intervals. Following transect opening, all sites were left undisturbed for at least 3 weeks before starting data collection to ensure wildlife could recover from possible disturbances.

All data were collected over a 6-week period in July and August 2017 during the short dry season for valid comparisons of the data. Three survey teams started in the morning and walked each transect once at a speed of approximately 1 km/h. The first team included one researcher and two local guides and focussed on collecting the indirect observations. The second team consisted of one research assistant with one guide fully trained by PGS and walked behind the first team on the same transect for the great ape nest surveys. Finally, the third team was made up of one researcher and 2 local guides and recorded all direct observations of wildlife along a different transect. Local guides were hired from neighbouring villages and had extensive hunting experience, knowledge in local animal signs, and experience in observing mammals in the forest.

### Direct wildlife observations

Any direct observation of mammals was recorded along the transects following line-transect distance sampling techniques. For each individual or group encountered, the direct distance from the observer to an individual or group centre was estimated visually and the sighting angle from the transect line was determined using an orientation compass. In addition, the location along the transect (m), the observed species, the number of individuals seen, the habitat type, the canopy openness, the understory openness (open, average, dense), and the horizontal visibility (m) were recorded for each observation. Researchers occasionally left the transect to confirm group size, but all individuals or groups were initially detected from the transect line.

### Indirect wildlife observations

All indirect signs of animal activity were recorded using the fixed-width strip-transect technique. Evidence of animal presence such as feeding remains, vocalization, sleeping site, dung, footprints, and tracks were recorded within a distance of 1 m on each side of the transect line. For each observation, the location along the transect (m), habitat type, canopy openness, understory openness, and horizontal visibility (m) were determined. Whenever a dung was found along the transect, the perpendicular distance from the transect line to each dung was estimated. Most species could be identified based on their signs. However, due to difficulties in discriminating dung pellets among black-fronted duiker (*Cephalophus nigrifrons*), Peters’ duiker (*Cephalophus callipygus*), and bay duiker (*Cephalophus dorsalis*), these species were grouped as red duikers [5,6]. Also, indirect signs of squirrels could not be differentiated amongst species and were therefore grouped. The same was done for indirect signs of murines.

### Great ape nest surveys

Gorilla and chimpanzee nests were surveyed using the standing crop nest count methodology. This method is based on line-transect distance sampling and is a widely used approach to estimate great ape population sizes [7,8]. Ape surveys almost always rely on counts of sleeping nests, rather than on direct observations as these animals occur at low densities and visibility is low in dense tropical forests. All weaned great apes build nests that function as sleeping or resting platforms, which may persist for several months providing means of surveying great ape populations over large spatial scales [7].

When a nest was detected, an area of 30m radius was searched for additional nests. All nests of similar age category located within 20 m or 30 m for gorillas and chimpanzees respectively, were considered as belonging to the same group. These criteria were used to allow comparison with previous surveys in La Belgique [9].

For each encountered nest, several parameters were scored such as the type of nest construction, nest diameter (cm), and nest age category. The habitat type, canopy openness, understory openness, horizontal visibility (m) were determined. Also, the location along the transect (m) and the perpendicular distance from the transect line to the visible nest (m) were measured, and the GPS location was recorded.

For each tree nest, additional information on nest height (m), tree species, tree height (m), and tree DBH (cm) was recorded. While it is commonly accepted that chimpanzees build almost exclusively arboreal nests, previous research in La Belgique demonstrates that chimpanzees occasionally build nest on the ground for sleeping [10]. Furthermore, according to Willie et al. (2014), a small percent (3.8%) of the western lowland gorillas in La Belgique build their nest in trees [11]. This can complicate the differentiation of nests between the sympatric species. Therefore, additional nest characteristics and evidence such as hairs, odour, knuckle prints, and faeces were used to reliably confirm nest builder identity.

## Data analysis

First, transects within each site were divided into 600 m segments and every other segment was included in the analysis to obtain independent observation units [1].

To evaluate differences in mammal abundance across study sites, species encounter rate (number of signs per km) was used as index of abundance and was calculated for each 600 m segment. Analyses were conducted on species level and on diverse assemblages of species within the mammal community. Species were pooled into seven taxonomical guilds: 1) even-toed ungulates, 2) rodents, 3) carnivores, 4) pangolins, 5) elephants, 6) great apes, and 7) Old World monkeys. Two species (southern tree hyrax and giant otter shrew) could not be assigned to guilds. Indirect wildlife data were used for the first five guilds whereas nest counts where used to estimate great ape abundance. Individual great ape nests were used as a measurement instead of nest sites to avoid possible bias arising from grouping individual nests into nest sites. Direct observations were used to generate abundance estimates for arboreal monkeys based on the total number of individuals observed per segment [1,5,10]. Using this approach, the same species or guilds could be compared based on indices measured in the same way. As accurate density estimations require a minimum of 60-80 observations for each species, species densities (individuals per km²) could not be calculated since the number of observations for each species was not high enough.

## Statistical analysis

Statistical analyses were performed using R Studio version 3.4.3 [12]. The normality of the wildlife data was tested using a Shapiro-Wilk’s test and the Levene’s test was used to test for homogeneity of variances. Data were not normally distributed and showed heteroscedasticity. Therefore, a non-parametric Kruskal-Wallis test was used to perform between-site comparison of species abundances. The Dunn’s multiple comparison test with Benjamini-Hochberg correction was used as post-hoc analysis to determine which study sites differed significantly.

# Results

## Mammal abundance

During the surveys, a mammal assemblage of 37 species were detected across sites with 23 species observed in Koum, 18 species in Ngouleminanga, 21 species in Palestine, 24 species in La Belgique, 23 species in Schouam and 26 species in Ndengue.

There was a significant difference in mammal abundance across sites (Table 1 & Table 2; χ² = 86.18, P < 0.001). Despite its unprotected status, La Belgique showed the highest overall mammal abundance, followed by the officially protected site Ndengue. The post-hoc analysis revealed that mammal abundance in La Belgique was significantly higher compared to La Palestine and Schouam (both P < 0.01), however, it did not significantly differ from mammal abundance in Ndengue. Koum and Ngouleminanga showed the lowest mammal abundance compared to Palestine, La Belgique, Schouam, and Ndengue (all P < 0.001). No difference in mammal abundance was found between the study sites Palestine, Schouam, and Ndengue, and the same was observed between Koum and Ngouleminanga.

Analyses were conducted on encounter rates at species level and on taxonomic guild level (Table 1 & Table 2). All mammal guilds showed differences in abundance between study sites, except for Old World monkeys. Carnivores where significantly more frequently encountered in Koum and La Belgique than in Ngouleminanga (P < 0.05 and P < 0.01, respectively) and Schouam (both P < 0.05). Ndengue and La Belgique showed significantly higher abundance of pangolins compared with Koum (both P < 0.001), Ngouleminanga (P < 0.05 and P < 0.001, respectively), and Schouam (P < 0.05 and P < 0.001, respectively). Furthermore, rodents were significantly more abundant in Palestine than in any other study site, with the exception of La Belgique. In Ngouleminanga and La Belgique, rodents were significantly more abundant than in the officially protected sites Schouam (both P < 0.05) and Ndengue (both P < 0.01), however, no difference in rodent abundance was observed with Koum. Finally, even-toed ungulates were significantly more abundant in La Belgique and Ndengue than in any other study sites whereas Koum and Ngouleminanga showed the lowest ungulate abundance compared with any other study site.

Species-specific analysis demonstrated that not all mammal species significantly differed in abundance across study sites (Table 1 & Table 2). Species with high conservation value such as forest elephant, western lowland gorilla, and central chimpanzee showed significant differences in abundance between study sites (Table 1; χ2 = 66.92, P < 0.001; χ2 =19.00, P < 0.01; χ2 = 19.57, P < 0.01 respectively). Forest elephants were only encountered in La Belgique, Schouam, and Ndengue. The species was the most abundant in Schouam compared with La Belgique (P < 0.001) and Ndengue (P < 0.05), where abundances were similar. Central chimpanzees and western lowland gorilla nests were not encountered in Koum, Ngouleminanga, and Palestine. Moreover, gorilla nests were also not observed in Schouam, despite its officially protected status. Central chimpanzee nests were evenly abundant in La Belgique and the officially protected sites Schouam and Ndengue and were significantly more abundant in the officially protected site Ndengue compared to the unprotected sites Koum (P < 0.05), Ngouleminanga (P < 0.05), and Palestine (P < 0.01). Finally, western lowland gorillas were evenly abundant in La Belgique and the officially protected site Ndengue and were significantly more abundant in these two sites compared with any other site, including the officially protected site Schouam (all P < 0.05.

Some mammal species were only encountered in specific sites (Table 1). The southern tree hyrax and the black-legged mongoose were only recorded in Ngouleminanga, the African civet in Koum and the De Brazza’s monkey in Palestine, whereas the African buffalo was only spotted in Schouam. Finally, the water chevrotain was only observed in Ndengue.

Table 1: Species-specific encounter rates (and standard error) and mammal guild encounter rates (and standard error) per study site. Kruskal wallis test was used to test to test differences in encounter rates across study sites. Significant differences between sites are displayed: *P < 0.05; **P < 0.01; ***P < 0.001. Index of Abundance (km-1).

| Index of Abundance (km-1) | | | | | | | | | | | | | | | |
| --- | --- | --- | --- | --- | --- | --- | --- | --- | --- | --- | --- | --- | --- | --- | --- |
|  | | | Koum | | Ngouleminanga | | Palestine | | Belgique | | Schouam | | Ndengue | | Kruskal wallis test |
| Common Name | Scientific Name | Observation | Mean | SE | Mean | SE | Mean | SE | Mean | SE | Mean | SE | Mean | SE | (d.f. = 5) |
| Mammals | Mammalia | Indirect | 21.92 | 2.21 | 23.65 | 1.85 | 57.72 | 5.77 | 107.00 | 6.24 | 55.42 | 3.24 | 92.78 | 12.13 | χ2 = 86.18; P < 0.001*** |
| Forest Elephant | *Loxodonta cyclotis* | Indirect | 0.00 | 0.00 | 0.00 | 0.00 | 0.00 | 0.00 | 2.33 | 0.92 | 13.50 | 3.36 | 3.17 | 0.94 | χ2 = 66.92; P < 0.001*** |
| Giant Otter Shrew | *Potamogale velox* | Indirect | 0.08 | 0.08 | 0,00 | 0,00 | 0,09 | 0,09 | 0,00 | 0,00 | 0,00 | 0,00 | 0,00 | 0,00 | χ2 = 4.24; P = 0.515 |
| Southern Tree Hyrax | *Dendrohyrax arboreus* | Indirect | 0.00 | 0.00 | 0.08 | 0.08 | 0.00 | 0.00 | 0.00 | 0.00 | 0.00 | 0.00 | 0.00 | 0.00 | χ2 = 4.76; P = 0.446 |
| Carnivores | Carnivora | Indirect | 1.17 | 0.30 | 0.16 | 0.16 | 0.88 | 0.37 | 1.08 | 0.25 | 0.33 | 0.26 | 0.63 | 0.27 | χ2 = 19.67; P = 0.001** |
| African Civet | *Civettictis civetta* | Indirect | 0.17 | 0.11 | 0.00 | 0.00 | 0.00 | 0.00 | 0.00 | 0.00 | 0.00 | 0.00 | 0.00 | 0.00 | χ2 = 10.18; P=0.070 |
| African Clawless Otter | *Aonyx capensis* | Indirect | 0.00 | 0.00 | 0.00 | 0.00 | 0.18 | 0.12 | 0.00 | 0.00 | 0.00 | 0.00 | 0.24 | 0.13 | χ2 = 10.83; P= 0.055 |
| Servaline Genet | *Genetta servalina* | Indirect | 0.00 | 0.00 | 0.00 | 0.00 | 0.00 | 0.00 | 0.25 | 0.14 | 0.00 | 0.00 | 0.08 | 0.08 | χ2 = 11.34; P = 0.045* |
| Black-legged Mongoose | *Bdeogale nigripes* | Indirect | 0.00 | 0.00 | 0.08 | 0.08 | 0.00 | 0.00 | 0.00 | 0.00 | 0.00 | 0.00 | 0.00 | 0.00 | χ2 = 4.76; P = 0.446 |
| African Palm Civet | *Nandinia binotata* | Indirect | 0.00 | 0.00 | 0.08 | 0.08 | 0.26 | 0.19 | 0.08 | 0.08 | 0.00 | 0.00 | 0.00 | 0.00 | χ2 = 5.52; P = 0.356 |
| Long-nosed Mongoose | *Herpestes naso* | Indirect | 0.00 | 0.00 | 0.00 | 0.00 | 0.00 | 0.00 | 0.08 | 0.08 | 0.00 | 0.00 | 0.00 | 0.00 | χ2 = 5.05; P = 0.410 |
| Flat-headed Cusimanse | *Crossarchus platycephalus* | Indirect | 1.00 | 0.31 | 0.00 | 0.00 | 0.44 | 0.21 | 0.67 | 0.22 | 0.33 | 0.26 | 0.32 | 0.19 | χ2 = 16.29; P = 0.006** |
| Even-toed Ungulates | Artiodactyla | Indirect | 6.08 | 1.04 | 5.95 | 0.92 | 26.23 | 3.48 | 75.58 | 5.45 | 26.33 | 2.64 | 66.83 | 10.59 | χ2 = 93.72; P < 0.001*** |
| Large Ungulates (> 40kg) | | Indirect | 0.17 | 0.11 | 1.35 | 0.27 | 7.72 | 1.39 | 13.83 | 2.13 | 8.17 | 1.11 | 8.81 | 1.86 | χ2 = 66.50; P < 0.001*** |
| Forest Buffalo | *Syncerus caffer nanus* | Indirect | 0.00 | 0.00 | 0.00 | 0.00 | 0.00 | 0.00 | 0.00 | 0.00 | 0.33 | 0.26 | 0.00 | 0.00 | χ2 = 10.18; P = 0.070 |
| Yellow-backed Duiker | *Cephalophus silvicultor* | Indirect | 0.08 | 0.08 | 1.27 | 0.25 | 4.65 | 0.87 | 5.58 | 1.25 | 4.25 | 0.88 | 6.11 | 1.61 | χ2 = 40.90; P < 0.001*** |
| Sitatunga | *Tragelaphus spekii* | Indirect | 0.08 | 0.08 | 0.08 | 0.08 | 1.32 | 0.49 | 1.83 | 0.72 | 0.42 | 0.34 | 0.71 | 0.29 | χ2 = 14.93; P = 0.011* |
| Red River Hog | *Potamochoerus porcus* | Indirect | 0.00 | 0.00 | 0.00 | 0.00 | 1.75 | 0.68 | 6.42 | 1.22 | 3.17 | 0.59 | 1.98 | 0.64 | χ2 = 56.00; P < 0.001*** |
| Small ungulates (< 40kg) | | Indirect | 5.92 | 1.04 | 4.60 | 0.84 | 18.51 | 2.40 | 61.75 | 4.67 | 18.17 | 2.32 | 58.02 | 9.27 | χ2 = 92.35; P < 0.001*** |
| Blue Duiker | *Cephalophus monticola* | Indirect | 3.67 | 0.69 | 0.95 | 0.27 | 4.30 | 0.78 | 15.42 | 2.05 | 7.25 | 0.96 | 15.32 | 2.85 | χ2 = 68.87; P < 0.001*** |
| Water Chevrotain | *Hyemoschus aquaticus* | Indirect | 0.00 | 0.00 | 0.00 | 0.00 | 0.00 | 0.00 | 0.00 | 0.00 | 0.00 | 0.00 | 0.24 | 0.24 | χ2 = 4.76; P = 0.446 |
| Red duikers | Cephalophus sp*.* | Indirect | 2.25 | 0.44 | 3.65 | 0.78 | 14.21 | 2.10 | 0.00 | 3.33 | 10.92 | 1.86 | 42.46 | 6.70 | χ2 = 91.40; P < 0.001*** |
| Pangolins | Pholidota | Indirect | 0.42 | 0.17 | 1.19 | 0.37 | 2.11 | 0.28 | 3.58 | 0.85 | 0.92 | 0.26 | 4.84 | 0.70 | χ2 = 42.89; P < 0.001*** |
| Giant Pangolin | *Smutsia gigantea* | Indirect | 0.00 | 0.00 | 0.00 | 0.00 | 0.09 | 0.09 | 1.92 | 0.57 | 0.42 | 0.17 | 2.14 | 0.43 | χ2 = 47.58; P <0.001*** |
| Tree Pangolin | *Phataginus tricuspis* | Indirect | 0.42 | 0.17 | 1.19 | 0.37 | 2.02 | 0.27 | 1.67 | 0.50 | 0.50 | 0.21 | 2.70 | 0.60 | χ2 = 25.88; P < 0.001*** |
| Old World monkeys | Cercopithecidae | Direct | 1.00 | 1.00 | 0.95 | 0.95 | 2.63 | 1.57 | 4.25 | 2.64 | 1.75 | 0.77 | 4.84 | 1.82 | χ2 = 10.30; P = 0.067 |
| Eastern Putty-nosed Monkey | *Cercopithecus nictitans nictitans* | Direct | 0.33 | 0.33 | 0.32 | 0.32 | 0.00 | 0.00 | 0.00 | 0.00 | 0.92 | 0.49 | 0.79 | 0.79 | χ2 = 9.46; P = 0.092 |
| Crowned Monkey | *Cercopithecus pogonias* | Direct | 0.67 | 0.67 | 0.63 | 0.63 | 0.00 | 0.00 | 1.58 | 1.42 | 0.67 | 0.46 | 2.06 | 1.10 | χ2 = 7.98; P = 0.157 |
| Agile Mangabey | *Cercocebus agilis* | Direct | 0.00 | 0.00 | 0.00 | 0.00 | 0.00 | 0.00 | 0.17 | 0.17 | 0.00 | 0.00 | 0.40 | 0.28 | χ2 = 6.84; P = 0.233 |
| De Brazza’s Monkey | *Cercopithecus neglectus* | Direct | 0.00 | 0.00 | 0.00 | 0.00 | 0.44 | 0.44 | 0.00 | 0.00 | 0.00 | 0.00 | 0.00 | 0.00 | χ2 = 5.37; P = 0.373 |
| Red-tailed Mustached Monkey | *Cercopithecus cephus cephus* | Direct | 0.00 | 0.00 | 0.00 | 0.00 | 2.19 | 1.54 | 2.50 | 1.27 | 0.17 | 0.17 | 0.48 | 0.40 | χ2 = 8.54; P = 0.129 |
| Unidentified species | | Direct | 0.00 | 0.00 | 0.00 | 0.00 | 0.00 | 0.00 | 0.00 | 0.00 | 0.00 | 0.00 | 1.11 | 0.77 | χ2 = 9.60; P = 0.087 |
| Great apes | Hominidae | Nests | 0.00 | 0.00 | 0.00 | 0.00 | 0.00 | 0.00 | 1.50 | 0.58 | 0.92 | 0.52 | 2.14 | 0.62 | χ2 = 31.31; P < 0.001*** |
| Central Chimpanzee | *Pan troglodytes troglodytes* | Nests | 0.00 | 0.00 | 0.00 | 0.00 | 0.00 | 0.00 | 0.67 | 0.35 | 0.92 | 0.52 | 1.35 | 0.56 | χ2 = 19.57; P = 0.002** |
| Western Lowland Gorilla | *Gorilla gorilla gorilla* | Nests | 0.00 | 0.00 | 0.00 | 0.00 | 0.00 | 0.00 | 0.83 | 0.52 | 0.00 | 0.00 | 0.79 | 0.37 | χ2 = 19.00; P = 0.002** |
| Rodents | Rodentia | Indirect | 13.50 | 1.81 | 16.19 | 1.37 | 28.07 | 3.59 | 17.75 | 1.48 | 11.17 | 1.63 | 9.76 | 1.54 | χ2 = 38.10; P < 0.001*** |
| Large rodents (> 1kg) | | Indirect | 10.42 | 1.45 | 13.41 | 1.09 | 24.04 | 3.28 | 14.33 | 1.27 | 10.58 | 1.65 | 8.33 | 1.48 | χ2 = 33.70; P < 0.001*** |
| Forest Giant Pouched Rat | *Cricetomys emini* | Indirect | 3.58 | 0.50 | 5.24 | 0.55 | 5.70 | 1.03 | 2.08 | 0.48 | 3.33 | 0.65 | 1.11 | 0.27 | χ2 = 31.16; P < 0.001*** |
| African Brush-tailed Porcupine | *Atherurus africanus* | Indirect | 6.17 | 1.32 | 8.02 | 0.84 | 18.33 | 2.82 | 12.25 | 1.35 | 7.25 | 1.27 | 7.22 | 1.51 | χ2 = 29.49; P < 0.001*** |
| Greater Cane Rat | *Thryonomys swinderianus* | Indirect | 0.67 | 0.33 | 0.16 | 0.16 | 0.00 | 0.00 | 0.00 | 0.00 | 0.00 | 0.00 | 0.00 | 0.00 | χ2 = 16.04; P < 0.007** |
| Small rodents (< 1 kg) | | Indirect | 3.08 | 0.68 | 2.78 | 0.68 | 4.04 | 0.99 | 3.42 | 0.66 | 0.58 | 0.22 | 1.43 | 0.31 | χ2 = 22.34; P < 0.001*** |
| Squirrels | Sciuridae | Indirect | 1.42 | 0.35 | 0.16 | 0.11 | 1.23 | 0.38 | 0.00 | 0.00 | 0.33 | 0.19 | 0.24 | 0.13 | χ2 = 28.69; P < 0.001*** |
| Murines | Murinae | Indirect | 1.67 | 0.57 | 2.62 | 0.68 | 2.81 | 0.86 | 3.42 | 0.66 | 0.25 | 0.14 | 1.19 | 0.29 | χ2 = 23.95; P < 0.001*** |

Table 2: Outcomes of the Dunn’s multiple comparison test for between-site differences in species-level and taxonomic guild abundances. Significant differences between sites are displayed: *P<0.05; **P < 0.01; ***P < 0.001. The six study sites are abbreviated as K: Koum; NG: Ngouleminanga; PA: Palestine; LB: La Belgique; S: Schouam; ND: Ndengue

| Multiple comparison | | | | | | | | | | | | | | | | |
| --- | --- | --- | --- | --- | --- | --- | --- | --- | --- | --- | --- | --- | --- | --- | --- | --- |
| Common Name | Scientific Name | K-NG | K-PA | K-LB | K-S | K-ND | NG-PA | NG-LB | NG-S | NG-ND | PA-LB | PA-S | PA-ND | LB-S | LB-ND | S-ND |
| Mammals | Mammalia |  | *** | *** | *** | *** | *** | *** | *** | *** | ** |  |  | ** |  |  |
| Forest Elephant | *Loxodonta cyclotis* |  |  | * | *** | *** |  | * | *** | *** | * | *** | *** | *** |  | * |
| Giant Otter Shrew | *Potamogale velox* |  |  |  |  |  |  |  |  |  |  |  |  |  |  |  |
| Southern Tree Hyrax | *Dendrohyrax arboreus* |  |  |  |  |  |  |  |  |  |  |  |  |  |  |  |
| Carnivores | Carnivora | * |  |  | * |  |  | ** |  |  |  |  |  | * |  |  |
| African Civet | *Civettictis civetta* |  |  |  |  |  |  |  |  |  |  |  |  |  |  |  |
| African Clawless Otter | *Aonyx capensis* |  |  |  |  |  |  |  |  |  |  |  |  |  |  |  |
| Servaline Genet | *Genetta servalina* |  |  | * |  |  |  | * |  |  | * |  |  | * |  |  |
| Black-legged Mongoose | *Bdeogale nigripes* |  |  |  |  |  |  |  |  |  |  |  |  |  |  |  |
| African Palm Civet | *Nandinia binotata* |  |  |  |  |  |  |  |  |  |  |  |  |  |  |  |
| Long-nosed Mongoose | *Herpestes naso* |  |  |  |  |  |  |  |  |  |  |  |  |  |  |  |
| Flat-headed Cusimanse | *Crossarchus platycephalus* | ** |  |  | * |  |  | * |  |  |  |  |  |  |  |  |
| Even-toed Ungulates | Artiodactyla |  | *** | *** | *** | *** | *** | *** | *** | *** | *** |  | ** | *** |  | * |
| Large Ungulates (> 40kg) | |  | *** | *** | *** | *** | *** | *** | *** | *** |  |  |  |  |  |  |
| Forest Buffalo | *Syncerus caffer nanus* |  |  |  |  |  |  |  |  |  |  |  |  |  |  |  |
| Yellow-backed Duiker | *Cephalophus silvicultor* |  | *** | *** | *** | *** | * | ** | * | * |  |  |  |  |  |  |
| Sitatunga | *Tragelaphus spekii* |  | * | * |  |  | * | * |  |  |  |  |  |  |  |  |
| Red River Hog | *Potamochoerus porcus* |  | * | *** | *** | ** | * | *** | *** | ** | ** | * |  |  | ** |  |
| Small ungulates (< 40kg) | |  | ** | *** | ** | *** | *** | *** | *** | *** | *** |  | ** | *** |  | ** |
| Blue Duiker | *Cephalophus monticola* | * |  | *** | * | *** | * | *** | *** | *** | *** |  | *** | * |  |  |
| Water Chevrotain | *Hyemoschus aquaticus* |  |  |  |  |  |  |  |  |  |  |  |  |  |  |  |
| Red duikers | Cephalophus sp*.* |  | *** | *** | ** | *** | ** | *** | * | *** | *** |  | ** | *** |  | *** |
| Pangolins | Pholidota |  | ** | *** |  | *** |  | * |  | *** |  | * |  | * |  | *** |
| Giant Pangolin | *Smutsia gigantea* |  |  | *** |  | *** |  | *** |  | *** | *** |  | *** | * |  | ** |
| Tree Pangolin | *Phataginus tricuspis* |  | ** |  |  | ** |  |  |  |  |  | ** |  |  |  | ** |
| Old World monkeys | Cercopithecidae |  |  |  |  |  |  |  |  |  |  |  |  |  |  |  |
| Eastern Putty-nosed Monkey | *Cercopithecus nictitans nictitans* |  |  |  |  |  |  |  |  |  |  |  |  |  |  |  |
| Crowned Monkey | *Cercopithecus pogonias* |  |  |  |  |  |  |  |  |  |  |  |  |  |  |  |
| Agile Mangabey | *Cercocebus agilis* |  |  |  |  |  |  |  |  |  |  |  |  |  |  |  |
| De Brazza’s Monkey | *Cercopithecus neglectus* |  |  |  |  |  |  |  |  |  |  |  |  |  |  |  |
| Red-tailed Mustached Monkey | *Cercopithecus cephus cephus* |  |  |  |  |  |  |  |  |  |  |  |  |  |  |  |
| Unidentified species | |  |  |  |  |  |  |  |  |  |  |  |  |  |  |  |
| Great apes | Hominidae |  |  | ** |  | *** |  | ** |  | *** | ** |  | *** |  |  |  |
| Central Chimpanzee | *Pan troglodytes troglodytes* |  |  |  |  | * |  |  |  | * |  |  | ** |  |  |  |
| Western Lowland Gorilla | *Gorilla gorilla gorilla* |  |  | * |  | * |  | * |  | * | * |  | * | * |  | * |
| Rodents | Rodentia |  | ** |  |  |  | * |  | * | ** |  | *** | *** | * | ** |  |
| Large rodents (> 1kg) | |  | *** |  |  |  |  |  |  | ** |  | *** | *** |  | ** |  |
| Forest Giant Pouched Rat | *Cricetomys emini* |  |  |  |  | ** |  | ** |  | *** | * |  | *** |  |  | * |
| African Brush-tailed Porcupine | *Atherurus africanus* |  | *** | ** |  |  | ** |  |  |  |  | ** | *** | * | ** |  |
| Greater Cane Rat | *Thryonomys swinderianus* | * | ** | ** | * | * |  |  |  |  |  |  |  |  |  |  |
| Small rodents (< 1 kg) | |  |  |  | ** |  |  |  | * |  |  | ** |  | ** |  |  |
| Squirrels | Sciuridae | ** |  | *** | ** | ** | ** |  |  |  | ** | * | * |  |  |  |
| Murines | Murinae |  |  | * |  |  |  |  | ** |  |  | ** |  | *** | * |  |

# References

1. Tagg N, Willie J, Duarte J, Petre C-A, Fa JE. Conservation research presence protects: a case study of great ape abundance in the Dja region, Cameroon. Anim Conserv. 2015;18: 489–498. doi:10.1111/acv.12212

2. Mba JE. Protected areas. The Forest Atlas of Cameroon; 2017. Available: https://data-minfof.opendata.arcgis.com/datasets/minfof::aires-proteg%C3%A9es-de-faunes/explore

3. Mba JE. Community forests. The Forest Atlas of Cameroon; 2018. Available: https://data-minfof.opendata.arcgis.com/datasets/minfof::community-forests/explore

4. Mba JE. Production Forests. The Forest Atlas of Cameroon; 2018. Available: https://data-minfof.opendata.arcgis.com/datasets/minfof::production-forests/explore

5. Laurance WF, Croes BM, Tchignoumba L, Lahm SA, Alonso A, Lee ME, et al. Impacts of Roads and Hunting on Central African Rainforest Mammals. Conserv Biol. 2006;20: 1251–1261. doi:10.1111/j.1523-1739.2006.00420.x

6. Van Vliet N, Zundel S, Miquel C, Taberlet P, Nasi R. Distinguishing dung from blue, red and yellow-backed duikers through noninvasive genetic techniques. Afr J Ecol. 2008;46: 411–417. doi:10.1111/j.1365-2028.2007.00879.x

7. Tutin CEG, Fernandez M. Nationwide census of gorilla (gorilla g. gorilla) and chimpanzee (Pan t. troglodytes) populations in Gabon. Am J Primatol. 1984;6: 313–336. doi:10.1002/ajp.1350060403

8. Thomas L, Buckland ST, Rexstad EA, Laake JL, Strindberg S, Hedley SL, et al. Distance software: design and analysis of distance sampling surveys for estimating population size. J Appl Ecol. 2010;47: 5–14. doi:10.1111/j.1365-2664.2009.01737.x

9. Dupain J, Guislain P, Nguenang GM, Vleeschouwer KD, Elsacker LV. High chimpanzee and gorilla densities in a non-protected area on the northern periphery of the Dja Faunal Reserve, Cameroon. Oryx. 2004;38: 209–216. doi:10.1017/S0030605304000365

10. Tagg N, Willie J, Petre C-A, Haggis O. Ground Night Nesting in Chimpanzees: New Insights from Central Chimpanzees (Pan troglodytes troglodytes) in South-East Cameroon. Folia Primatol (Basel). 2013;84: 362–383. doi:10.1159/000353172

11. Willie J, Tagg N, Petre C-A, Pereboom Z, Lens L. Plant selection for nest building by western lowland gorillas in Cameroon. Primates. 2014;55: 41–49. doi:10.1007/s10329-013-0363-5

12. R Core Team. R: A language and environment for statistical computing. Version 3.4.3. Vienna, Austria: R Foundation for Statistical Computing; 2017. Available: https://www.R-project.org/
