# Supplementary material for: Defaunation is known to have pervasive, negative effects on tropical forests, but this is not the whole story
Source: PLoS One. 2023 Aug 31;18(8):e0290717. doi: 10.1371/journal.pone.0290717 (PMC10470957; doi:10.1371/journal.pone.0290717)
Supplement: S2 File — (DOCX) [file pone.0290717.s003.docx]

## Supplementary information 3: Site characteristics.

# Site characteristics

## Species importance

Species importance was determined for all species recorded in the large plots. The importance values of species differed across sites (Table 1). Out of the twenty species with the highest importance values, six species (*Rinorea longifolia*, *Petersianthus macrocarpus*, *Tabernaemontana crassa*, *Uapaca paludosa*, *Alchornea floribunda*, and *Lasianthera africana*) were common at all sites. Most of the highest-ranking species in Ngouleminanga (most defaunated site) also predominated in at least one of the other sites. Only little more than half of the high-ranking species in La Belgique (least defaunated site) and Palestine (intermediate site) also predominated in at least one other site.

In La Belgique (LB) and Palestine (PA), the tree *Rinorea longifolia* was the most important woody plant (> 1m) (LB, IV = 16.29; PA, IV = 21.29). The species in Ngouleminanga (NG) had overall lower importance values. At this site the most important woody plant species was the tree *Petersianthus macrocarpus* (IV = 11.69). Palestine showed the biggest difference between the most important species and the lower ranking plants. La Belgique and especially Ngouleminanga (NG) showed a more gradual decline of importance values towards lower ranking species. Noteworthy was the high importance of the palm *Raphia monbuttorum* in La Belgique (IV = 9.23). La Belgique also showed high importance of one shrub species (*Lasianthera africana*, IV = 5.6) and three liana species (*Manniophyton fulvum*, IV = 5.12; *Tetracera podotricha*, IV = 4.65; *Neuropeltis acuminata*, IV = 4.33). In Palestine, two shrub species (*Lasianthera Africana*, IV = 10.4; *Pseuderanthemum ludovicianum*, IV = 5.12) and one liana (*Strychnos angolensis*, IV = 4.71) had high importance. In Ngouleminanga, only two species of shrubs (*Lasianthera africana*, IV = 9.83; *Pseuderanthemum ludovicianum*, IV = 6.75) and no lianas showed high importance. All other plants of the twenty most important species at the three sites were trees.

**Table 1: The importance values for the woody species in the assemblage of stems >1m.**

|  | La Belgique | | | | Palestine | | | | Ngouleminanga | | | |
| --- | --- | --- | --- | --- | --- | --- | --- | --- | --- | --- | --- | --- |
| Nr. | Species | Morphospecies | IV | % IV | Species | Morphospecies | IV | % IV | Species | Morphospecies | IV | % IV |
| 1 | *Rinorea longifolia* | Tree | 16.29 | 5.43 | *Rinorea longifolia* | Tree | 21.29 | 7.10 | *Petersianthus macrocarpus* | Tree | 11.69 | 3.90 |
| 2 | *Petersianthus macrocarpus* | Tree | 12.57 | 4.19 | *Tabernaemontana crassa* | Tree | 12.74 | 4.25 | *Lasianthera africana* | Shrub | 9.83 | 3.28 |
| 3 | *Tabernaemontana crassa* | Tree | 11.74 | 3.91 | *Uapaca paludosa* | Tree | 11.47 | 3.82 | *Pentaclethra macrophylla* | Tree | 9.63 | 3.21 |
| 4 | *Uapaca guineensis* | Tree | 10.39 | 3.46 | *Lasianthera africana* | Shrub | 10.40 | 3.47 | *Heisteria parvifolia* | Tree | 8.89 | 2.96 |
| 5 | *Uapaca paludosa* | Tree | 9.69 | 3.23 | *Heisteria parvifolia* | Tree | 10.21 | 3.40 | *Tabernaemontana crassa* | Tree | 8.89 | 2.96 |
| 6 | *Raphia monbuttorum* | Palm | 9.23 | 3.08 | *Distemonanthus benthamianus* | Tree | 9.71 | 3.24 | *Uapaca paludosa* | Tree | 8.52 | 2.84 |
| 7 | *Polyalthia suaveolens* | Tree | 8.29 | 2.76 | *Petersianthus macrocarpus* | Tree | 8.62 | 2.87 | *Pseuderanthemum ludovicianum* | Shrub | 6.75 | 2.25 |
| 8 | *Alchornea floribunda* | Tree | 7.7 | 2.57 | *Pentaclethra macrophylla* | Tree | 8.47 | 2.82 | *Distemonanthus benthamianus* | Tree | 6.48 | 2.16 |
| 9 | *Strombosia grandifolia* | Tree | 6.46 | 2.15 | *Trichilia heudelotii* | Tree | 8.09 | 2.70 | *Alchornea floribunda* | Tree | 5.96 | 1.99 |
| 10 | *Desbordesia glaucescens* | Tree | 6.23 | 2.08 | *Plagiostyles africana* | Tree | 7.71 | 2.57 | *Polyalthia suaveolens* | Tree | 5.58 | 1.86 |
| 11 | *Plagiostyles africana* | Tree | 5.74 | 1.91 | *Uapaca acuminata* | Tree | 7.45 | 2.48 | *Strombosia grandifolia* | Tree | 5.13 | 1.71 |
| 12 | *Lasianthera africana* | Shrub | 5.6 | 1.87 | *Santiria trimera* | Tree | 7.07 | 2.36 | *Rinorea dentata* | Tree | 5.00 | 1.67 |
| 13 | *Tricalysia oligoneura* | Tree | 5.44 | 1.81 | *Alchornea floribunda* | Tree | 6.76 | 2.25 | *Rinorea longifolia* | Tree | 4.83 | 1.61 |
| 14 | *Manniophyton fulvum* | Liana | 5.12 | 1.71 | *Carapa procera* | Tree | 6.20 | 2.07 | Trichilia sp. | Tree | 4.66 | 1.55 |
| 15 | *Strombosiopsis tetrandra* | Tree | 4.67 | 1.56 | *Pycnanthus angolensis* | Tree | 5.95 | 1.98 | *Celtis mildbraedii* | Tree | 4.50 | 1.50 |
| 16 | *Rinorea dentata* | Tree | 4.66 | 1.55 | *Coelocaryon preussii* | Tree | 5.51 | 1.84 | *Coelocaryon preussii* | Tree | 4.34 | 1.45 |
| 17 | *Tetracera podotricha* | Liana | 4.65 | 1.55 | *Pseuderanthemum ludovicianum* | Shrub | 5.12 | 1.71 | *Erythrophleum suaveolens* | Tree | 4.21 | 1.40 |
| 18 | Trichilia sp. | Tree | 4.46 | 1.49 | *Hylodendron gabunense* | Tree | 4.95 | 1.65 | *Uapaca vanhoutei* | Tree | 3.84 | 1.28 |
| 19 | *Neuropeltis acuminata* | Liana | 4.33 | 1.44 | *Strychnos angolensis* | Liana | 4.71 | 1.57 | *Musanga cecropioides* | Tree | 3.84 | 1.28 |
| 20 | *Lecaniodiscus cupanioides* | Tree | 3.96 | 1.32 | *Canarium wenzelii* | Tree | 4.67 | 1.56 | *Symphonia globulifera* | Tree | 3.79 | 1.26 |
| 21 | *Uapaca acuminata* | Tree | 3.9 | 1.3 | *Chytranthus mortehanii* | Tree | 4.59 | 1.53 | *Strombosiopsis tetrandra* | Tree | 3.76 | 1.25 |
| 22 | *Sorindeia grandifolia* | Tree | 3.51 | 1.17 | *Strombosia grandifolia* | Tree | 4.20 | 1.40 | *Carapa procera* | Tree | 3.66 | 1.22 |
| 23 | *Eribroma oblongum* | Tree | 3.42 | 1.14 | *Uapaca vanhoutei* | Tree | 4.20 | 1.40 | *Pterocarpus mildbraedii* | Tree | 3.50 | 1.17 |
| 24 | *Monodora myristica* | Tree | 3.23 | 1.08 | *Polyalthia suaveolens* | Tree | 3.95 | 1.32 | *Olax latifolia* | Tree | 3.42 | 1.14 |
| 25 | *Cola balayii* | Tree | 3.15 | 1.05 | *Uapaca guineensis* | Tree | 3.94 | 1.31 | *Chytranthus mortehanii* | Tree | 3.34 | 1.11 |
| 26 | *Dichapetalum sp* | Liana | 3.13 | 1.04 | Trichilia sp. | Tree | 3.75 | 1.25 | *Diospyros mannii* | Tree | 3.27 | 1.09 |
| 27 | *Enantia chlorantha* | Tree | 3.1 | 1.03 | *Symphonia globulifera* | Tree | 3.58 | 1.19 | *Antrocaryon klaineanum* | Tree | 3.08 | 1.03 |
| 28 | *Desplatsia subericarpa* | Tree | 3.1 | 1.03 | *Neuropeltis acuminata* | Liana | 3.13 | 1.04 | *Hylodendron gabunense* | Tree | 3.07 | 1.02 |
| 29 | *Pterocarpus mildbraedii* | Tree | 3.07 | 1.02 | *Strombosiopsis tetrandra* | Tree | 3.11 | 1.04 | *Ongokea gore* | Tree | 3.06 | 1.02 |
| 30 | *Anonidium mannii* | Tree | 3.03 | 1.01 | *Rothmannia coriacea* | Tree | 2.91 | 0.97 | *Unidentified tree 10* | Tree | 2.93 | 0.98 |
| 31 | *Trichilia heudelotii* | Tree | 2.97 | 0.99 | *Anonidium mannii* | Tree | 2.87 | 0.96 | *Centroplacus glaucinus* | Tree | 2.89 | 0.96 |
| 32 | *Psychotria densinervia* | Tree | 2.87 | 0.96 | *Celtis tessmannii* | Tree | 2.78 | 0.93 | *Santiria trimera* | Tree | 2.89 | 0.96 |
| 33 | *Pausinystalia lane-poolei* | Tree | 2.78 | 0.93 | *Centroplacus glaucinus* | Tree | 2.78 | 0.93 | *Staudtia kamerunensis* | Tree | 2.85 | 0.95 |
| 34 | *Olax latifolia* | Tree | 2.7 | 0.9 | *Olax latifolia* | Tree | 2.61 | 0.87 | *Gambeya boukokoensis* | Tree | 2.79 | 0.93 |
| 35 | *Massularia acuminata* | Tree | 2.7 | 0.9 | *Lecaniodiscus cupanioides* | Tree | 2.47 | 0.82 | *Trichilia heudelotii* | Tree | 2.66 | 0.89 |
| 36 | *Strychnos angolensis* | Liana | 2.68 | 0.89 | *Tetrapleura tetraptera* | Tree | 2.40 | 0.80 | *Uapaca guineensis* | Tree | 2.63 | 0.88 |
| 37 | *Strombosia pustulata* | Shrub | 2.67 | 0.89 | *Hugonia platysepala* | Liana | 2.39 | 0.80 | *Pterocarpus soyauxii* | Tree | 2.62 | 0.87 |
| 38 | *Aulacocalyx auriculata* | Tree | 2.52 | 0.84 | *Chytranthus atroviolaceus* | Tree | 2.37 | 0.79 | *Strychnos angolensis* | Liana | 2.60 | 0.87 |
| 39 | *Maesobotrya klaineana* | Tree | 2.46 | 0.82 | *Guarea cedrata* | Tree | 2.35 | 0.78 | *Amphimas pterocarpoides* | Tree | 2.56 | 0.85 |
| 40 | *Diospyros holeana* | Tree | 2.44 | 0.81 | *Tricalysia oligoneura* | Tree | 2.33 | 0.78 | *Panda oleosa* | Tree | 2.53 | 0.84 |
| 41 | *Duboscia macrocarpa* | Tree | 2.41 | 0.8 | *Unidentified tree 10* | Tree | 2.29 | 0.76 | *Raphia monbuttorum* | Palm | 2.52 | 0.84 |
| 42 | *Milicia excelsa* | Tree | 2.39 | 0.8 | *Landolphia violacea* | Liana | 2.24 | 0.75 | *Unidentified tree 11* | Tree | 2.47 | 0.82 |
| 43 | *Erythrophleum suaveolens* | Tree | 2.38 | 0.79 | *Rinorea dentata* | Tree | 2.03 | 0.68 | *Funtumia elastica* | Tree | 2.45 | 0.82 |
| 44 | *Terminalia superba* | Tree | 2.35 | 0.78 | *Maesobotrya sp* | Tree | 2.00 | 0.67 | *Brenania brieyi* | Tree | 2.43 | 0.81 |
| 45 | *Heisteria parvifolia* | Tree | 2.34 | 0.78 | *Enantia chlorantha* | Tree | 1.78 | 0.59 | *Rothmannia coriacea* | Tree | 2.41 | 0.80 |
| 46 | *Anthonotha macrophylla* | Tree | 2.31 | 0.77 | *Cleistopholis patens* | Liana | 1.71 | 0.57 | *Monodora myristica* | Tree | 2.35 | 0.78 |
| 47 | *Santiria trimera* | Tree | 2.15 | 0.72 | *Sorindeia grandifolia* | Tree | 1.65 | 0.55 | *Celtis tessmannii* | Tree | 2.29 | 0.76 |
| 48 | *Chytranthus mortehanii* | Tree | 2.02 | 0.67 | *Psychotria densinervia* | Tree | 1.64 | 0.55 | *Uapaca acuminata* | Tree | 2.20 | 0.73 |
| 49 | *Albizia zygia* | Tree | 1.98 | 0.66 | *Celtis mildbraedii* | Tree | 1.59 | 0.53 | *Diospyros holeana* | Tree | 2.19 | 0.73 |
| 50 | *Pterocarpus soyauxii* | Tree | 1.97 | 0.66 | *Pausinystalia lane-poolei* | Tree | 1.46 | 0.49 | *Albizia adianthifolia* | Tree | 2.19 | 0.73 |
| 51 | *Pentaclethra macrophylla* | Tree | 1.95 | 0.65 | *Anthonotha macrophylla* | Tree | 1.41 | 0.47 | *Maesobotrya sp* | Tree | 2.17 | 0.72 |
| 52 | *Chytranthus atroviolaceus* | Tree | 1.94 | 0.65 | *Lasiodiscus marmoratus* | Tree | 1.39 | 0.46 | Dichapetalum sp | Liana | 2.07 | 0.69 |
| 53 | *Campylospermum elongatum* | Shrub | 1.83 | 0.61 | *Brenania brieyi* | Tree | 1.38 | 0.46 | *Nauclea pobeguinii* | Tree | 1.92 | 0.64 |
| 54 | Maesobotrya sp | Tree | 1.82 | 0.61 | *Desbordesia glaucescens* | Tree | 1.32 | 0.44 | *Neuropeltis acuminata* | Liana | 1.92 | 0.64 |
| 55 | Combretum sp2 | Liana | 1.81 | 0.6 | *Diospyros holeana* | Tree | 1.30 | 0.43 | *Lecaniodiscus cupanioides* | Tree | 1.78 | 0.59 |
| 56 | *Syzygium guineense* | Tree | 1.78 | 0.59 | Draceana sp | Tree | 1.24 | 0.41 | *Landolphia violacea* | Liana | 1.76 | 0.59 |
| 57 | *Uapaca vanhoutei* | Tree | 1.75 | 0.58 | *Monodora myristica* | Tree | 1.24 | 0.41 | *Xylopia hypolampra* | Tree | 1.76 | 0.59 |
| 58 | *Rothmannia coriacea* | Tree | 1.74 | 0.58 | *Agelaea paradoxa* | Liana | 1.23 | 0.41 | *Desbordesia glaucescens* | Tree | 1.75 | 0.58 |
| 59 | *Hugonia platysepala* | Liana | 1.71 | 0.57 | *Garcinia punctata* | Tree | 1.18 | 0.39 | *Anonidium mannii* | Tree | 1.74 | 0.58 |
| 60 | *Hylodendron gabunense* | Tree | 1.69 | 0.56 | *Campylospermum elongatum* | Shrub | 1.16 | 0.39 | *Tricalysia oligoneura* | Tree | 1.69 | 0.56 |
| 61 | *Warneckea cinnamomoides* | Tree | 1.66 | 0.55 | *Tetracera podotricha* | Liana | 1.15 | 0.38 | *Macaranga barteri* | Tree | 1.62 | 0.54 |
| 62 | *Brenania brieyi* | Tree | 1.58 | 0.53 | *Antrocaryon klaineanum* | Tree | 1.13 | 0.38 | *Agelaea paradoxa* | Liana | 1.60 | 0.53 |
| 63 | *Cordia platythyrsa* | Tree | 1.52 | 0.51 | Indigofera sp | Liana | 1.07 | 0.36 | *Garcinia punctata* | Tree | 1.60 | 0.53 |
| 64 | *Celtis tessmannii* | Tree | 1.51 | 0.5 | *Strombosia pustulata* | Shrub | 1.07 | 0.36 | *Plagiostyles africana* | Tree | 1.58 | 0.53 |
| 65 | *Phyllanthus discoideus* | Tree | 1.49 | 0.5 | *Klainedoxa gabonensis* | Tree | 1.05 | 0.35 | *Piptadeniastrum africanum* | Tree | 1.54 | 0.51 |
| 66 | *Celtis mildbraedii* | Tree | 1.41 | 0.47 | *Manniophyton fulvum* | Liana | 1.04 | 0.35 | *Trichoscypha acuminata* | Tree | 1.50 | 0.50 |
| 67 | *Tetrapleura tetraptera* | Tree | 1.39 | 0.46 | *Warneckea cinnamomoides* | Tree | 1.04 | 0.35 | *Myrianthus arboreus* | Tree | 1.45 | 0.48 |
| 68 | Treculia sp | Tree | 1.27 | 0.42 | *Maesobotrya klaineana* | Tree | 1.01 | 0.34 | *Klainedoxa microphylla* | Tree | 1.42 | 0.47 |
| 69 | *Guarea thompsonii* | Tree | 1.24 | 0.41 | Dichapetalum sp | Liana | 0.99 | 0.33 | *Cola balayii* | Tree | 1.41 | 0.47 |
| 70 | *Desplatsia dewevrei* | Tree | 1.23 | 0.41 | *Amphimas pterocarpoides* | Tree | 0.99 | 0.33 | *Albizia zygia* | Tree | 1.40 | 0.47 |
| 71 | *Coelocaryon preussii* | Tree | 1.22 | 0.41 | *Myrianthus arboreus* | Tree | 0.98 | 0.33 | *Tabernaemontana penduliflora* | Tree | 1.36 | 0.45 |
| 72 | *Pteleopsis hylodendron* | Tree | 1.2 | 0.4 | Rinorea sp | Tree | 0.97 | 0.32 | *Anthonotha macrophylla* | Tree | 1.35 | 0.45 |
| 73 | *Cissus dinklagei* | Liana | 1.15 | 0.38 | *Trichoscypha acuminata* | Tree | 0.96 | 0.32 | *Voacanga africana* | Tree | 1.32 | 0.44 |
| 74 | *Landolphia violacea* | Liana | 0.99 | 0.33 | *Cola balayii* | Tree | 0.91 | 0.30 | *Xylopia rubescens* | Tree | 1.26 | 0.42 |
| 75 | *Bridelia micrantha* | Tree | 0.94 | 0.31 | Dicranolepsis sp | Shrub | 0.91 | 0.30 | *Alstonia boonei* | Tree | 1.23 | 0.41 |
| 76 | Rinorea sp | Tree | 0.9 | 0.3 | *Cissus dinklagei* | Liana | 0.82 | 0.27 | *Entandrophragma candollei* | Tree | 1.19 | 0.40 |
| 77 | *Sindoropsis le-testui* | Tree | 0.83 | 0.28 | Drypetes sp | Tree | 0.81 | 0.27 | *Chytranthus atroviolaceus* | Tree | 1.18 | 0.39 |
| 78 | *Macaranga barteri* | Tree | 0.82 | 0.27 | *Diospyros mannii* | Tree | 0.78 | 0.26 | *Sterculia tragacantha* | Tree | 1.16 | 0.39 |
| 79 | *Distemonanthus benthamianus* | Tree | 0.8 | 0.27 | *Rhabdophyllum calophyllum* | Tree | 0.77 | 0.26 | *Duboscia macrocarpa* | Tree | 1.14 | 0.38 |
| 80 | *Trichilia gilgiana* | Tree | 0.75 | 0.25 | *Agelaea pseudobliqua* | Liana | 0.77 | 0.26 | *Fagara dinklagei* | Tree | 1.09 | 0.36 |
| 81 | Indigofera sp | Liana | 0.74 | 0.25 | *Duboscia macrocarpa* | Tree | 0.77 | 0.26 | *Aulacocalyx auriculata* | Tree | 1.08 | 0.36 |
| 82 | *Oncoba crepiniana* | Tree | 0.7 | 0.23 | *Cola pachycarpa* | Tree | 0.72 | 0.24 | *Lepidobotrys staudtii* | Tree | 1.06 | 0.35 |
| 83 | Millettia sp | Liana | 0.68 | 0.23 | *Albizia adianthifolia* | Tree | 0.66 | 0.22 | *Sorindeia grandifolia* | Tree | 1.03 | 0.34 |
| 84 | *Symphonia globulifera* | Tree | 0.67 | 0.22 | *Aidia micrantha* | Tree | 0.65 | 0.22 | *Dacryodes buettneri* | Tree | 1.03 | 0.34 |
| 85 | *Diospyros crassiflora* | Tree | 0.66 | 0.22 | *Tabernaemontana penduliflora* | Tree | 0.65 | 0.22 | *Guarea thompsonii* | Tree | 1.01 | 0.34 |
| 86 | *Gambeya boukokoensis* | Tree | 0.66 | 0.22 | *Cola caricaefolia* | Tree | 0.65 | 0.22 | *Pausinystalia lane-poolei* | Tree | 0.97 | 0.32 |
| 87 | *Xylopia rubescens* | Tree | 0.63 | 0.21 | *Massularia acuminata* | Tree | 0.63 | 0.21 | *Irvingia grandifolia* | Tree | 0.96 | 0.32 |
| 88 | *Cola arcuata* | Tree | 0.63 | 0.21 | *Picralima nitida* | Tree | 0.63 | 0.21 | *Macaranga spinosa* | Tree | 0.95 | 0.32 |
| 89 | Uapaca sp | Tree | 0.6 | 0.2 | *Irvingia gabonensis* | Tree | 0.61 | 0.20 | *Albizia coriaria* | Tree | 0.89 | 0.30 |
| 90 | *Myrianthus arboreus* | Tree | 0.59 | 0.2 | *Gambeya boukokoensis* | Tree | 0.58 | 0.19 | *Agelaea pseudobliqua* | Liana | 0.88 | 0.29 |
| 91 | *Pseuderanthemum ludovicianum* | Shrub | 0.58 | 0.19 | *Pteleopsis hylodendron* | Tree | 0.58 | 0.19 | *Manniophyton fulvum* | Liana | 0.88 | 0.29 |
| 92 | *Garcinia mannii* | Tree | 0.58 | 0.19 | *Xylopia staudtii* | Tree | 0.56 | 0.19 | *Oncoba crepiniana* | Tree | 0.88 | 0.29 |
| 93 | *Unidentified tree 2* | Tree | 0.57 | 0.19 | *Xylopia rubescens* | Tree | 0.55 | 0.18 | Indigofera sp | Liana | 0.87 | 0.29 |
| 94 | *Cleistopholis patens* | Liana | 0.56 | 0.19 | *Trichoscypha abut* | Tree | 0.54 | 0.18 | Caloncoba sp | Tree | 0.81 | 0.27 |
| 95 | *Cola pachycarpa* | Tree | 0.54 | 0.18 | *Macaranga spinosa* | Tree | 0.52 | 0.17 | *Cissus dinklagei* | Liana | 0.79 | 0.26 |
| 96 | *Vitex grandifolia* | Tree | 0.53 | 0.18 | *Mammea africana* | Tree | 0.46 | 0.15 | *Tetrapleura tetraptera* | Tree | 0.78 | 0.26 |
| 97 | *Landolphia maxima* | Liana | 0.52 | 0.17 | *Grewia hookerana* | Liana | 0.44 | 0.15 | Combretum sp2 | Liana | 0.77 | 0.26 |
| 98 | *Amphimas pterocarpoides* | Tree | 0.52 | 0.17 | *Garcinia mannii* | Tree | 0.44 | 0.15 | *Strombosia pustulata* | Shrub | 0.75 | 0.25 |
| 99 | *Artabotrys thomsonii* | Liana | 0.51 | 0.17 | Treculia sp | Tree | 0.41 | 0.14 | *Psychotria densinervia* | Tree | 0.74 | 0.25 |
| 100 | *Trichoscypha abut* | Tree | 0.51 | 0.17 | *Desplatsia subericarpa* | Tree | 0.39 | 0.13 | *Enantia chlorantha* | Tree | 0.70 | 0.23 |
| 101 | *Piptadeniastrum africanum* | Tree | 0.5 | 0.17 | *Aulacocalyx auriculata* | Tree | 0.39 | 0.13 | *Ricinodendron heudelotii* | Tree | 0.69 | 0.23 |
| 102 | Dicranolepsis sp | Shrub | 0.5 | 0.17 | *Guarea thompsonii* | Tree | 0.39 | 0.13 | *Hugonia platysepala* | Liana | 0.65 | 0.22 |
| 103 | *Uvariopsis le-testui* | Shrub | 0.5 | 0.17 | Uapaca sp | Tree | 0.39 | 0.13 | *Pycnanthus angolensis* | Tree | 0.63 | 0.21 |
| 104 | *Chrysophyllum lacourtianum* | Tree | 0.49 | 0.16 | *Piptadeniastrum africanum* | Tree | 0.39 | 0.13 | *Uvariopsis le-testui* | Shrub | 0.61 | 0.20 |
| 105 | *Mareyopsis longifolia* | Tree | 0.48 | 0.16 | *Fagara poggei* | Tree | 0.39 | 0.13 | *Massularia acuminata* | Tree | 0.61 | 0.20 |
| 106 | *Homalium dictyoneurum* | Tree | 0.44 | 0.15 | *Alstonia boonei* | Tree | 0.34 | 0.11 | *Maesobotrya klaineana* | Tree | 0.60 | 0.20 |
| 107 | *Macaranga spinosa* | Tree | 0.43 | 0.14 | *Chytranthus talbotii* | Tree | 0.34 | 0.11 | *Markhamia tomentosa* | Tree | 0.60 | 0.20 |
| 108 | *Xylopia hypolampra* | Tree | 0.42 | 0.14 | *Chrysophyllum lacourtianum* | Tree | 0.26 | 0.09 | *Lovoa trichilioides* | Tree | 0.60 | 0.20 |
| 109 | Strychnos sp1 | Liana | 0.42 | 0.14 | *Fagara macrophylla* | Tree | 0.24 | 0.08 | *Irvingia robur* | Tree | 0.57 | 0.19 |
| 110 | Psychotria sp | NA | 0.4 | 0.13 | Millettia sp | Liana | 0.23 | 0.08 | Treculia sp | Tree | 0.56 | 0.19 |
| 111 | *Leea guineense* | Shrub | 0.4 | 0.13 | *Acacia pennata* | Liana | 0.22 | 0.07 | *Desplatsia subericarpa* | Tree | 0.55 | 0.18 |
| 112 | Strephonema sp | Tree | 0.4 | 0.13 | *Landolphia jumellei* | Liana | 0.22 | 0.07 | *Campylospermum elongatum* | Shrub | 0.55 | 0.18 |
| 113 | *Mammea africana* | Tree | 0.4 | 0.13 | *Cylicodiscus gabunensis* | Tree | 0.21 | 0.07 | *Warneckea cinnamomoides* | Tree | 0.55 | 0.18 |
| 114 | *Pachypodanthium staudtii* | Tree | 0.38 | 0.13 | *Pterocarpus soyauxii* | Tree | 0.21 | 0.07 | *Rhabdophyllum calophyllum* | Tree | 0.55 | 0.18 |
| 115 | *Grewia hookerana* | Liana | 0.38 | 0.13 | *Didymosalpinx lanciloba* | Shrub | 0.20 | 0.07 | *Fagara poggei* | Tree | 0.54 | 0.18 |
| 116 | *Rhabdophyllum calophyllum* | Tree | 0.38 | 0.13 | *Dacryodes buettneri* | Tree | 0.20 | 0.07 | *Unidentified tree 12* | Tree | 0.52 | 0.17 |
| 117 | *Entada purseatha* | Liana | 0.35 | 0.12 | *Unidentified liana 7* | Liana | 0.20 | 0.07 | *Tetracera podotricha* | Liana | 0.51 | 0.17 |
| 118 | *Dacryodes buettneri* | Tree | 0.34 | 0.11 | *Homalium dictyoneurum* | Tree | 0.19 | 0.06 | *Chytranthus talbotii* | Tree | 0.51 | 0.17 |
| 119 | *Funtumia elastica* | Tree | 0.32 | 0.11 | *Garcinia kola* | Tree | 0.19 | 0.06 | *Klainedoxa gabonensis* | Tree | 0.51 | 0.17 |
| 120 | *Landolphia jumellei* | Liana | 0.3 | 0.1 | *Theobroma cacao* | Tree | 0.19 | 0.06 | *Guarea cedrata* | Tree | 0.50 | 0.17 |
| 121 | *Pauridiantha canthiiflora* | Shrub | 0.3 | 0.1 | *Staudtia kamerunensis* | Tree | 0.19 | 0.06 | *Garcinia kola* | Tree | 0.50 | 0.17 |
| 122 | *Mucuna flagellipes* | Liana | 0.28 | 0.09 | *Voacanga africana* | Tree | 0.19 | 0.06 | *Baissea mortehanii* | Liana | 0.45 | 0.15 |
| 123 | *Fagara macrophylla* | Tree | 0.28 | 0.09 | *Ficus mucuso* | Tree | 0.19 | 0.06 | *Cylicodiscus gabunensis* | Tree | 0.43 | 0.14 |
| 124 | *Sterculia tragacantha* | Tree | 0.27 | 0.09 | *Uvariopsis le-testui* | Shrub | 0.19 | 0.06 | *Pseudospondias microcarpa* | Tree | 0.42 | 0.14 |
| 125 | *Baissea mortehanii* | Liana | 0.26 | 0.09 | *Antidesma laciniatum* | Tree | 0.19 | 0.06 | *Tetrorchidium didymostemon* | Tree | 0.39 | 0.13 |
| 126 | *Garcinia kola* | Tree | 0.26 | 0.09 | *Artabotrys thomsonii* | Liana | 0.19 | 0.06 | *Polyscias fulva* | Tree | 0.39 | 0.13 |
| 127 | *Irvingia gabonensis* | Tree | 0.26 | 0.09 | *Cordia platythyrsa* | Tree | 0.19 | 0.06 | *Terminalia superba* | Tree | 0.38 | 0.13 |
| 128 | *Fagara dinklagei* | Tree | 0.26 | 0.09 | *Glyphaea brevis* | Tree | 0.19 | 0.06 | *Maesopsis eminii* | Tree | 0.34 | 0.11 |
| 129 | *Clerodendron carnulosum* | Liana | 0.25 | 0.08 | *Musanga cecropioides* | Tree | 0.19 | 0.06 | *Ficus mucuso* | Tree | 0.33 | 0.11 |
| 130 | *Agelaea pseudobliqua* | Liana | 0.25 | 0.08 | Psychotria sp | NA | 0.19 | 0.06 | *Mucuna flagellipes* | Liana | 0.33 | 0.11 |
| 131 | *Agelaea paradoxa* | Liana | 0.25 | 0.08 | *Fagara claessensii* | Liana | 0.19 | 0.06 | *Friesodielsia enghiana* | Liana | 0.33 | 0.11 |
| 132 | *Unidentified liana 7* | Liana | 0.25 | 0.08 | *Oncoba crepiniana* | Tree | 0.19 | 0.06 | *Diospyros crassiflora* | Tree | 0.32 | 0.11 |
| 133 | *Xylopia staudtii* | Tree | 0.25 | 0.08 | *Rauvolfia woodsoniana* | Tree | 0.19 | 0.06 | *Millettia barteri* | Liana | 0.31 | 0.10 |
| 134 | *Omphalocarpum elatum* | Tree | 0.25 | 0.08 | *Roureopsis obliquifoliolata* | Liana | 0.19 | 0.06 | *Glyphaea brevis* | Tree | 0.31 | 0.10 |
| 135 | Macaranga sp1 | Liana | 0.25 | 0.08 | Combretum sp2 | Liana | 0.19 | 0.06 | Uapaca sp | Tree | 0.31 | 0.10 |
| 136 | Drypetes sp | Tree | 0.25 | 0.08 | / | / | / | / | *Landolphia jumellei* | Liana | 0.30 | 0.10 |
| 137 | *Strychnos aculeata* | Liana | 0.25 | 0.08 | / | / | / | / | *Artabotrys thomsonii* | Liana | 0.29 | 0.10 |
| 138 | *Lepidobotrys staudtii* | Tree | 0.24 | 0.08 | / | / | / | / | *Landolphia maxima* | Liana | 0.29 | 0.10 |
| 139 | *Glyphaea brevis* | Tree | 0.2 | 0.07 | / | / | / | / | *Desplatsia dewevrei* | Tree | 0.28 | 0.09 |
| 140 | *Neoboutonia glabuscens* | Tree | 0.19 | 0.06 | / | / | / | / | *Mammea africana* | Tree | 0.28 | 0.09 |
| 141 | *Chytranthus talbotii* | Tree | 0.18 | 0.06 | / | / | / | / | *Syzygium guineense* | Tree | 0.28 | 0.09 |
| 142 | *Garcinia punctata* | Tree | 0.17 | 0.06 | / | / | / | / | *Aidia micrantha* | Tree | 0.28 | 0.09 |
| 143 | *Lovoa trichilioides* | Tree | 0.17 | 0.06 | / | / | / | / | *Irvingia gabonensis* | Tree | 0.28 | 0.09 |
| 144 | *Antrocaryon klaineanum* | Tree | 0.16 | 0.05 | / | / | / | / | *Clerodendron carnulosum* | Liana | 0.28 | 0.09 |
| 145 | *Drypetes gossweileri* | Tree | 0.15 | 0.05 | / | / | / | / | *Smilax kraussiana* | Liana | 0.28 | 0.09 |
| 146 | *Landolphia landolphioides* | Liana | 0.15 | 0.05 | / | / | / | / | *Xylopia staudtii* | Tree | 0.27 | 0.09 |
| 147 | *Millettia barteri* | Liana | 0.14 | 0.05 | / | / | / | / | *Vitex grandifolia* | Tree | 0.26 | 0.09 |
| 148 | *Trichoscypha acuminata* | Tree | 0.14 | 0.05 | / | / | / | / | *Psidium guajava* | Tree | 0.26 | 0.09 |
| 149 | *Lasiodiscus marmoratus* | Tree | 0.14 | 0.05 | / | / | / | / | *Cola caricaefolia* | Tree | 0.25 | 0.08 |
| 150 | *Pseudospondias microcarpa* | Tree | 0.14 | 0.05 | / | / | / | / | *Croton longiracemosus* | Tree | 0.25 | 0.08 |
| 151 | Caloncoba sp | Tree | 0.14 | 0.05 | / | / | / | / | Millettia sp | Liana | 0.23 | 0.08 |
| 152 | *Tetrorchidium didymostemon* | Tree | 0.13 | 0.04 | / | / | / | / | *Fagara macrophylla* | Tree | 0.23 | 0.08 |
| 153 | *Tabernaemontana penduliflora* | Tree | 0.13 | 0.04 | / | / | / | / | *Bridelia grandis* | Tree | 0.20 | 0.07 |
| 154 | *Vitex myrmecophila* | Liana | 0.13 | 0.04 | / | / | / | / | *Unidentified tree 7* | Tree | 0.20 | 0.07 |
| 155 | *Albizia adianthifolia* | Tree | 0.13 | 0.04 | / | / | / | / | *Pteleopsis hylodendron* | Tree | 0.19 | 0.06 |
| 156 | *Ancistrophyllum secondiflorum* | Palm | 0.13 | 0.04 | / | / | / | / | *Manilkara zenkeri* | Tree | 0.16 | 0.05 |
| 157 | *Vernonia frondosa* | Tree | 0.13 | 0.04 | / | / | / | / | *Cola arcuata* | Tree | 0.16 | 0.05 |
| 158 | Landolphia sp2 | Liana | 0.13 | 0.04 | / | / | / | / | *Cordia platythyrsa* | Tree | 0.16 | 0.05 |
| 159 | *Markhamia tomentosa* | Tree | 0.13 | 0.04 | / | / | / | / | *Bridelia micrantha* | Tree | 0.15 | 0.05 |
| 160 | Combretum sp1 | Liana | 0.13 | 0.04 | / | / | / | / | *Baillonella toxisperma* | Tree | 0.15 | 0.05 |
| 161 | *Landolphia glabra* | Liana | 0.12 | 0.04 | / | / | / | / | *Pauridiantha canthiiflora* | Shrub | 0.15 | 0.05 |
| 162 | *Voacanga africana* | Tree | 0.12 | 0.04 | / | / | / | / | *Strelitzia madagascariensis* | Palm | 0.15 | 0.05 |
| 163 | *Acacia pennata* | Liana | 0.12 | 0.04 | / | / | / | / | Rinorea sp | Tree | 0.15 | 0.05 |
| 164 | *Aidia micrantha* | Tree | 0.12 | 0.04 | / | / | / | / | Ficus sp | Liana | 0.14 | 0.05 |
| 165 | *Picralima nitida* | Tree | 0.12 | 0.04 | / | / | / | / | *Lavigeria macrocarpa* | Liana | 0.14 | 0.05 |
| 166 | *Didymosalpinx lanciloba* | Shrub | 0.12 | 0.04 | / | / | / | / | *Ancistrophyllum secondiflorum* | Palm | 0.14 | 0.05 |
| 167 | *Friesodielsia enghiana* | Liana | 0.12 | 0.04 | / | / | / | / | *Roureopsis obliquifoliolata* | Liana | 0.14 | 0.05 |
| 168 | *Centroplacus glaucinus* | Tree | 0.12 | 0.04 | / | / | / | / | *Rauvolfia woodsoniana* | Tree | 0.14 | 0.05 |
| 169 | / | / | / | / | / | / | / | / | *Unidentified tree 1* | Tree | 0.14 | 0.05 |
| 170 | / | / | / | / | / | / | / | / | *Landolphia glabra* | Liana | 0.14 | 0.05 |
| 171 | / | / | / | / | / | / | / | / | *Trichoscypha abut* | Tree | 0.14 | 0.05 |
| 172 | / | / | / | / | / | / | / | / | *Homalium dictyoneurum* | Tree | 0.14 | 0.05 |
| 173 | / | / | / | / | / | / | / | / | *Unidentified liana 10* | Liana | 0.14 | 0.05 |
| 174 | / | / | / | / | / | / | / | / | *Fagara claessensii* | Liana | 0.14 | 0.05 |
| 175 | / | / | / | / | / | / | / | / | Drypetes sp | Tree | 0.14 | 0.05 |
| 176 | / | / | / | / | / | / | / | / | *Pachypodanthium staudtii* | Tree | 0.14 | 0.05 |
| 177 | / | / | / | / | / | / | / | / | *Didymosalpinx lanciloba* | Shrub | 0.14 | 0.05 |
| 178 | / | / | / | / | / | / | / | / | *Vernonia frondosa* | Tree | 0.14 | 0.05 |
| 179 | / | / | / | / | / | / | / | / | *Unidentified liana 9* | Liana | 0.14 | 0.05 |
| 180 | / | / | / | / | / | / | / | / | *Acacia pennata* | Liana | 0.14 | 0.05 |
| 181 | / | / | / | / | / | / | / | / | *Unidentified liana 3* | Liana | 0.14 | 0.05 |
| 182 | / | / | / | / | / | / | / | / | *Vitex myrmecophila* | Liana | 0.14 | 0.05 |
| 183 | / | / | / | / | / | / | / | / | Dicranolepsis sp | Shrub | 0.14 | 0.05 |
| 184 | / | / | / | / | / | / | / | / | Erythrococca sp | Tree | 0.14 | 0.05 |

The Importance value (IV), the percentage of the total IV (% IV), and the morphospecies are shown for each species. The colours of the 20 highest ranking species show which species have high importance values at all three sites (green), at two sites (yellow) or only at a single site (red).

When only considering the largest stems (trees ≥10cm and lianas ≥5cm in diameter), the importance values of the most important species followed a similar pattern at all sites (Table 2). There was one species at each site with a markedly higher importance value than the other species. The lower ranking plants showed more gradual differences. Only three species (*Petersianthus macrocarpus*, *Uapaca paludosa*, and *Neuropeltis acuminata*) were part of the twenty highest ranking plants at all sites. In La Belgique and Ngouleminanga the most important species was the tree *Petersianthus macrocarpus* (LB, IV = 23.48; NG, IV = 21.81). The most important species in Palestine was the tree *Heisteria parvifolia* (IV = 21.24).

All five focal tree species had relatively low importance values and not all species were recorded in the large plots at the three study sites (Table 1). *D. macrocarpa* had the highest importance value (IV = 2.41) in La Belgique. In Palestine the highest-ranking focal tree was *T. tetraptera* (IV = 2.40), while *A. klaineanum* was the most important focal tree (IV = 3.08) in Ngouleminanga. The ranking order of the five focal tree species was similar when only the largest stems were considered (Table 2)

**Table 2: The importance values for the woody species in the assemblage containing only the largest stems (trees ≥10cm diameter and lianas ≥5cm diameter).**

|  | La Belgique | | | | Palestine | | | | Ngouleminanga | | | |
| --- | --- | --- | --- | --- | --- | --- | --- | --- | --- | --- | --- | --- |
| Nr. | Species | Morphospecies | IV | % IV | Species | Morphospecies | IV | % IV | Species | Morphospecies | IV | % IV |
| 1 | *Petersianthus macrocarpus* | Tree | 23.48 | 7.83 | *Heisteria parvifolia* | Tree | 21.24 | 7.08 | *Petersianthus macrocarpus* | Tree | 21.81 | 7.27 |
| 2 | *Tabernaemontana crassa* | Tree | 16.87 | 5.62 | *Petersianthus macrocarpus* | Tree | 17.14 | 5.71 | *Heisteria parvifolia* | Tree | 15.63 | 5.21 |
| 3 | *Uapaca guineensis* | Tree | 15.57 | 5.19 | *Uapaca paludosa* | Tree | 17.10 | 5.70 | *Uapaca paludosa* | Tree | 13.28 | 4.43 |
| 4 | *Raphia monbuttorum* | Palm | 13.85 | 4.62 | *Uapaca acuminata* | Tree | 16.35 | 5.45 | *Pentaclethra macrophylla* | Tree | 12.56 | 4.19 |
| 5 | *Neuropeltis acuminata* | Liana | 13.42 | 4.47 | *Distemonanthus benthamianus* | Tree | 14.13 | 4.71 | *Distemonanthus benthamianus* | Tree | 9.71 | 3.24 |
| 6 | *Desbordesia glaucescens* | Tree | 11.54 | 3.85 | *Neuropeltis acuminata* | Liana | 11.01 | 3.67 | *Coelocaryon preussii* | Tree | 8.21 | 2.74 |
| 7 | *Anonidium mannii* | Tree | 8.96 | 2.99 | *Carapa procera* | Tree | 10.34 | 3.45 | *Musanga cecropioides* | Tree | 6.99 | 2.33 |
| 8 | *Uapaca paludosa* | Tree | 8.74 | 2.91 | *Trichilia heudelotii* | Tree | 10.16 | 3.39 | *Symphonia globulifera* | Tree | 6.79 | 2.26 |
| 9 | *Strombosia grandifolia* | Tree | 8.63 | 2.88 | *Santiria trimera* | Tree | 10.08 | 3.36 | *Hylodendron gabunense* | Tree | 6.56 | 2.19 |
| 10 | *Tetracera podotricha* | Liana | 8.12 | 2.71 | *Plagiostyles africana* | Tree | 9.75 | 3.25 | *Erythrophleum suaveolens* | Tree | 6.04 | 2.01 |
| 11 | *Plagiostyles africana* | Tree | 8.10 | 2.70 | *Tabernaemontana crassa* | Tree | 8.87 | 2.96 | *Polyalthia suaveolens* | Tree | 5.77 | 1.92 |
| 12 | *Polyalthia suaveolens* | Tree | 7.41 | 2.47 | *Hylodendron gabunense* | Tree | 8.53 | 2.84 | *Strombosia grandifolia* | Tree | 5.67 | 1.89 |
| 13 | Trichilia sp. | Tree | 7.09 | 2.36 | *Pycnanthus angolensis* | Tree | 7.90 | 2.63 | *Raphia monbuttorum* | Palm | 5.40 | 1.80 |
| 14 | *Manniophyton fulvum* | Liana | 5.86 | 1.95 | *Anonidium mannii* | Tree | 7.61 | 2.54 | *Uapaca vanhoutei* | Tree | 5.22 | 1.74 |
| 15 | *Uapaca acuminata* | Tree | 5.46 | 1.82 | *Canarium wenzelii* | Tree | 7.45 | 2.48 | *Celtis mildbraedii* | Tree | 4.97 | 1.66 |
| 16 | *Trichilia heudelotii* | Tree | 5.45 | 1.82 | *Pentaclethra macrophylla* | Tree | 6.78 | 2.26 | *Ongokea gore* | Tree | 4.82 | 1.61 |
| 17 | *Strychnos angolensis* | Liana | 4.82 | 1.61 | *Strychnos angolensis* | Liana | 6.46 | 2.15 | *Neuropeltis acuminata* | Liana | 4.79 | 1.60 |
| 18 | *Santiria trimera* | Tree | 4.82 | 1.61 | *Coelocaryon preussii* | Tree | 6.40 | 2.13 | *Antrocaryon klaineanum* | Tree | 4.69 | 1.56 |
| 19 | *Eribroma oblongum* | Tree | 4.33 | 1.44 | *Uapaca vanhoutei* | Tree | 6.04 | 2.01 | *Unidentified tree 10* | Tree | 4.68 | 1.56 |
| 20 | *Duboscia macrocarpa* | Tree | 4.20 | 1.40 | *Unidentified tree 10* | Tree | 6.03 | 2.01 | *Staudtia kamerunensis* | Tree | 4.57 | 1.52 |
| 21 | *Pterocarpus mildbraedii* | Tree | 3.99 | 1.33 | *Symphonia globulifera* | Tree | 5.31 | 1.77 | *Panda oleosa* | Tree | 4.25 | 1.42 |
| 22 | *Pausinystalia lane-poolei* | Tree | 3.97 | 1.32 | *Tetrapleura tetraptera* | Tree | 4.76 | 1.59 | *Pterocarpus soyauxii* | Tree | 4.05 | 1.35 |
| 23 | *Milicia excelsa* | Tree | 3.77 | 1.26 | *Celtis tessmannii* | Tree | 4.75 | 1.58 | *Funtumia elastica* | Tree | 3.82 | 1.27 |
| 24 | *Heisteria parvifolia* | Tree | 3.58 | 1.19 | Indigofera sp | Liana | 4.27 | 1.42 | *Pterocarpus mildbraedii* | Tree | 3.79 | 1.26 |
| 25 | *Cola balayii* | Tree | 3.25 | 1.08 | *Uapaca guineensis* | Tree | 4.03 | 1.34 | *Xylopia rubescens* | Tree | 3.53 | 1.18 |
| 26 | *Terminalia superba* | Tree | 3.16 | 1.05 | *Guarea cedrata* | Tree | 3.74 | 1.25 | Trichilia sp | Tree | 3.51 | 1.17 |
| 27 | *Enantia chlorantha* | Tree | 3.08 | 1.03 | *Klainedoxa gabonensis* | Tree | 3.21 | 1.07 | *Xylopia hypolampra* | Tree | 3.43 | 1.14 |
| 28 | *Erythrophleum suaveolens* | Tree | 3.05 | 1.02 | *Hugonia platysepala* | Liana | 3.20 | 1.07 | *Unidentified tree 11* | Tree | 3.41 | 1.14 |
| 29 | *Pentaclethra macrophylla* | Tree | 3.01 | 1.00 | *Cleistopholis patens* | Liana | 3.04 | 1.01 | *Carapa procera* | Tree | 3.38 | 1.13 |
| 30 | Combretum sp2 | Liana | 2.88 | 0.96 | Trichilia sp | Tree | 3.02 | 1.01 | Indigofera sp | Liana | 3.35 | 1.12 |
| 31 | *Chytranthus atroviolaceus* | Tree | 2.83 | 0.94 | *Landolphia violacea* | Liana | 2.94 | 0.98 | *Santiria trimera* | Tree | 3.33 | 1.11 |
| 32 | *Cordia platythyrsa* | Tree | 2.82 | 0.94 | *Tetracera podotricha* | Liana | 2.90 | 0.97 | *Duboscia macrocarpa* | Tree | 3.33 | 1.11 |
| 33 | *Sorindeia grandifolia* | Tree | 2.79 | 0.93 | *Strombosia grandifolia* | Tree | 2.84 | 0.95 | *Celtis tessmannii* | Tree | 3.30 | 1.10 |
| 34 | *Albizia zygia* | Tree | 2.75 | 0.92 | Draceana sp | Tree | 2.53 | 0.84 | *Sterculia tragacantha* | Tree | 3.21 | 1.07 |
| 35 | *Tetrapleura tetraptera* | Tree | 2.62 | 0.87 | *Antrocaryon klaineanum* | Tree | 2.41 | 0.80 | *Nauclea pobeguinii* | Tree | 3.21 | 1.07 |
| 36 | *Phyllanthus discoideus* | Tree | 2.42 | 0.81 | *Enantia chlorantha* | Tree | 2.18 | 0.73 | *Tabernaemontana crassa* | Tree | 2.84 | 0.95 |
| 37 | *Pterocarpus soyauxii* | Tree | 2.38 | 0.79 | *Lasiodiscus marmoratus* | Tree | 2.18 | 0.73 | *Uapaca acuminata* | Tree | 2.75 | 0.92 |
| 38 | *Lecaniodiscus cupanioides* | Tree | 2.37 | 0.79 | *Desbordesia glaucescens* | Tree | 1.99 | 0.66 | *Strombosiopsis tetrandra* | Tree | 2.56 | 0.85 |
| 39 | Indigofera sp | Liana | 2.19 | 0.73 | *Picralima nitida* | Tree | 1.87 | 0.62 | Dichapetalum sp | Liana | 2.53 | 0.84 |
| 40 | *Anthonotha macrophylla* | Tree | 2.12 | 0.71 | *Xylopia rubescens* | Tree | 1.78 | 0.59 | *Trichoscypha acuminata* | Tree | 2.50 | 0.83 |
| 41 | *Macaranga barteri* | Tree | 2.12 | 0.71 | *Duboscia macrocarpa* | Tree | 1.72 | 0.57 | *Dacryodes buettneri* | Tree | 2.45 | 0.82 |
| 42 | *Coelocaryon preussii* | Tree | 2.07 | 0.69 | *Polyalthia suaveolens* | Tree | 1.71 | 0.57 | *Klainedoxa microphylla* | Tree | 2.29 | 0.76 |
| 43 | *Strombosiopsis tetrandra* | Tree | 1.98 | 0.66 | *Strombosiopsis tetrandra* | Tree | 1.68 | 0.56 | *Uapaca guineensis* | Tree | 2.16 | 0.72 |
| 44 | *Hylodendron gabunense* | Tree | 1.89 | 0.63 | *Celtis mildbraedii* | Tree | 1.64 | 0.55 | *Albizia zygia* | Tree | 2.12 | 0.71 |
| 45 | *Pteleopsis hylodendron* | Tree | 1.89 | 0.63 | *Trichoscypha acuminata* | Tree | 1.64 | 0.55 | *Plagiostyles africana* | Tree | 2.12 | 0.71 |
| 46 | *Bridelia micrantha* | Tree | 1.89 | 0.63 | *Xylopia staudtii* | Tree | 1.58 | 0.53 | *Unidentified tree 12* | Tree | 2.11 | 0.70 |
| 47 | *Trichilia gilgiana* | Tree | 1.63 | 0.54 | *Alstonia boonei* | Tree | 1.55 | 0.52 | *Albizia coriaria* | Tree | 2.11 | 0.70 |
| 48 | *Celtis tessmannii* | Tree | 1.55 | 0.52 | *Chytranthus talbotii* | Tree | 1.55 | 0.52 | *Desbordesia glaucescens* | Tree | 2.08 | 0.69 |
| 49 | *Entada purseatha* | Liana | 1.53 | 0.51 | *Cola caricaefolia* | Tree | 1.54 | 0.51 | *Trichilia heudelotii* | Tree | 2.07 | 0.69 |
| 50 | *Sindoropsis le-testui* | Tree | 1.49 | 0.50 | *Lecaniodiscus cupanioides* | Tree | 1.51 | 0.50 | *Rothmannia coriacea* | Tree | 2.07 | 0.69 |
| 51 | *Landolphia jumellei* | Liana | 1.48 | 0.49 | *Chytranthus atroviolaceus* | Tree | 1.50 | 0.50 | *Entandrophragma candollei* | Tree | 2.05 | 0.68 |
| 52 | Millettia sp | Liana | 1.46 | 0.49 | *Trichoscypha abut* | Tree | 1.50 | 0.50 | *Anonidium mannii* | Tree | 1.99 | 0.66 |
| 53 | *Uapaca vanhoutei* | Tree | 1.37 | 0.46 | *Grewia hookerana* | Liana | 1.45 | 0.48 | *Diospyros holeana* | Tree | 1.99 | 0.66 |
| 54 | *Unidentified tree 2* | Tree | 1.20 | 0.40 | *Manniophyton fulvum* | Liana | 1.45 | 0.48 | *Piptadeniastrum africanum* | Tree | 1.97 | 0.66 |
| 55 | *Psychotria densinervia* | Tree | 1.11 | 0.37 | Millettia sp | Liana | 1.43 | 0.48 | *Alstonia boonei* | Tree | 1.94 | 0.65 |
| 56 | *Strombosia pustulata* | Shrub | 1.11 | 0.37 | *Acacia pennata* | Liana | 1.43 | 0.48 | *Macaranga barteri* | Tree | 1.90 | 0.63 |
| 57 | *Mareyopsis longifolia* | Tree | 1.10 | 0.37 | *Cissus dinklagei* | Liana | 1.42 | 0.47 | *Irvingia grandifolia* | Tree | 1.80 | 0.60 |
| 58 | *Celtis mildbraedii* | Tree | 1.08 | 0.36 | *Landolphia jumellei* | Liana | 1.42 | 0.47 | *Fagara dinklagei* | Tree | 1.78 | 0.59 |
| 59 | *Macaranga spinosa* | Tree | 1.05 | 0.35 | / | / | / | / | *Tetrapleura tetraptera* | Tree | 1.61 | 0.54 |
| 60 | *Distemonanthus benthamianus* | Tree | 1.04 | 0.35 | / | / | / | / | *Ricinodendron heudelotii* | Tree | 1.51 | 0.50 |
| 61 | *Xylopia hypolampra* | Tree | 1.03 | 0.34 | / | / | / | / | *Irvingia robur* | Tree | 1.33 | 0.44 |
| 62 | *Diospyros crassiflora* | Tree | 1.03 | 0.34 | / | / | / | / | *Albizia adianthifolia* | Tree | 1.31 | 0.44 |
| 63 | *Myrianthus arboreus* | Tree | 0.93 | 0.31 | / | / | / | / | *Markhamia tomentosa* | Tree | 1.27 | 0.42 |
| 64 | *Olax latifolia* | Tree | 0.91 | 0.30 | / | / | / | / | *Desplatsia subericarpa* | Tree | 1.21 | 0.40 |
| 65 | *Cleistopholis patens* | Liana | 0.89 | 0.30 | / | / | / | / | *Terminalia superba* | Tree | 1.19 | 0.40 |
| 66 | *Vitex grandifolia* | Tree | 0.87 | 0.29 | / | / | / | / | *Macaranga spinosa* | Tree | 1.17 | 0.39 |
| 67 | *Cola arcuata* | Tree | 0.86 | 0.29 | / | / | / | / | *Gambeya boukokoensis* | Tree | 1.14 | 0.38 |
| 68 | *Desplatsia dewevrei* | Tree | 0.86 | 0.29 | / | / | / | / | *Diospyros crassiflora* | Tree | 1.12 | 0.37 |
| 69 | *Maesobotrya klaineana* | Tree | 0.86 | 0.29 | / | / | / | / | *Enantia chlorantha* | Tree | 1.08 | 0.36 |
| 70 | *Lepidobotrys staudtii* | Tree | 0.84 | 0.28 | / | / | / | / | *Lecaniodiscus cupanioides* | Tree | 1.08 | 0.36 |
| 71 | *Dacryodes buettneri* | Tree | 0.82 | 0.27 | / | / | / | / | *Lepidobotrys staudtii* | Tree | 1.07 | 0.36 |
| 72 | *Desplatsia subericarpa* | Tree | 0.81 | 0.27 | / | / | / | / | *Vitex grandifolia* | Tree | 1.06 | 0.35 |
| 73 | Maesobotrya sp | Tree | 0.80 | 0.27 | / | / | / | / | *Tetrorchidium didymostemon* | Tree | 1.03 | 0.34 |
| 74 | *Garcinia mannii* | Tree | 0.79 | 0.26 | / | / | / | / | Millettia sp | Liana | 1.03 | 0.34 |
| 75 | *Funtumia elastica* | Tree | 0.79 | 0.26 | / | / | / | / | *Fagara macrophylla* | Tree | 1.02 | 0.34 |
| 76 | *Neoboutonia glabuscens* | Tree | 0.78 | 0.26 | / | / | / | / | *Guarea cedrata* | Tree | 1.02 | 0.34 |
| 77 | *Oncoba crepiniana* | Tree | 0.77 | 0.26 | / | / | / | / | *Chytranthus talbotii* | Tree | 1.01 | 0.34 |
| 78 | *Chytranthus talbotii* | Tree | 0.77 | 0.26 | / | / | / | / | *Garcinia kola* | Tree | 1.01 | 0.34 |
| 79 | *Syzygium guineense* | Tree | 0.76 | 0.25 | / | / | / | / | *Pycnanthus angolensis* | Tree | 1.01 | 0.34 |
| 80 | *Tricalysia oligoneura* | Tree | 0.76 | 0.25 | / | / | / | / | *Bridelia grandis* | Tree | 0.99 | 0.33 |
| 81 | *Hugonia platysepala* | Liana | 0.74 | 0.25 | / | / | / | / | *Cola caricaefolia* | Tree | 0.99 | 0.33 |
| 82 | *Mucuna flagellipes* | Liana | 0.74 | 0.25 | / | / | / | / | *Unidentified tree 7* | Tree | 0.99 | 0.33 |
| 83 | *Landolphia landolphioides* | Liana | 0.74 | 0.25 | / | / | / | / | *Klainedoxa gabonensis* | Tree | 0.98 | 0.33 |
| 84 | *Millettia barteri* | Liana | 0.73 | 0.24 | / | / | / | / | *Strombosia pustulata* | Shrub | 0.98 | 0.33 |
| 85 | *Baissea mortehanii* | Liana | 0.73 | 0.24 | / | / | / | / | *Fagara poggei* | Tree | 0.98 | 0.33 |
| 86 | Dichapetalum sp | Liana | 0.72 | 0.24 | / | / | / | / | *Rinorea dentata* | Tree | 0.98 | 0.33 |
| 87 | *Landolphia violacea* | Liana | 0.72 | 0.24 | / | / | / | / | *Landolphia violacea* | Liana | 0.96 | 0.32 |
| 88 | *Artabotrys thomsonii* | Liana | 0.72 | 0.24 | / | / | / | / | *Millettia barteri* | Liana | 0.96 | 0.32 |
| 89 | *Cissus dinklagei* | Liana | 0.72 | 0.24 | / | / | / | / | *Strychnos angolensis* | Liana | 0.96 | 0.32 |
| 90 | *Grewia hookerana* | Liana | 0.72 | 0.24 | / | / | / | / | *Cissus dinklagei* | Liana | 0.95 | 0.32 |
| 91 | / | / | / | / | / | / | / | / | *Baissea mortehanii* | Liana | 0.95 | 0.32 |
| 92 | / | / | / | / | / | / | / | / | *Tetracera podotricha* | Liana | 0.95 | 0.32 |
| 93 | / | / | / | / | / | / | / | / | *Landolphia jumellei* | Liana | 0.94 | 0.31 |
| 94 | / | / | / | / | / | / | / | / | *Artabotrys thomsonii* | Liana | 0.94 | 0.31 |

The Importance value (IV), the percentage of the total IV (% IV), and the morphospecies are shown for each species. The colours of the 20 highest ranking species show which species have high importance values at all three sites (green), at two sites (yellow) or only at a single site (red).

## Species diversity

Species diversity of stems larger than one metre, were clearly higher in Ngouleminanga (H = 4.43) compared to the other sites. Palestine and La Belgique differed less, but La Belgique (H = 4.13) had a slightly higher diversity than Palestine (H = 3.92). Considering only the largest woody stems (trees ≥10cm diameter and lianas ≥5cm diameter) resulted in a similar pattern. Again, Ngouleminanga (H = 4.4) had the highest level of diversity, but the difference between La Belgique (H = 4.08) and Palestine (H = 3.98) was even smaller. The species richness, of stems larger than one metre, was slightly lower in Palestine compared to the other sites; however, the 95% confidence interval overlapped with La Belgique and at the end of the curve with Ngouleminanga (Fig 1). The same pattern emerged when only the largest stems were considered (Fig 2).

**Fig 1: Extrapolated rarefaction curves for woody stems >1m at the three study sites.** Curves were extrapolated until a plateau was reached. The 95% confidence interval of La Belgique (least defaunated) overlaps with both Palestine (intermediate) and Ngouleminanga (most defaunated). Palestine has no more overlap with Ngouleminanga after approximately 550 stems, but the 95% confidence intervals start to overlap again after 5170 stems. Differences are statistically non-significant when confidence intervals overlap.

**Fig 2: Extrapolated rarefaction curves for the largest woody stems (trees ≥10cm diameter and lianas ≥5cm diameter) at the three study sites.** Curves were extrapolated until a plateau was reached. The 95% confidence interval of La Belgique (least defaunated) overlaps with both Palestine (intermediate) and Ngouleminanga (most defaunated). Palestine has no more overlap with Ngouleminanga after approximately 450 stems, but the 95% confidence intervals start to overlap again after 1420 stems. Differences are statistically non-significant when confidence intervals overlap.

## Dispersal mode composition

A few differences in the abundance of dispersal modes were identified in the large stem assemblages (>1m) between the study sites (Table 3 & Fig 3). The abundance of wind-dispersed stems differed clearly between sites. Post-hoc pairwise comparison indicated that Ngouleminanga had a significantly higher abundance of wind-dispersed species compared to the other sites (LB-NG, P-value = <0.001; NG-PA, P-value = <0.001). Differences in the abundance of mammal- dispersed stems was significant, however post-hoc analysis showed only non-significant differences. In Ngouleminanga the abundance of mammal-dispersed stems was slightly higher compared to the other sites. When only considering the largest stems, a small difference in the abundance of water-dispersed stems was identified (Table 3 & Fig 4), with mean ranks of 67.50 in La Belgique, 73.14 in Palestine, and 78.49 in Ngouleminanga. Ngouleminanga and Palestine had higher abundances of large water-dispersed stems compared to La Belgique. The difference between La Belgique and Palestine was not significant (LB-NG, P-value = 0.0057; LB-PA, P-value = 0.058). However, this result is based on a small number of observations of water-dispersed stems.

**Table 3: Comparison of the dispersal modes of large woody stems between the study sites.**

| **Large plots: all stems >1m** | | | | | | | | |
| --- | --- | --- | --- | --- | --- | --- | --- | --- |
| Dispersal mode | La Belgique | | Palestine | | Ngouleminanga | | Test (df = 2) | |
|  | Median abund. | CI | Median abund. | CI | Median abund. | CI | χ² | P-value |
| Bird | 8 | 7; 9.5 | 7 | 5.5; 8.5 | 9 | 8; 11 | 5.88 | 0.053 |
| Mammal | 14 | 13; 17 | 14 | 12.5; 16.5 | 18.5 | 15; 20 | 7.01 | 0.030 * |
| Wind | 2 | 1; 2 | 1.5 | 1; 2.5 | 4 | 3; 5 | 18.34 | 0.0001 *** |
| Water | 0 | 0; 0 | 0 | 0; 0 | 0 | 0; 0 | 5.39 | 0.068 |
| Explosion | 1 | 1; 1 | 1 | 0; 1 | 1 | 1; 1 | 1.63 | 0.44 |
| Drop | 5.5 | 4; 6.5 | 4.5 | 3.5; 5 | 5 | 4; 6 | 5.40 | 0.067 |
| **Large plots: trees ≥10cm and lianas ≥5cm in diameter** | | | | | | | | |
| Dispersal mode | La Belgique | | Palestine | | Ngouleminanga | | Test (df = 2) | |
|  | Median abund. | CI | Median abund. | CI | Median abund. | CI | χ² | P-value |
| Bird | 1 | 1; 2 | 1 | 1; 2 | 2 | 2; 2 | 5.92 | 0.052 |
| Mammal | 3 | 2; 3 | 3 | 2; 3 | 3 | 2.5; 4 | 2.47 | 0.29 |
| Wind | 1 | 0; 1 | 0 | 0; 0.5 | 1 | 0; 1 | 4.19 | 0.12 |
| Water | 0 | 0; 0 | 0 | 0; 0 | 0 | 0; 0 | 9.38 | 0.0092 ** |
| Explosion | 0 | 0; 0 | 0 | 0; 0 | 0 | 0; 0 | 4.05 | 0.13 |
| Drop | 1 | 0; 1 | 1 | 0; 1 | 1 | 0.5; 1 | 1.72 | 0.42 |

Results for the assemblage of all woody stems >1m and the assemblage of only the largest woody stems (trees ≥10cm diameter and lianas ≥5cm diameter) are presented separately. The median abundance, 95% percentile confidence intervals (CI), significance, and test statistic (χ²) are shown. For water dispersal the shape assumption of the median test was not met. Therefore, the mean ranks were compared using the Kruskal-Wallis test. Significant differences between sites are indicated: * = P<0.05; ** = P < 0.01; *** = P < 0.001.

**
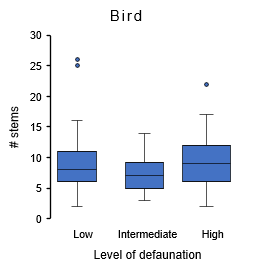

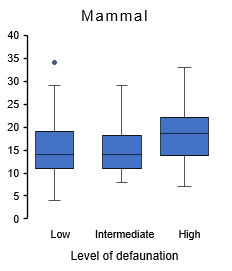

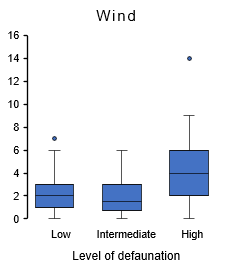

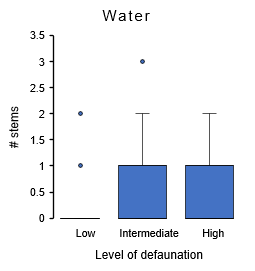

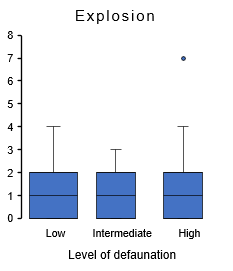

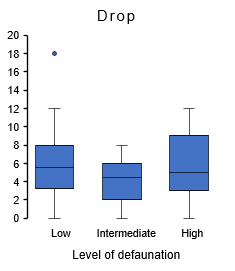
**

***

**Fig 3: Boxplots of the abundance data for woody stems of each dispersal mode in the assemblage of stems >1m at the three study sites.** Individual points represent the number of stems per plot (100m²). The three study sites: La Belgique (low), Palestine (intermediate), and Ngouleminanga (high). Significant differences between sites are indicated: * = P<0.05; ** = P < 0.01; *** = P < 0.001.


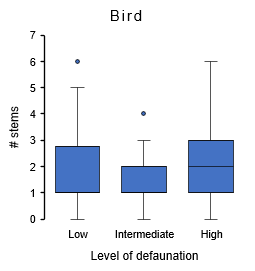

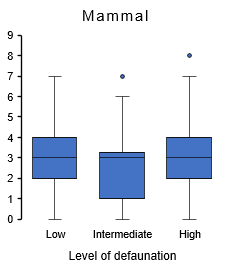

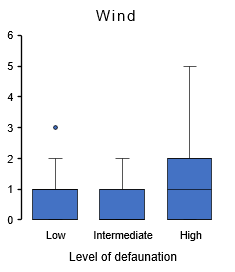

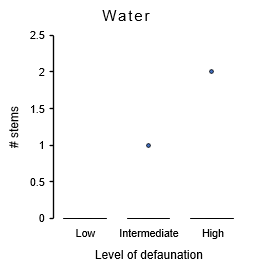

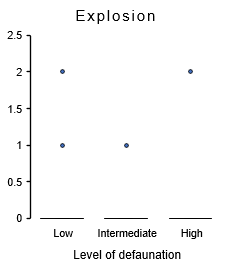

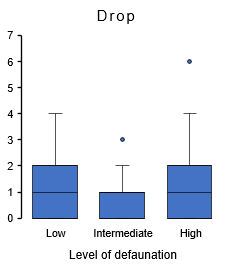


**

**Fig 4:** **Boxplots of the abundance data for woody stems of each dispersal mode in the largest stem assemblage (trees ≥10cm and lianas ≥5cm in diameter) at the three study sites.** Individual points represent the number of stems per plot (100m²). The three study sites: La Belgique (low), Palestine (intermediate), and Ngouleminanga (high). Significant differences between sites are indicated: * = P<0.05; ** = P < 0.01; *** = P < 0.001.
