# Supplementary material for: Defaunation is known to have pervasive, negative effects on tropical forests, but this is not the whole story
Source: PLoS One. 2023 Aug 31;18(8):e0290717. doi: 10.1371/journal.pone.0290717 (PMC10470957; doi:10.1371/journal.pone.0290717)
Supplement: S3 File — (DOCX) [file pone.0290717.s004.docx]

# Supplementary information 6: Conspecific density and demographics near focal trees

# Species specific differences

*K. gabonensis* differed in the number of Class 1 stems >10m away and Class 2 stems <10m away from focal trees between the study sites. Post-hoc pairwise comparisons indicated that Ngouleminanga (most defaunated) had more Class 1 stems far from the focal trees compared to La Belgique (least defaunated) (P-value = 0.012; Fig 1A). The number of Class 2 stems <10m away from focal trees was higher in La Belgique compared to Ngouleminanga (P-value = 0.024). A similar pattern was found for juveniles >10m away, but this difference was not significant (P-value = 0.057; Fig 1B). In Ngouleminanga no juvenile stems were recorded.
Seedlings were proportionally more abundant near the focal trees (60%) in La Belgique. In both Palestine (intermediate) and Ngouleminanga the percentage of Class 1 stems >10m away from focal trees (PA: 77.87%; NG: 70.91%) was higher than the percentage <10m away. In Palestine this pattern was mainly the result of strong variation in the number of stems >10m away between individual focal trees. Juveniles were observed in slightly higher proportions <10m away from focal trees in La Belgique and Palestine (LB: 59.26%; PA: 57.14%), although in La Belgique this was partly caused by an outlier.


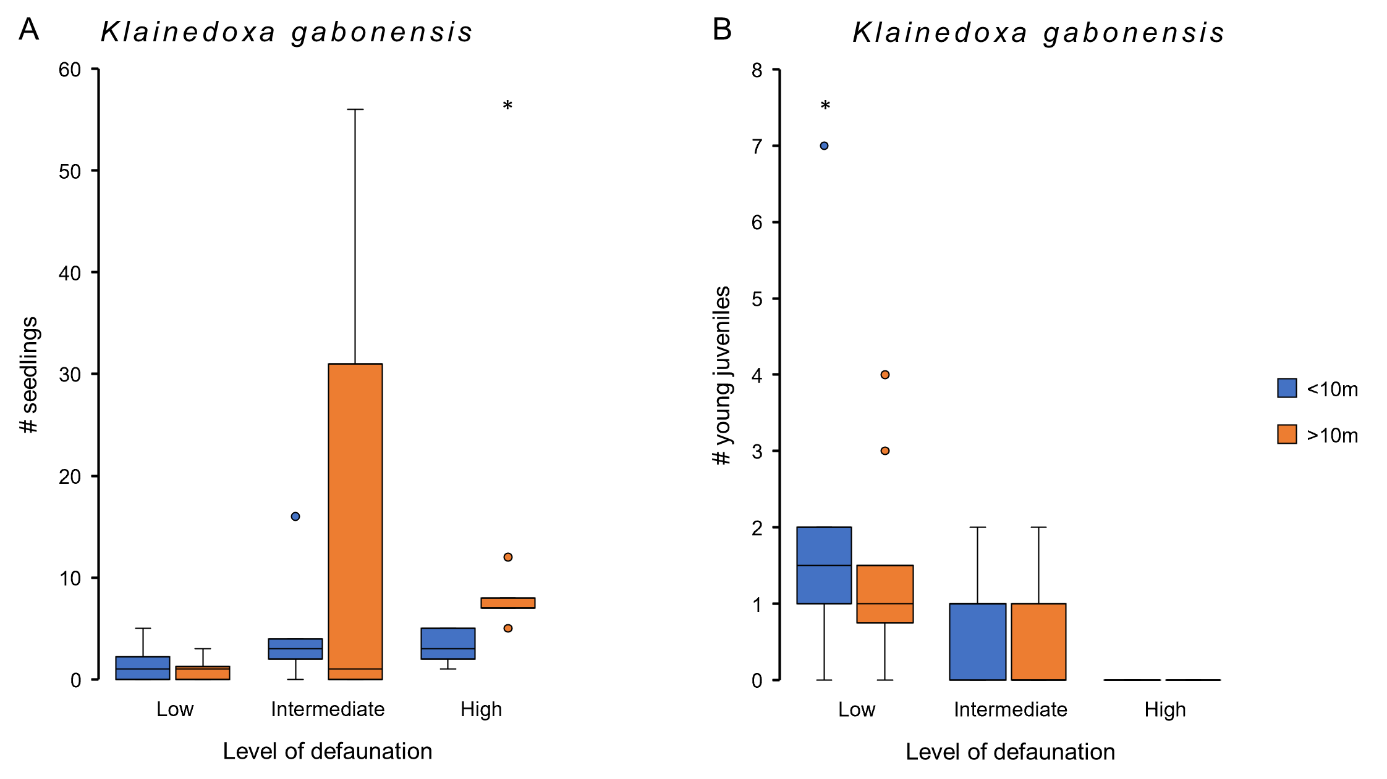


**Fig 1: Boxplots showing the distribution of (A) seedlings (Class1) and (B) juvenile stems (Class 2) in wedges under *K. gabonensis* focal trees at the three study sites.**
Stems <10m away (Near) from focal trees are in blue and stems 10–20m away (Far) from focal trees are in orange. The three study sites: La Belgique (low), Palestine (intermediate), and Ngouleminanga (high). Significant differences between sites are indicated: * = P<0.05; ** = P < 0.01; *** = P < 0.001.

*T. tetraptera* showed significant differences in the number of Class 1 stems near focal trees (Fig 2A) and a non- significant difference in the number of Class 2 stems far from focal trees (Fig 2B) between sites. A post-hoc pairwise comparison showed that both La Belgique and Palestine had a higher number of Class 1 stems near focal trees compared to Ngouleminanga (LB-NG, P-value = 0.0062; PA-NG, P-value = 0.0078).
While the percentage of stems <10m and >10m away from focal trees were similar in La Belgique and Palestine, there was a lower percentage of both seedlings and juveniles near focal trees in Ngouleminanga (Class 1: 18.60%; Class 2: 10.71%). In Palestine the percentage of juveniles was also higher far from the focal trees (69.23%) than near the trees.


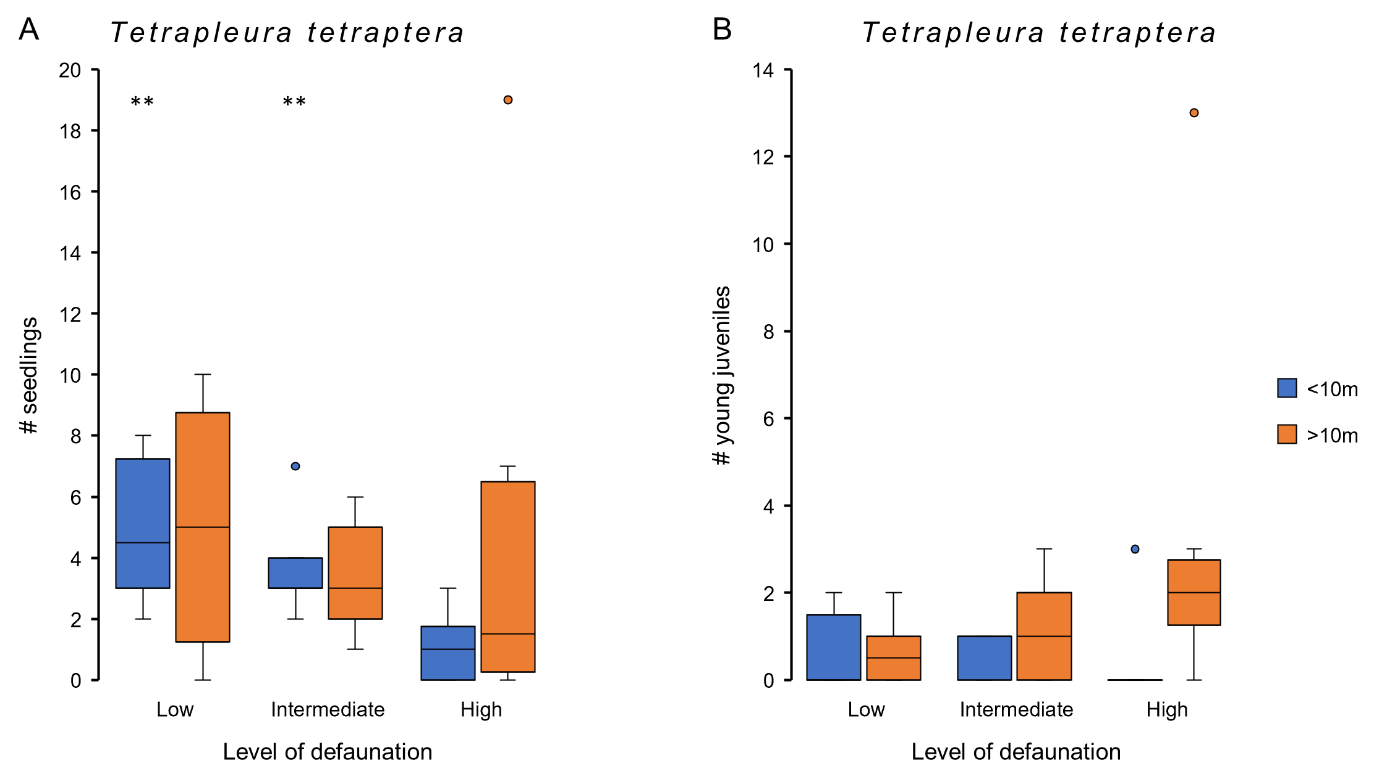


**Fig 2: Boxplots showing the distribution of (A) seedlings (Class1) and (B) juvenile stems (Class 2) in wedges under *T. tetraptera* focal trees at the three study sites.**
Stems <10m away (Near) from focal trees are in blue and stems 10–20m away (Far) from focal trees are in orange. The three study sites: La Belgique (low), Palestine (intermediate), and Ngouleminanga (high). Significant differences between sites are indicated: * = P<0.05; ** = P < 0.01; *** = P < 0.001.

*C. lacourtianum* trees differed in the number of Class 1 stems >10m away from focal trees. The sample size for Palestine was very small (n=1) and this site was therefore not included in the comparison. The sample size in Ngouleminanga was also low (n=3) and therefore these findings should be interpreted with caution. Class 1 stems were more abundant far from focal trees in Ngouleminanga compared to La Belgique (Fig 3A). Class 2 stems near focal trees were slightly more abundant in La Belgique compared to Ngouleminanga however this difference was not significant (Fig 3B).
While the percentage of seedlings and juveniles near and far from focal trees were almost equal in La Belgique, in Ngouleminanga a much higher percentage of seedlings occurred >10m away (82.76%). Juveniles, on the other hand, were recorded in a higher proportion near (66.67%) focal trees than far from trees in Ngouleminanga. However, this percentage was based on a total of only three juvenile stems.


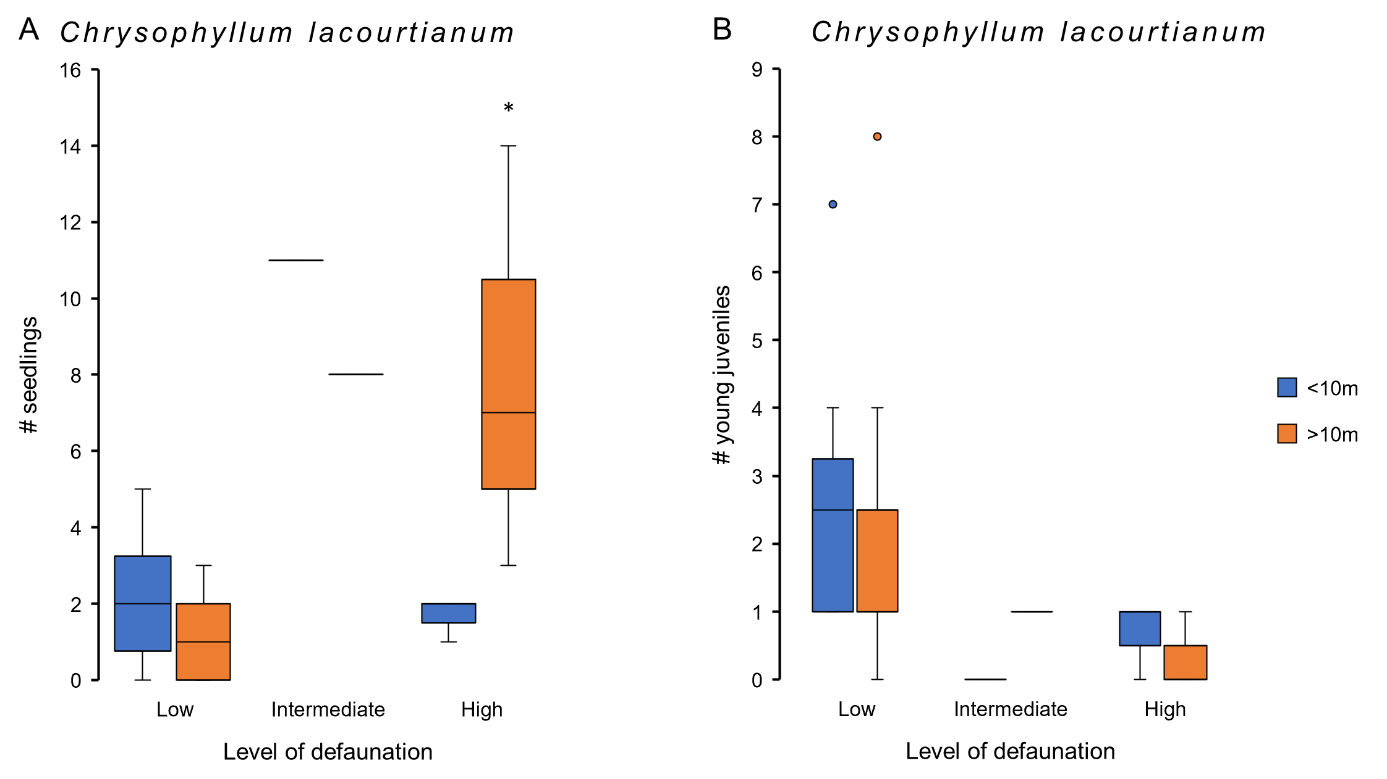


**Fig 3: Boxplots showing the distribution of (A) seedlings (Class1) and (B) juvenile stems (Class 2) in wedges under *C. lacourtianum* focal trees at the three study sites.**
Stems <10m away (Near) from focal trees are in blue and stems 10–20m away (Far) from focal trees are in orange. The three study sites: La Belgique (low), Palestine (intermediate), and Ngouleminanga (high). Significant differences between sites are indicated: * = P<0.05; ** = P < 0.01; *** = P < 0.001.

*D. macrocarpa* showed a significant difference in the number of Class 1 stems <10m away from focal trees, with a higher number in La Belgique compared to Ngouleminanga (Fig 4). Class 2 stems were almost completely absent in the wedges at all sites. In La Belgique only one stem was recorded near a focal tree, while the other sites had no Class 2 stems of this species at all. The sampling in Palestine was very limited for this species (n = 1). Statistical comparisons with this site were therefore not possible.
The percentage of seedlings near (59.27%) focal trees in La Belgique was slightly higher than the percentage far from trees, mostly because of an outlier. In Ngouleminanga the opposite pattern was observed, with a lower percentage of stems <10m away from focal trees (31.88%).


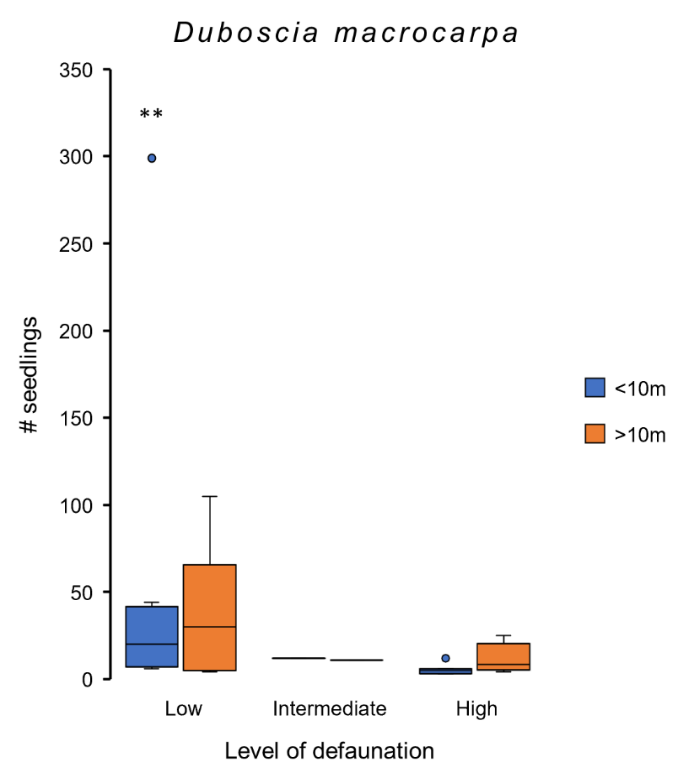


**Fig 4: Boxplots showing the distribution of seedlings (Class1) in wedges under *D. macrocarpa* focal trees at the three study sites.**
Stems <10m away (Near) from focal trees are in blue and stems 10–20m away (Far) from focal trees are in orange. The three study sites: La Belgique (low), Palestine (intermediate), and Ngouleminanga (high). Significant differences between sites are indicated: * = P<0.05; ** = P < 0.01; *** = P < 0.001.

*A. klaineanum* did not show any differences in the number of seedlings and juveniles near and far from focal trees between the three sites (Fig 5A-B). Both seedlings and juveniles showed much variation in the number of stems at several sites.
The percentage of seedlings was similar near and far from the focal trees in La Belgique and in Palestine. In Ngouleminanga a slightly higher percentage (64.71%) of seedlings was recorded far from the trees. On the other hand, juveniles were observed at lower percentage near (LB: 26.00%; PA: 20.00%; NG: 27.40%) than far from focal trees at all sites. However, this pattern was partly caused by outliers.


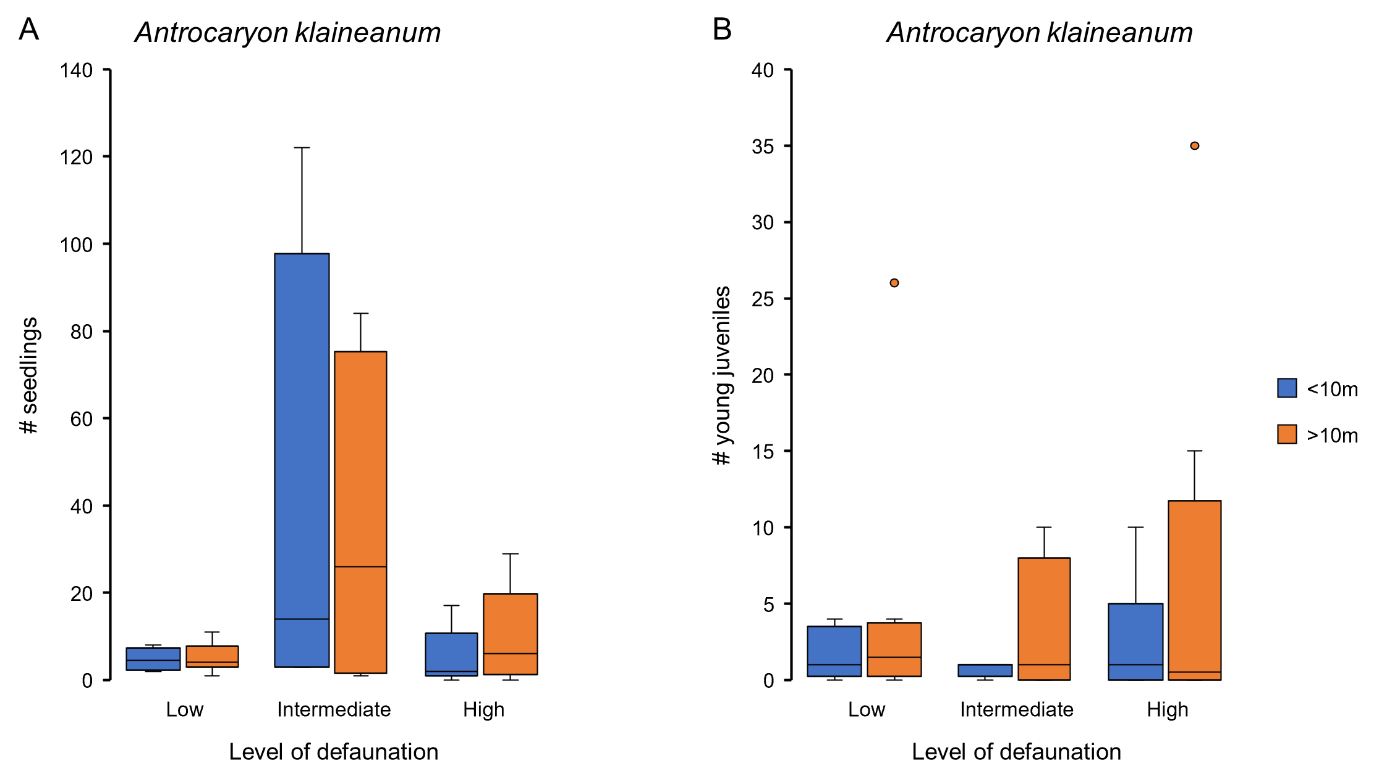


**Fig 5: Boxplots showing the distribution of (A) seedlings (Class1) and (B) juvenile stems (Class 2) in wedges under *A. klaineanum* focal trees at the three study sites.**
Stems <10m away (Near) from focal trees are in blue and stems 10–20m away (Far) from focal trees are in orange. The three study sites: La Belgique (low), Palestine (intermediate), and Ngouleminanga (high). Significant differences between sites are indicated: * = P<0.05; ** = P < 0.01; *** = P < 0.001.
