# Supplementary material for: Defaunation is known to have pervasive, negative effects on tropical forests, but this is not the whole story
Source: PLoS One. 2023 Aug 31;18(8):e0290717. doi: 10.1371/journal.pone.0290717 (PMC10470957; doi:10.1371/journal.pone.0290717)
Supplement: S1 Fig — (DOCX) [file pone.0290717.s005.docx]

# Supplementary information 5: Additional figures.


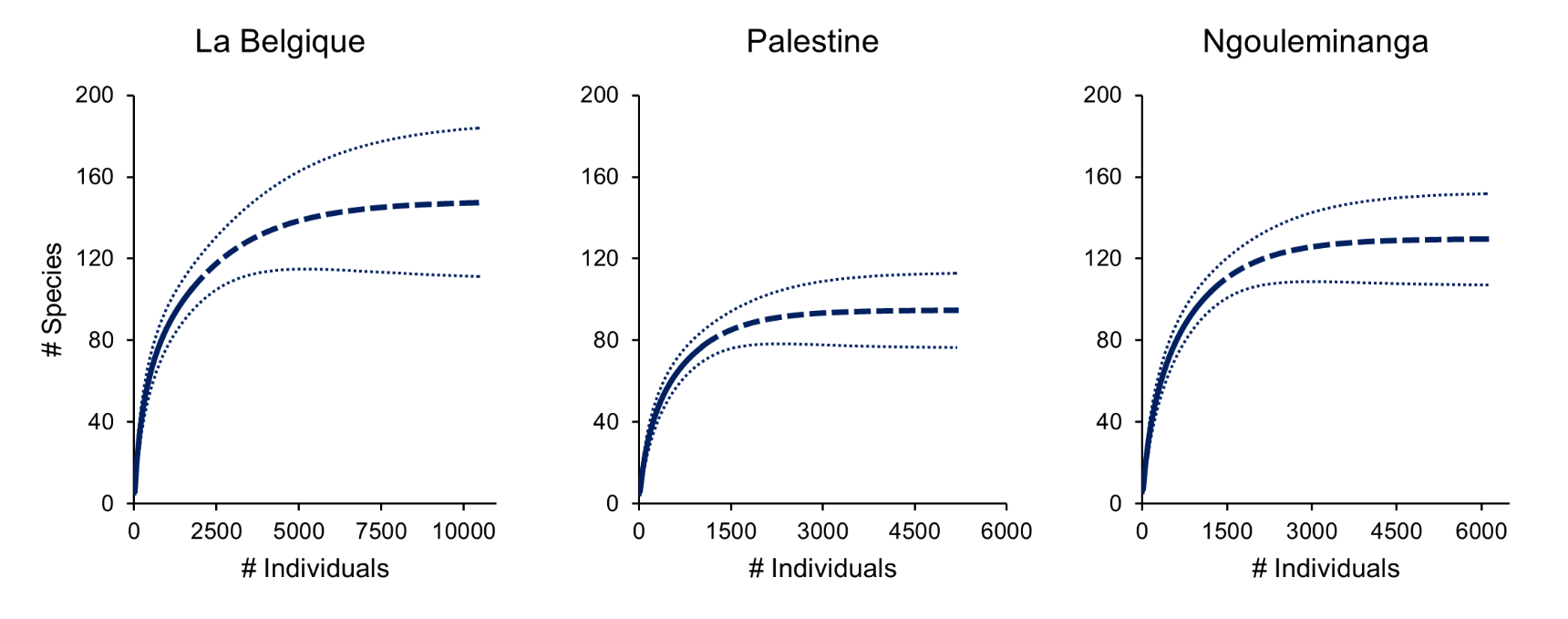


**Fig A: Extrapolated rarefaction curves for small woody stems (≤ 1m) at the three study sites. Curves were extrapolated until a plateau was reached.**
The curves for La Belgique (least defaunated) and Ngouleminanga (most defaunated) are very similar, and their 95% confidence intervals greatly overlap. Palestine (intermediate) has no more overlap with La Belgique after approximately 2860 stems. Palestine and Ngouleminanga move apart after approximately 530 stems, but the 95% confidence intervals start to overlap again after 2980 stems are reached. Differences are statistically non-significant when confidence intervals overlap.


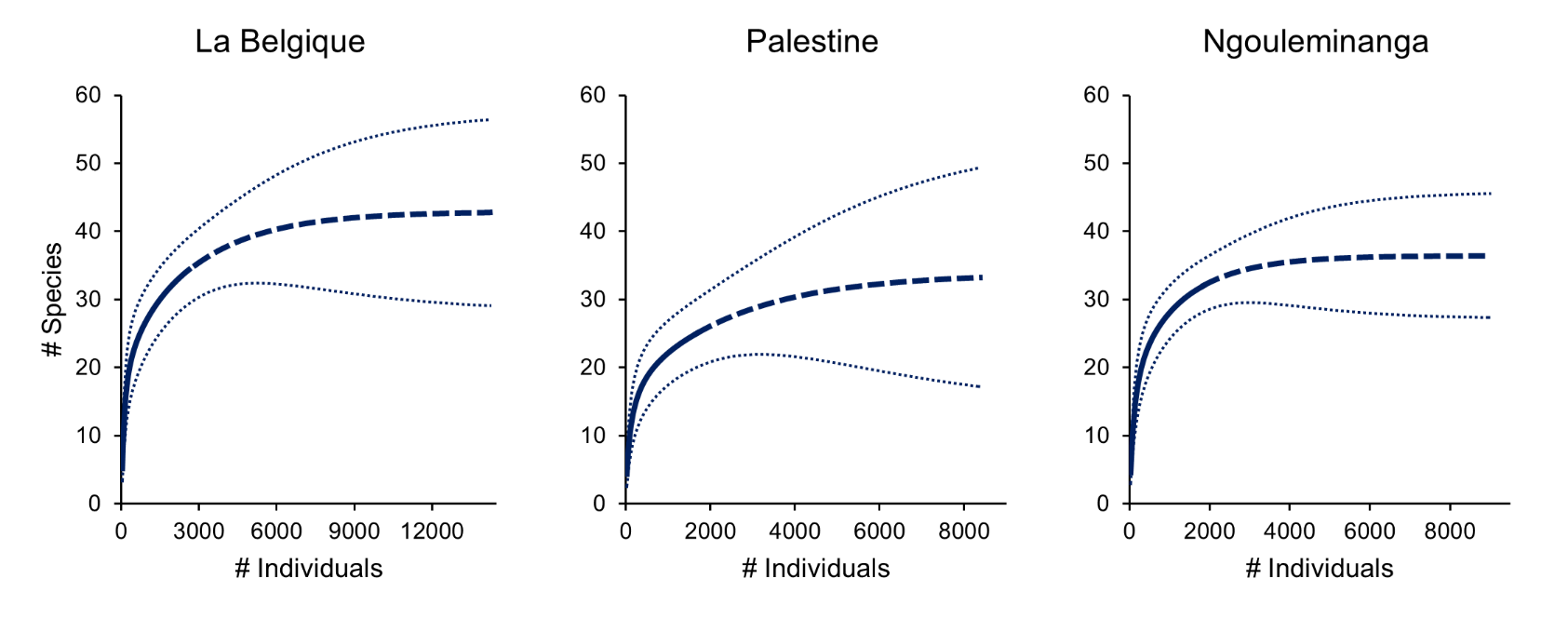


**Fig B: Extrapolated rarefaction curves for herbaceous stems at the three study sites. Curves were extrapolated until a plateau was reached.**
The curves for all three sites are very similar, and their 95% confidence intervals greatly overlap. Differences are statistically non-significant when confidence intervals overlap.


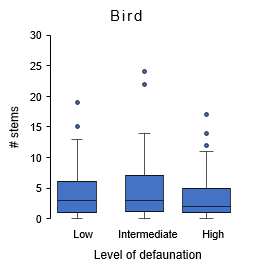

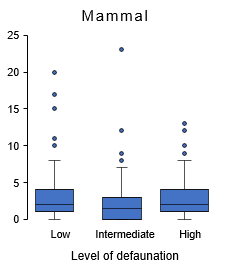

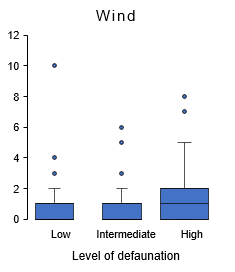

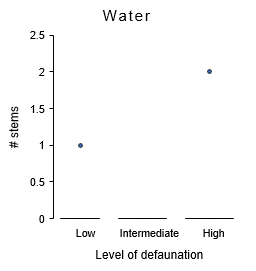

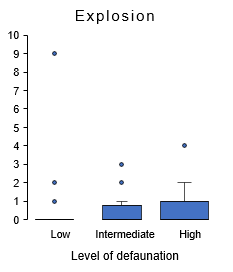

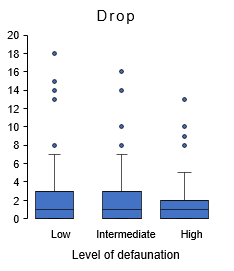


**Fig C: Boxplots of the abundance data for small woody stems (≤1m) with each dispersal mode at the three study sites.** Individual points represent the number of stems per plot (4m²). The three study sites: La Belgique (low), Palestine (intermediate), and Ngouleminanga (high). Significant differences between sites are indicated: * = P<0.05; ** = P < 0.01; *** = P < 0.001.


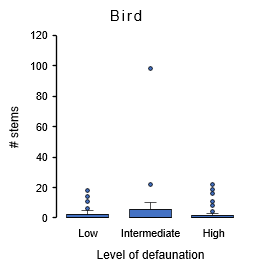

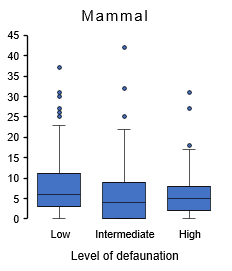

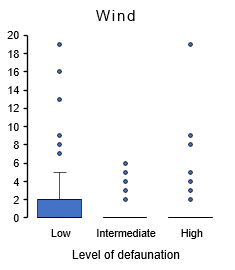

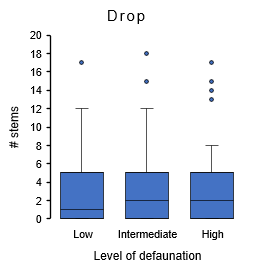


*

**Fig D: Boxplots of the abundance data for herbaceous stems with each dispersal mode at the three study sites.** Individual points represent the number of stems per plot (4m²). The three study sites: La Belgique (low), Palestine (intermediate), and Ngouleminanga (high). Significant differences between sites are indicated: * = P<0.05; ** = P < 0.01; *** = P < 0.001.
