# Supplementary material for: Defaunation is known to have pervasive, negative effects on tropical forests, but this is not the whole story
Source: PLoS One. 2023 Aug 31;18(8):e0290717. doi: 10.1371/journal.pone.0290717 (PMC10470957; doi:10.1371/journal.pone.0290717)
Supplement: S1 Table — (DOCX) [file pone.0290717.s006.docx]

# Supplementary information 2: Additional tables.

**Table A: Focal tree species description. The seed size, dispersal modes, seed predators, and references are shown.**

| **Scientific name** | **Seed size (mm)** | **Main dispersers** | **Other dispersers** | **Seed predators** | **References** |
| --- | --- | --- | --- | --- | --- |
| *Klainedoxa gabonensis* | 21–35 × 6–12 × 3–5 | E | G, C, M, U, A | R | [1–6] |
| *Tetrapleura tetraptera* | 9 × 7 × 4 | E, G | / | R | [3–5,7–9] |
| *Chrysophyllum lacourtianum* | 31 × 16 × 10 | E, G, C, M | / | U, R | [4,5,10] |
| *Duboscia macrocarpa* | 10 × 6 × 3 | E, G, C, M | A | U | [4–6,11–13] |
| *Antrocaryon klaineanum* | 20 × 18 × 11 | E, G, C, M | U | R | [4–6,12–17] |

Abbreviations: elephants (E), lowland gorillas (G), chimpanzees (C), monkeys (M), ungulates (U), rodents (R), and abiotic (A).

**Table B: Comparisons of the stem circumference of the sampled parent trees at the three study sites.**

| Species | Tree | Stem circumference (cm) | | | Test (df=2) | |
| --- | --- | --- | --- | --- | --- | --- |
|  |  | La Belgique | Palestine | Ngouleminanga | χ² | P-value |
| *K. gabonensis* | 1 | 288 | 296 | 240 | 1.32 | 0.52 |
|  | 2 | 323 | 180 | 220 |  |  |
|  | 3 | 359 | 411 | 180 |  |  |
|  | 4 | 270 | NA | 205 |  |  |
|  | 5 | 214 | NA | 300 |  |  |
|  | 6 | 230 | / | / |  |  |
|  | 7 | 225 | / | / |  |  |
|  | 8 | 206 | / | / |  |  |
| *T. tetraptera* | 1 | 132 | 159 | 175 | 0.072 | 0.96 |
|  | 2 | 116 | 129 | 104 |  |  |
|  | 3 | 177 | 650 | 145 |  |  |
|  | 4 | 130 | 170 | 215 |  |  |
|  | 5 | 198 | 122 | 253 |  |  |
|  | 6 | 130 | 116 | 112 |  |  |
|  | 7 | 159 | 146 | 116 |  |  |
|  | 8 | 205 | / | 200 |  |  |
| *C. lacourtianum* | 1 | 238 | NA | 255 | (U) 21 | 0.085 |
|  | 2 | 225 | / | 180 |  |  |
|  | 3 | 364 | / | 202 |  |  |
|  | 4 | 302 | / | / |  |  |
|  | 5 | 221 | / | / |  |  |
|  | 6 | 256 | / | / |  |  |
|  | 7 | 301 | / | / |  |  |
|  | 8 | 277 | / | / |  |  |
| *D. macrocarpa* | 1 | 847 | NA | 140 | (U) 49 | 0.083 |
|  | 2 | 835 | / | 210 |  |  |
|  | 3 | 310 | / | 200 |  |  |
|  | 4 | 265 | / | 300 |  |  |
|  | 5 | 359 | / | 160 |  |  |
|  | 6 | 173 | / | 305 |  |  |
|  | 7 | 143 | / | 260 |  |  |
|  | 8 | 304 | / | 155 |  |  |
| *A. klaineanum* | 1 | 435 | 246 | 205 | 2.52 | 0.28 |
|  | 2 | 271 | 125 | 352 |  |  |
|  | 3 | 287 | 294 | 254 |  |  |
|  | 4 | 259 | 215 | 272 |  |  |
|  | 5 | 313 | / | 200 |  |  |
|  | 6 | 234 | / | 353 |  |  |
|  | 7 | 412 | / | 246 |  |  |
|  | 8 | 243 | / | 350 |  |  |

**Table C: List of references used for the identification of dispersal modes.**

| Alexandre DY. Le rôle disséminateur des éléphants en forêt de Tai, Côte-d’Ivoire. Rev Ecol-Terre Vie. 1978;32:47-72. Available from http://hdl.handle.net/2042/58320. |
| --- |
| Astaras C, Waltert M. What does seed handling by the drill tell us about the ecological services of terrestrial cercopithecines in African forests?. Anim Conserv. 2010;13(6):568-578. doi: 10.1111/j.1469-1795.2010.00378.x |
| Barnes AD, Chapman HM. Dispersal traits determine passive restoration trajectory of a Nigerian montane forest. Acta Oecol (Montrouge). 2014;56:32-40. doi: 10.1016/j.actao.2014.02.002 |
| Blake S. The ecology of forest elephant distribution and its implications for conservation. Doctoral dissertation, University of Edinburgh; 2002. Available from: https://era.ed.ac.uk/handle/1842/15069 |
| Campos-Arceiz A, Blake S. Megagardeners of the forest–the role of elephants in seed dispersal. Acta Oecol (Montrouge). 2011;37(6):542-553. doi: 10.1016/j.actao.2011.01.014 |
| Carrière SM, André M, Letourmy P, Olivier I, McKey DB. Seed rain beneath remnant trees in a slash-and-burn agricultural system in southern Cameroon. J Trop Ecol. 2002;18(3):353-374. Available from: http://www.jstor.org/stable/3068629 |
| Chumacero de Schawe C, Durka W, Tscharntke T, Hensen I, Kessler M. Gene flow and genetic diversity in cultivated and wild cacao (Theobroma cacao) in Bolivia. Am J Bot. 2013;100(11):2271-2279. doi: 10.3732/ajb.1300025 |
| Clark CJ, Poulsen JR, Parker VT. The role of arboreal seed dispersal groups on the seed rain of a lowland tropical forest. Biotropica. 2001;33(4):606-620. doi: 10.1111/j.1744-7429.2001.tb00219.x |
| Clark CJ, Poulsen JR, Bolker BM, Connor EF, Parker VT. Comparative seed shadows of bird, monkey, and wind dispersed trees. Ecology. 2005;86(10):2684-2694. doi: 10.1890/04-1325 |
| Clark CJ, Poulsen JR, Connor EF, Parker VT. Fruiting trees as dispersal foci in a semi-deciduous tropical forest. Oecologia. 2004;139(1):66-75. doi: 10.1007/s00442-003-1483-1 |
| Daïnou K, Bauduin A, Bourland N, Gillet JF, Fétéké F, Doucet JL. Soil seed bank characteristics in Cameroonian rainforests and implications for post-logging forest recovery. Ecol Eng. 2011;37(10):1499-1506. doi: 10.1016/j.ecoleng.2011.05.004 |
| de Ruijter A. Barteria fistulosa Mast. In: PROTA4U [Internet]. 2007. Schmelzer GH, Gurib-Fakim A, editors. PROTA, Wageningen, Netherlands. Available from: https://www.prota4u.org/database/protav8.asp?h=M4&t=Barteria,fistulosa&p=Barteria+fistulosa#Synonyms |
| Dike MC, Aguguom AC. Fruits/seeds weights, flight patterns and dispersal distances of some Nigerian rainforest tree species. ARPN J Agric Biol Sci. 2010;5(3):56-64. Available from: https://citeseerx.ist.psu.edu |
| Epila J, Verbeeck H, Otim-Epila T, Okullo P, Kearsley E, Steppe K. The ecology of Maesopsis eminii Engl. in tropical Africa. Afr J Ecol. 2017;55(4):679-692. doi: 10.1111/aje.12408 |
| Evrard Q, Haurez B, Doucet JL. Le rôle des rongeurs dans la dispersion des diaspores en milieu forestier (synthèse bibliographique). Biotechnol Agron Soc Environ. 2017;21(1):66–79. doi: 10.25518/1780-4507.13448 |
| Ewango CEN. The liana assemblage of a Congolian rainforest: Diversity, structure and dynamics. Doctoral dissertation, Wageningen University. 2010. Available from: https://www.wur.nl/nl/show/The-liana-assemblage-of-a-Congolian-rainforest-Diversity-structure-and-dynamics.htm |
| Feer F. Morphology of fruits dispersed by African forest elephants. Afr J Ecol. 1995;33(3):279-284. doi: 10.1111/j.1365-2028.1995.tb00808.x |
| Feer F. Seed dispersal in African forest ruminants. J Trop Ecol. 1995;11(4):683-689. Available from: http://www.jstor.org/stable/2560089 |
| Fonge BA, Focho DA, Egbe EA, Tening AS, Fongod AN, Neba GA. The effects of climate and edaphic factors on plant colonisation of lava flows on Mount Cameroon. J Ecol Nat Environ, 2011;3(8):255-267. Available from: https://academicjournals.org |
| Forget PM, Jansen PA. Hunting increases dispersal limitation in the tree Carapa procera, a nontimber forest product. Conserv Biol. 2007;21(1):106-113. doi: 10.1111/j.1523-1739.2006.00590.x |
| Forget PM, Mercier F, Collinet F. Spatial patterns of two rodent-dispersed rain forest trees Carapa procera (Meliaceae) and Vouacapoua americana (Caesalpiniaceae) at Paracou, French Guiana. J Trop Ecol. 1999;15(3):301-313. Available from: http://www.jstor.org/stable/2560028 |
| Francis JK. Wildland shrubs of the United States and its territories: thamnic descriptions. Gen Tech Rep IITF-GTR-26. 2004;1:830. doi: 10.2737/IITF-GTR-26 |
| Gautier-Hion A, Duplantier JM, Quris R, Feer F, Sourd C, Decoux JP, et al. Fruit characters as a basis of fruit choice and seed dispersal in a tropical forest vertebrate community. Oecologia. 1985;65(3):324-337. doi: 10.1007/BF00378906 |
| Grubben GJH. Denton OA, editors. Plant Resources of Tropical Africa 2. Vegetables. Wageningen, Netherlands: PROTA Foundation; 2004. Available from: https://books.google.be/books?hl=nl&lr=&id=6jrlyOPfr24C |
| Hall JB, Swaine MD. Distribution and ecology of vascular plants in a tropical rain forest: forest vegetation in Ghana. Vol. 1. The Hague, Netherlands: Junk; 2013. doi: 10.1007/978-94-009-8650-3 |
| Hardy OJ, Delaide B, Hainaut H, Gillet JF, Gillet P, Kaymak E, et al. Seed and pollen dispersal distances in two African legume timber trees and their reproductive potential under selective logging. Mol Ecol. 2019;28(12):3119-3134. doi: 10.1111/mec.15138 |
| Haurez B, Tagg N, Petre CA, Brostaux Y, Boubady A, Doucet JL. Seed dispersal effectiveness of the western lowland gorilla (Gorilla gorilla gorilla) in Gabon. Afr J Ecol. 2018;56(2):185-193. doi: 10.1111/aje.12449 |
| Holbrook KM, Smith TB. Seed dispersal and movement patterns in two species of Ceratogymna hornbills in a West African tropical lowland forest. Oecologia. 2000;125(2):249-257. doi: 10.1007/s004420000445 |
| Hovestadt T, Yao P, Linsenmair KE. Seed dispersal mechanisms and the vegetation of forest islands in a West African forest-savanna mosaic (Comoé National Park, Ivory Coast). Plant Ecol. 1999;144(1):1-25. doi: 10.1023/A:1009764031116 |
| Jerome I, Chapman H, Iyiola T, Calistus A, Goldson S. Guild of Frugivores on three fruit-producing tree species (Polyscias fulva, Syzyguim Guineensis SUBSP. Bamensdae and Pouteria Altissima) in Ngel Nyaki Forest Reserve, a Montane Forest Ecosystem in Nigeria. j res for wildl environ. 2011;3(2):1-11. Available from: https://www.ajol.info/index.php/jrfwe/article/view/80303 |
| Jiofack T. Manniophyton fulvum Muell. Arg.. PROTA4U [Internet]. 2010. In: Brink M, Achigan-Dako EG, editors. PROTA, Wageningen, Netherlands. Available from: https://www.prota4u.org/database/protav8.asp?h=M4&t=Manniophyton,fulvum&p=Manniophyton+fulvum#Synonyms |
| Keay RWJ. Wind-dispersed species in a Nigerian forest. J Ecol. 1957;45(2):471-478. doi: 10.2307/2256930 |
| Koné I, Lambert JE, Refisch J, Bakayoko A. Primate seed dispersal and its potential role in maintaining useful tree species in the Taï region, Côte-d'Ivoire: implications for the conservation of forest fragments. Trop Conserv Sci. 2008;1(3):293-305. doi: 10.1177/194008290800100309 |
| Lemmens RHMJ, Louppe D, Oteng-Amoako AA, editors. Plant Resources of Tropical Africa 7 (2). Timbers 2. Wageningen, Netherlands: PROTA foundation; 2012. Available from: https://books.google.be/books?hl=nl&lr=&id=4MpmAgAAQBAJ |
| Lieberman D, Lieberman M, Martin C. Notes on seeds in elephant dung from Bia National Park, Ghana. Biotropica. 1987;19(4):365-369. doi: 10.2307/2388635 |
| Louppe D, Oteng-Amoako AA, Brink M, editors. Plant Resources of Tropical Africa 7 (1). Timbers 1. Wageningen, Netherlands: PROTA foundation; 2008. Available from: https://books.google.be/books?id=-nw-mZQ0kcEC |
| Mendes FDS. The impact of introduced animals and plants on São Tomé seed-dispersal network. Doctoral dissertation, University of Coimbra. 2017. Available from: http://hdl.handle.net/10316/82870 |
| Menke S, Böhning-Gaese K, Schleuning M. Plant–frugivore networks are less specialized and more robust at forest–farmland edges than in the interior of a tropical forest. Oikos. 2012;121(10):1553-1566. doi: 10.1111/j.1600-0706.2011.20210.x |
| Nchanji AC, Plumptre AJ. Seed germination and early seedling establishment of some elephant-dispersed species in Banyang-Mbo Wildlife Sanctuary, south-western Cameroon. J Trop Ecol. 2003;19(3):229-237. Available from: http://www.jstor.org/stable/4091960 |
| Nuñez CL, Clark JS, Clark CJ, Poulsen JR. Low-intensity logging and hunting have long-term effects on seed dispersal but not fecundity in Afrotropical forests. AoB Plants. 2019;11(1):ply074. doi: 10.1093/aobpla/ply074 |
| Petre CA. Effectivenness of western lowland gorilla (Gorilla gorilla gorilla) seed dispersal and plant-gorilla mutualism in southeast Cameroon. Doctoral dissertation, University of Liège. 2016. Available from: http://hdl.handle.net/2268/191040 doi: 10.1111/aje.12449 |
| Petre CA, Tagg N, Beudels-Jamar RC, Haurez B, Doucet JL. Western lowland gorilla seed dispersal: Are seeds adapted to long gut retention times?. Acta Oecol (Montrouge). 2015;67:59-65. doi: 10.1016/j.actao.2015.06.003 |
| Petre CA, Tagg N, Beudels-Jamar R, Haurez B, Salah M, Spetschinsky V, et al. Quantity and spatial distribution of seeds dispersed by a western lowland gorilla population in south-east Cameroon. J Trop Ecol. 2015;31(3):201-212. Available from: https://www.jstor.org/stable/26563541 |
| Poulsen JR. Logging and hunting alter patterns of seed dispersal and seedling recruitment in an afrotropical forest. Doctoral dissertation, University of Florida. 2009. Available from: https://ufdc.ufl.edu/UFE0024893/00001 |
| Poulsen JR, Clark CJ, Smith TB. Seed dispersal by a diurnal primate community in the Dja Reserve, Cameroon. J Trop Ecol. 2001;17(6):787-808. Available from: http://www.jstor.org/stable/3068616 |
| Puff C. The Significance of Gynoecium and Fruit and Seed Characters for the Classification of the Rubiaceae. Malayan Nat J. 2001;55(1&2):133-146. Available from: https://www.semanticscholar.org |
| Renner S. Plant dispersal across the tropical Atlantic by wind and sea currents. Int J Plant Sci. 2004;165(S4):S23-S33. doi: 10.1086/383334 |
| Renvoize SA, Clayton WD. Classification and evolution of the grasses. In G. P. Chapman (Ed.) Grass evolution and domestication. Cambridge, UK: Cambridge University Press; 1992. pp.15. Available from: https://books.google.be/books?id=dtY9AAAAIAAJ |
| Rosin C. Does hunting threaten timber regeneration in selectively logged tropical forests?. For Ecol Manag. 2014;331:153-164. doi: 10.1016/j.foreco.2014.08.001 |
| Spicer RA, Burnham RJ, Grant P, Glicken H. Pityrogramma calomelanos, the primary, post-eruption colonizer of Volcán Chichonal, Chiapas, Mexico. Am Fern J. 1985;75(1):1-5. doi: 10.2307/1546571 |
| Steentoft M. Flowering plants in west Africa. Cambridge, UK: Cambridge University Press; 1988. Available from: https://books.google.be/books?hl=nl&lr=&id=XqJrpbZOchIC |
| Takasaki H. Seed dispersal by chimpanzees: a preliminary note. Afr Stud Monogr. 1983;3:105-108. doi: 10.14989/67988 |
| Tédonzong LRD, Willie J, Keuko AMP, Kuenbou JK, Njotah G, Tchamba MN, et al. Using abundance and habitat variables to identify high conservation value areas for threatened mammals. Biodivers Conserv. 2018;27(5):1115-1137. doi: 10.1007/s10531-017-1483-9 |
| Theuerkauf J, Waitkuwait WE, Guiro Y, Ellenberg H, Porembski S. Diet of forest elephants and their role in seed dispersal in the Bossematie Forest Reserve, Ivory Coast. Mammalia. 2000;64(4):447-460. doi: 10.1515/mamm.2000.64.4.447 |
| Toirambe B. Tetrorchidium didymostemon (Baill.) Pax K.Hoffm.. In: PROTA4U [Internet]. 2008. Schmelzer GH, Gurib-Fakim A, editors. PROTA, Wageningen, Netherlands. Available from: https://www.prota4u.org/database/protav8.asp?h=M4&t=Tetrorchidium,didymostemon&p=Tetrorchidium+didymostemon#Synonyms |
| Torroba-Balmori P, Budde KB, Heer K, Gonzalez-Martinez SC, Olsson S, Scotti-Saintagne C, et al. Altitudinal gradients, biogeographic history and microhabitat adaptation affect fine-scale spatial genetic structure in African and Neotropical populations of an ancient tropical tree species. PloS one. 2017;12(8):e0182515. doi: 10.1371/journal.pone.0182515 |
| Trolliet F, Serckx A, Forget PM, Beudels-Jamar RC, Huynen MC, Hambuckers A. Ecosystem services provided by a large endangered primate in a forest-savanna mosaic landscape. Biol Conserv. 2016;203:55-66. doi: 10.1016/j.biocon.2016.08.025 |
| van der Maesen LJG, van der Burgt XM, van Medenbach de Rooy JM, editors. The Biodiversity of African Plants. Wageningen, Netherlands: Springer Science Business Media; 1996. doi: 10.1007/978-94-009-0285-5 |
| Voorhoeve AG. Liberian high forest trees: a systematic botanical study of the 75 most important or frequent high forest trees, with reference to numerous related species. Doctoral dissertation, Wageningen University. 1965. Available from: https://library.wur.nl/WebQuery/wurpubs/525678 |
| Vozzo JA, editor. Tropical Tree Seed Manual (No. 721). Washington, DC: US Department of Agriculture, Forest Service; 2002. Available from: https://books.google.be/books?hl=nl&lr=&id=RolEyk_23WIC |
| White LJ. Patterns of fruit-fall phenology in the Lopé Reserve, Gabon. J Trop Ecol. 1994;10(3):289-312. Available from: http://www.jstor.org/stable/2560315 |
| White L, Abernethy K. Guide de la végétation de la Réserve de la Lopé (2). Libreville, Gabon: ECOFAC; 1996. Available from: http://hdl.handle.net/1893/25985 |
| Whitney KD, Fogiel MK, Lamperti AM, Holbrook KM, Stauffer DJ, Hardesty BD, et al. Seed dispersal by Ceratogymna hornbills in the Dja Reserve, Cameroon. J Trop Ecol. 1998;14(3):351-371. Available from: http://www.jstor.org/stable/2559914 |
| Yumoto T, Maruhashi T, Yamagiwa J, Mwanza N. Seed-dispersal by elephants in a tropical rain forest in Kahuzi-Biega National Park, Zaire. Biotropica. 1995;27(4):526-530. doi: 10.2307/2388968 |
| Zona S, Henderson A. A review of animal-mediated seed dispersal of palms. Selbyana. 1989;11:6-21. Available from: http://www.jstor.org/stable/41759760 |

**Table D: Percentages of species with each dispersal mode identified at the three study sites.**

| **Small plots: woody stems ≤1m** | | | |
| --- | --- | --- | --- |
| **Dispersal mode** | **La Belgique** | **Palestine** | **Ngouleminanga** |
| Bird | 45.61% | 48.72% | 40.35% |
| Mammal | 64.91% | 61.54% | 70.18% |
| Drop | 29.82% | 28.21% | 29.82% |
| Explosion | 5.26% | 5.13% | 3.51% |
| Water | 5.26% | 5.13% | 3.51% |
| Wind | 21.05% | 20.51% | 19.30% |
| **Small plots: herbaceous stems** | | | |
| **Dispersal mode** | **La Belgique** | **Palestine** | **Ngouleminanga** |
| Bird | 7.14% | 10.00% | 8.33% |
| Mammal | 78.57% | 70.00% | 83.33% |
| Drop | 21.43% | 30.00% | 16.67% |
| Wind | 14.29% | 20.00% | 16.67% |
| **Large plots: woody stems >1m** | | | |
| **Dispersal mode** | **La Belgique** | **Palestine** | **Ngouleminanga** |
| Bird | 46.00% | 40.96% | 42.11% |
| Mammal | 74.00% | 79.52% | 75.44% |
| Drop | 27.00% | 27.71% | 28.07% |
| Explosion | 4.00% | 3.61% | 2.63% |
| Water | 3.00% | 2.41% | 3.51% |
| Wind | 17.00% | 15.66% | 18.42% |
| **Large plots: trees ≥10cm and lianas ≥5cm in diameter** | | | |
| **Dispersal mode** | **La Belgique** | **Palestine** | **Ngouleminanga** |
| Bird | 51.56% | 48.89% | 47.30% |
| Mammal | 71.88% | 83.44% | 77.03% |
| Drop | 26.56% | 33.33% | 31.08% |
| Explosion | 6.25% | 4.44% | 1.35% |
| Water | 0.00% | 2.22% | 4.05% |
| Wind | 17.19% | 13.33% | 18.92% |

Some species were assigned multiple dispersal modes; therefore, the sum of the percentages exceeds 100. The values are shown separately for the four analysed datasets.

**Table E: The number of species with identified dispersal modes at each site.**

| **Small plots: woody stems ≤1m** | | |
| --- | --- | --- |
| Site | Identified | Total |
| All together | 79 | 146 |
| La Belgique | 57 | 105 |
| Palestine | 39 | 77 |
| Ngouleminanga | 57 | 104 |
| **Small plots: herbaceous stems** | | |
| Site | Identified | Total |
| All together | 15 | 39 |
| La Belgique | 14 | 35 |
| Palestine | 10 | 25 |
| Ngouleminanga | 12 | 32 |
| **Large plots: all stems >1m** | | |
| Site | Identified | Total |
| All together | 130 | 216 |
| La Belgique | 100 | 168 |
| Palestine | 83 | 135 |
| Ngouleminanga | 114 | 184 |
| **Large plots: trees ≥10cm and lianas ≥5cm in diameter** | | |
| Site | Identified | Total |
| All together | 98 | 163 |
| La Belgique | 64 | 90 |
| Palestine | 45 | 58 |
| Ngouleminanga | 74 | 94 |

The total number of species recorded at each site is also shown. Separate values are shown for: woody stems ≤1m in small plots, herbaceous stems in small plots, all stems >1m in large plots, and trees ≥10cm and lianas ≥5cm in diameter in large plots.

**Table F: Density (stems/m²) comparisons for small woody (≤ 1m) and herbaceous stems between the three sites.**

|  | **La Belgique** | | **Palestine** | | **Ngouleminanga** | | **Test (df = 2)** | |
| --- | --- | --- | --- | --- | --- | --- | --- | --- |
| **Vegetation type** | **Median density** | **CI** | **Median density** | **CI** | **Median density** | **CI** | **χ²** | **P-value** |
| Small woody stems | 4 | 3.25; 4.62 | 3.25 | 2.75; 4.25 | 4.5 | 3; 5 | 1.84 | 0.40 |
| Herbaceous stems | 7.38 | 6.38; 8.25 | 6.25 | 5; 8 | 6.5 | 4.75; 7 | 3.40 | 0.18 |

The median density, 95% percentile confidence interval (CI), significance, and test statistic are shown.

**Table G: Comparison of the stem density (stems/m²) under focal trees at the three sites.**

| **Class 1** | **Median density** | | | **Test (df = 2)** | |
| --- | --- | --- | --- | --- | --- |
| **Species** | **La Belgique** | **Palestine** | **Ngouleminanga** | **χ²** | **P-value** |
| All together | 0.11 | 0.092 | 0.13 | 1.43 | 0.49 |
| *T. tetraptera* | 0.13 | 0.085 | 0.057 | 3.93 | 0.14 |
| *C. lacourtianum* | 0.043 | 0.27 | 0.13 | (U) 2.5 | 0.065 |
| *K. gabonensis* | 0.021 | 0.043 | 0.13 | 6.94 | 0.031 * |
| *D. macrocarpa* | 0.69 | 0.33 | 0.18 | (U) 52 | 0.040 * |
| *A. klaineanum* | 0.12 | 0.57 | 0.11 | 0.91 | 0.635 |
| **Class 2** | **Median density** | | | **Test (df = 2)** | |
| **Species** | **La Belgique** | **Palestine** | **Ngouleminanga** | **χ²** | **P-value** |
| All together | 0.021 | 0.021 | 0 | 2.02 | 0.36 |
| *T. tetraptera* | 0.014 | 0.028 | 0.028 | 0.615 | 0.735 |
| *C. lacourtianum* | 0.071 | 0.014 | 0.014 | (U) 22 | 0.051 |
| *K. gabonensis* | 0.028 | 0.014 | 0 | 9.66 | 0.0080 ** |
| *D. macrocarpa* | 0 | 0 | 0 | (U) 36 | 0.38 |
| *A. klaineanum* | 0.035 | 0.021 | 0.021 | 0.45 | 0.80 |

The top part of the table presents the results for Class 1 stems (<15cm, seedlings), while the bottom part presents the results for Class 2 stems (15–100cm, juveniles). The median abundance, significance, and test statistic (χ²) are shown. As the sample sizes for *C. lacourtianum* and *D. macrocarpa* were too small in Palestine (n = 1), the Wilcoxon-Mann-Whitney U test was used to compare the densities between La Belgique and Ngouleminanga. For these species the U statistic is presented instead of the χ². Significant differences between sites are indicated: * = P<0.05; ** = P < 0.01; *** = P < 0.001.

**Table H: The percentages of Class 1 and Class 2 stems ≤10m (Near) and 10–20m (Far) from the focal trees at each site.**

| **Class 1** | **La Belgique** | | **Palestine** | | **Ngouleminanga** | |
| --- | --- | --- | --- | --- | --- | --- |
| **Species** | **Near** | **Far** | **Near** | **Far** | **Near** | **Far** |
| All together | 57.72% | 42.28% | 45.95% | 54.05% | 29.95% | 70.05% |
| *T. tetraptera* | 48.72% | 51.28% | 53.06% | 46.94% | 18.60% | 81.40% |
| *C. lacourtianum* | 65.38% | 34.62% | 57.89% | 42.11% | 17.24% | 82.76% |
| *K. gabonensis* | 60.00% | 40.00% | 22.12% | 77.87% | 29.09% | 70.91% |
| *D.* *macrocarpa* | 59.27% | 40.73% | 52.17% | 47.83% | 31.88% | 68.12% |
| *A. klaineanum* | 48.68% | 51.32% | 52.76% | 47.24% | 35.29% | 64.71% |
| **Class 2** | **La Belgique** | | **Palestine** | | **Ngouleminanga** | |
| **Species** | **Near** | **Far** | **Near** | **Far** | **Near** | **Far** |
| All together | 44.09% | 55.91% | 30.56% | 69.44% | 24.04% | 75.96% |
| *T. tetraptera* | 44.44% | 55.56% | 30.77% | 69.23% | 10.71% | 89.29% |
| *C. lacourtianum* | 55.00% | 45.00% | 0.00% | 100.00% | 66.67% | 33.33% |
| *K. gabonensis* | 59.26% | 40.74% | 57.14% | 42.86% | NA | NA |
| *D. macrocarpa* | 100.00% | 0.00% | NA | NA | NA | NA |
| *A. klaineanum* | 26.00% | 74.00% | 20.00% | 80.00% | 27.40% | 72.60% |

The top part of the table presents the percentual distribution of Class 1 stems (<15cm, seedlings), while the bottom part presents the percentages for Class 2 stems (15–100cm, juveniles). These percentages are based on the proportion of the total number of stems found under focal trees. Data based on small sample sizes are marked in grey.

**References:**

1. Harris DJ. A Revision of the Irvingiaceae in Africa. Bull Jard Bot Natl Belg Bull Van Natl Plantentuin Van Belg. 1996;65: 143–196. doi:10.2307/3668184

2. Nchanji AC, Plumptre AJ. Seed germination and early seedling establishment of some elephant-dispersed species in Banyang-Mbo Wildlife Sanctuary, south-western Cameroon. J Trop Ecol. 2003;19: 229–237. doi:10.1017/S0266467403003262

3. Yumoto T, Maruhashi T, Yamagiwa J, Mwanza N. Seed-Dispersal by Elephants in a Tropical Rain Forest in Kahuzi-Biega National Park, Zaire. Biotropica. 1995;27: 526–530. doi:10.2307/2388968

4. Petre C-A. Effectivenness of western lowland gorilla (Gorilla gorilla gorilla) seed dispersal and plant-gorilla mutualism in southeast Cameroon. Doctoral dissertation, University of Liège. 2016. Available: http://hdl.handle.net/2268/191040 doi: 10.1111/aje.12449

5. Petre C-A, Tagg N, Beudels-Jamar R, Haurez B, Salah M, Spetschinsky V, et al. Quantity and spatial distribution of seeds dispersed by a western lowland gorilla population in south-east Cameroon. J Trop Ecol. 2015;31: 201–212. doi:10.1017/S0266467415000073

6. Clark CJ, Poulsen JR, Parker VT. The Role of Arboreal Seed Dispersal Groups on the Seed Rain of a Lowland Tropical Forest1. Biotropica. 2001;33: 606–620. doi:10.1111/j.1744-7429.2001.tb00219.x

7. Adesina S, Iwalewa EzekielO, Johnny I. Tetrapleura tetraptera Taub- Ethnopharmacology, Chemistry, Medicinal and Nutritional Values- A Review. Br J Pharm Res. 2016;12: 1–22. doi:10.9734/BJPR/2016/26554

8. Opabode JT, Akinyemiju OA, Ayeni OO. Plant regeneration via somatic embryogenesis from immature leaves in Tetrapleura tetraptera (Schum. & Thonn.) Taub. Arch Biol Sci. 2011;63: 1135–1145. doi:10.2298/ABS1104135O

9. Orwa C, Mutua A, Kindt R, Jamnadass R, Simons A. Tetrapleura tetraptera. Agroforestree Database: a tree reference and selection guide version 4.0.; 2009. Available: http://apps.worldagroforestry.org/treedb2/AFTPDFS/Tetrapleura_tetraptera.PDF

10. Lemmens RHMJ. Chrysophyllum lacourtianum de Wild. In: Louppe D, Oteng-Amoake AA, Brink M, editors. Plant resources of tropical Africa 7(1): timbers 1. Wageningen, Netherlands: PROTA foundation; 2008. pp. 161–163. Available: https://books.google.be/books?hl=nl&lr=&id=-nw-mZQ0kcEC

11. Brink M. Duboscia macrocarpa Bocq. In: Louppe D, Oteng-Amoake AA, Brink M, editors. Plant resources of tropical Africa 7(1): timbers 1. Wageningen, Netherlands: PROTA foundation; 2008. p. 226. Available: https://books.google.be/books?hl=nl&lr=&id=-nw-mZQ0kcEC

12. Poulsen JR, Clark CJ, Smith TB. Seed dispersal by a diurnal primate community in the Dja Reserve, Cameroon. J Trop Ecol. 2001;17: 787–808. doi:10.1017/S0266467401001602

13. Gautier-Hion A, Duplantier J-M, Quris R, Feer F, Sourd C, Decoux J-P, et al. Fruit characters as a basis of fruit choice and seed dispersal in a tropical forest vertebrate community. Oecologia. 1985;65: 324–337. doi:10.1007/BF00378906

14. Kémeuzé VA, Nkongmeneck BA. Antrocaryon klaineanum Pierre. In: PROTA4U [Internet]. 2011. Available: https://www.prota4u.org/database/protav8.asp?h=M4&t=Antrocaryon,klaineanum&p=Antrocaryon+klaineanum#Synonyms

15. Wang BC, Sork VL, Leong MT, Smith TB. Hunting of Mammals Reduces Seed Removal and Dispersal of the Afrotropical Tree Antrocaryon klaineanum (Anacardiaceae). Biotropica. 2007;39: 340–347. doi:10.1111/j.1744-7429.2007.00275.x

16. Feer F. Morphology of fruits dispersed by African forest elephants. Afr J Ecol. 1995;33: 279–284. doi:10.1111/j.1365-2028.1995.tb00808.x

17. Feer F. Seed dispersal in African forest ruminants. J Trop Ecol. 1995;11: 683-689. doi:10.1017/S0266467400009238
