# Supplementary material for: Defaunation is known to have pervasive, negative effects on tropical forests, but this is not the whole story
Source: PLoS One. 2023 Aug 31;18(8):e0290717. doi: 10.1371/journal.pone.0290717 (PMC10470957; doi:10.1371/journal.pone.0290717)
Supplement: Attachment — Submitted filename: Response to reviewers.docx [file pone.0290717.s009.docx]

Comments to the Author:

**Reviewer 1**

Overall:

This project was clearly an ambitious undertaking and data collection on the vegetation communities surrounding focal trees was extensive. Understanding changes in understory vegetation communities is an important but not well understood aspect of our comprehensive knowledge of ecological cascades triggered by defaunation in Afrotropical systems. As such, this data represents a valuable addition of knowledge and is certainly worth revising and publishing. This is an exceptionally challenging area to work in and we need more published data on defaunation’s consequences for Afrotropical systems. However, in its current state, this paper is not publishable. Improvements need to be made on organization, content, succinctness, and figure clarity.

Rep: Thank you for your feedback and suggestions. We have now edited the manuscript accordingly. Improvements to the organisation of the manuscript were made and the length of the manuscript was reduced.

Title:

The project reports on community composition changes and this would increase readership if included in the title (e.g. Community composition, demographic structure, and functional trait responses in forest understory vegetation along an Afrotropical defaunation gradient)

Rep: We decided to go with a title that has a link to the defaunation debate.

Introduction:

The introduction needs to be organized into clear paragraphs unified under topics. The content is good, but can and should be written more succinctly. There can be 1-2 paragraphs on vertebrate declines, a paragraph or 2 on potential effects on understory plant communities and background context, and in particular, the mechanisms by which the understory plant community might change should be explained, the Janzen Connell effect needs to be clearly explained and defined before it is discussed by name. Also hypotheses/research objectives should be clearly stated in a way that the reader can remember them as they move through the paper. As is, there is a lot of repetitive/superficial information that clouds the main points.

Rep: We have rewritten the introduction to make it more succinct and clearer. The hypotheses were rewritten to be more clear and easier to remember.

Line 47: clarify what WWF stands for before using an acronym.

Rep: This term was removed from the manuscript, and therefore does not require explanation.

Methods:

Ethics statement – I believe this goes in acknowledgements or elsewhere at the end of the paper unless the journal requires otherwise.

Rep: It is stated in the PLoS one guidelines that the ethics statement should be included in the methodology section. We have moved it to the end of the methodology section.

Study sites: throughout the paper, you need to clearly state which sites are faunally-intact, intermediate, or defaunated (or give them some other title that portrays their biological relevance in the context of your study).

Rep: we used the following terms: most defaunated site (Ngouleminanga), intermediate site (Palestine) and least defaunated site (La Belgique). By using these terms instead of the site names, the defaunation gradient is now clear thoughout the whole manuscript.

There is extra information in the methods that does not belong. Only include what is relevant to readers understanding how your methods were conducted and your study was designed. The information on the local uses of the focal tree species is interesting, but perhaps adds text not exactly relevant to this study. If it is relevant (if you want to argue that these species are ideal focal species because they are important human resources), then be more clear about why you include this information.

Rep: We edited the methods section and only retained the most relevant information.

‘Parent tree species’ could instead be referred to as ‘focal’ tree species.

Rep: We agree and used the term focal tree species.

The sub headings within the methods should be revised to better reflect that part of the study (e.g. 3.5.1 Recruitment under parent trees should be something specific such as “Conspecific density and demographics near parent trees”. Section 3.5.2 “General Forest Vegetation” could be “Understory and overstory composition”.

Rep: The headings were chaged to better reflect the content of the sections.

It is not clear to me what the point of the transects are for this specific study since you selected focal trees and used wedges and nearby plots (large and small) but I don’t see data being collected along the transects (I may have missed this though?).

Rep: The rectangular plots were placed along the transects at each kilometer mark. This is mentioned in the methodology L226-233 (section 3.3.2.).

The 3.6 “Data Collection” section should be deleted. Some parts can be moved elsewhere if they are needed to understand the study.

Rep: We integrated the relevant information from this section in other sections and removed the rest.

The paragraph in 3.6 on vegetation types seems unnecessary unless you are trying to state that the sites differed in important fundamental ways that affect your outcomes, in which case, you will need to account for that in you study, analysis, and interpretation. As is, the paragraph’s message is unclear.

Rep: This part was removed from the manuscript

3.7 Statistical analysis needs to be split up with what test you used for each element of your project listed under the appropriate subheading.

Rep: For most site comparisons the same analyses were used, and this is mentioned as such, to reduce repetition. Additional statistical analyses are listed per section.

The tables that add up to greater than 100% in describing the community composition of different dispersal modes are unfortunately not clear- it is hard to get information from this, especially with the whole adding up to >100. Please try to find another way to graphically portray this information, or, it might be appropriate to regroup some dispersal modes to be more inclusive of multiple disperser (this to me is more biologically intuitive- we know that most fruiting tree species are dispersed by a combination of animal groups rather than exclusively mammal or bird). Also, this information needs to be in the results not methods.

Rep: Even when grouping birds and mammals together there are many species that have multiple means of dispersal (including abiotic). As dispersal modes are not mutually exclusive, this will always result in percentages larger than 100%. Grouping some dispersal modes cannot change this reality, so it is better to keep the information in its current form. Furthermore, our defaunation gradient is based on mammal abundances. Looking at mammal dispersed species seperately allows for a better comparison along this gradient. The table has been moved to the Supplementary information (Table D in S2 Tables).

Please define what shrubs are as soon as you start discussing them.

Rep: This part was removed from the manuscript

Table 3 – again, this should be in results, but it is unclear how this fits with your hypothesis with the current categories (e.g. bamboo, cyper, ginger – where there specific hypotheses about what was expected with these groups in relation to defaunation pressure changing? Why are these groups singled out in your results? Is there a clearer way to summarize your plant community composition data that relates to the defaunation gradient?).

Rep: This part was removed from the manuscript

Results

Again, please include a reminder to the reader about the faunal/disturbance/defaunation status of each site as you give the findings for them. The results can be streamlined into succinct and complete stats and tables/figures.

Rep: We included several reminders of the level of defaunation at each site, and we made the results more succinct by removing unneccesary information and moving additional information to the supporting information.

Life form composition – it might be just because of an inconsistency in terminology, but this part is hard to understand in terms of how it relates to your hypotheses, and you are presenting so many results, it might be good just to eliminate or shorten this. Also, life forms usually mean vines versus shrubs versus herbs versus trees, or something along those lines, while you seem to be using that term in a unique way that I haven’t seen before, and I suspect that might confuse many readers.

Rep: Thank you for bringing this up. We have removed this section from the manuscript.

Some results can be presented as a table or as a figure, and you don’t need both in the main text (e.g. put figure in main text, put table in supplementary materials).

Rep: We have moved several figures and tables to the supplementay information (S2 Tables and S4 Figures).

Indicator species – I think this analysis should be removed. You only have 3 sites, and I don’t think that the effects of defaunation in the understory that you are interested in would be reflected by an indicator species. If you do decide to keep this, please explain it in your hypotheses and justify pursuing that analysis. Given that you would want all these sites to be similar except with differences in disturbance levels, high plant species overlap and a lack of indicator species differences is what you would expect and want to find.

Rep: We agree and have removed this analysis from the manuscript

Discussion:

Organize paragraphs around 3-4 main take away messages, remove bolded statements, shorten and make clearer and more succinct.

Rep: The discussion has been rewritten to be more succinct and clearer

Figures:

Map: includes larger image of Africa with Cameroon highlights and the region within Cameroon you are working also highlighted. If possible, plot the GPS locations of the focal trees. All boxplots: # seedlings: change to number of seedlings per unit area (you had a specific area in which you sampled, include that). Include on the x axis the disturbance level of each site. Include a figure title over each figure because the axes are the same and it can be confusing at a glance. Put distances for legend rather than near and far (e.g. “> x m from parent tree”)

Rep: A new clearer map of the study area was created based on your suggestions (Fig 1). The exact GPS coordinates of the focal trees are not available. The x axis of the boxplots have been updated and titles have been added as suggested. For the wedges we used the same methodology as Cordeiro & Howe and kept the # of stems on the y-axis to make comparison with this study easier. Additionally, the sampling area >10m and <10m away differs, adding this in the Y-axis would make the figure more confusing. For all other boxplots the sample area was added in the figure caption.

Species rarefaction curves could probably go into supplementary materials. Figure 5 b gives no real information and figures like this shouldn’t be included- rather find another way (probably just in text) to give this data.

Rep: Thanks for the suggestion. We moved the rarefaction curves to the supplemantary information (Fig A & B in S4 Figures). Figure 5b was removed and this data is mentioned in text in S5 Supplementary material.

**Reviewer 2**

All along the manuscript

- Supplementary files being called in random order, starting from S4 L240, for instance.
Rep: Thank you for your feedback and suggestions. Supplemantary information is now called in order.

Introduction

I think that the introduction contains the information that it needs and is generally well-written. But it is highly unusual to see a research article with an introduction of 16 paragraphs for 6 pages... And even more to see 80 citations in the introduction… (!) In fact, the over-abundance of reference makes it extremely difficult for the reader to navigate through the authors references and to check them. Most of this is explained by incoherent citing, when the authors would repeatedly cite new small case studies, rather than keeping referring to some of the very good and broad literature reviews that were published by Poulsen, Dirzo, and others… I invite them to select a couple of them, and to use them to support general and well-documented statements about defaunation and the cascade consequences on plant communities. These reviews summarized the state of the art in a much broader and convincing way than it could be done in a single Introduction, and this is not the aim of an Introduction to cover an entire research topic anyway. I propose them to cut the number of citations in the introduction by more than half, to make it again accessible to the reader.

Rep: We have shortened and rewriten the introduction and reduced the number of citations.

A lot of introduced material is a bit redundant across paragraphs and could be condensed in 4-6 paragraphs I think that the topic is well-introduced by the author, but I also feel that the introduction could be greatly shorten without losing information. It has been a bit tedious to read through.

Rep: We have shortened and rewriten the introduction.

L47-51. I recommend you to cite (Dirzo, et al. 2014. Science) here, and to add (Ceballos, et al. 2017, PNAS) that already cited, rather than push forward an NGO report.
Paragraph 1 and 2. Consider fusing them together.

Rep: We have shortened and rewriten the introduction.

L88. Consider changing your sentence to: “The Janzen-Connell model predicts a decrease of recruitment among seeds closer to the parent-tree due to density and distance-dependent mortality”

Rep: We have shortened and rewriten the introduction.

L112-L115. Lifeform abundance: please clarify what you mean here, this term is ambiguous to me. You precise later that you are in fact talking about tree or liana dominance. I recommend you to use this term. “Shift toward trees/lianas” does not make much sense neither.

Rep: This analysis has been removed from the manuscript.

L121. Consider citing a review on the topic to support that claim, not a case study.

Rep: This sentence was removed from the manuscript.

Material and Method

This part is more chaotic than the introduction. A lot of unnecessary details are provided, disorganized, while important information is omitted. Please, really consider my comments hereunder.

Rep: Thank you for your feedback. We removed the unnecessary details and provided all relevant information.

There is maybe something I would like the authors to clarify at least during the review process and/or within the manuscript. What is the nature of this “Unpublished Master thesis”? I assume it is the work of an alumni student. In this case, since you are presenting his or her data anyway in the Supplementary Material S4, why to even mention it? Why not to merge the manuscript and the thesis into 1 (at least to precise that larger mammals’ occurrence and abundance were documented by this or that methodology, for this amount of time…, in the material and methods)? Why not providing a link where to actually consult this thesis? I feel that this should be somehow clarified, as the authors just currently ask us to trust them that the defaunation gradient has been well-documented. I think the part 3 of the Material and Methods should then be rewritten to convince better the reader of the existence of this defaunation gradient, rather than making it mostly descriptive.

Rep: We have included the methodology and results from this thesis in the supplementary information. In addition, the author and supervisors of that thesis are now included as co-authors of this manuscript.

L196-197. “The reserve has a high…”: please remove, it is both obvious and meaningless if compared to nothing.

Rep: This sentence was remved from the manuscript.

L210, L219… I do not see the point of citing Material that the reader cannot access. In both case you are asking the reader to trust you. Because a large part of your study relies on the fact that the anthropogenic pressures are documented by you (or at least an alumni or someone that worked at the same study site), please consider providing a way for the reader to either 1/ access the Master thesis (sometime Universities are providing them online – mine was) or 2/ fuse them in a single manuscript.

Rep: We have included the methodology and results from this thesis in the supplementary information (see S1 Supplementary Information on mammal abundance).

L238-257. I strongly recommend you to either 1/ greatly summarize in couple of sentences, or 2/ Summarize in a Figure. This paragraph is only the description of the supplementary material.

Rep: We have included the methodology and results from this thesis in the manuscript and more detailed in the supplementary information S1 Supplementary Information.

L259-307. Similar comment as above. Why to go so much into details within the text, plants by plants, why you could have made a Figure, with 5 pictures, with the seeds and a scale, and a Table with the dispersers, the references, and everything else? This would be much more comfortable for the reader to navigate.

Rep: We created a clear table (Table A in S2 Tables) with the dispersers of the five focal tree species.

Part 3.5.1. and Part 3.5.2. Consider creating a figure to illustrate your design. It took me several readings to picture it. This figure could be merge with your Fig. 1, under 3 subcharts A,B,C for instance (A: the map; B: recruitment design; C: vegetation survey).

Rep: Fig 2 has been added showing the two vegetation sampling designs.

L345-351. Consider summarizing all of this in 1 Table.

Rep: This paragraph was summarized in L243-244

L352-358. The generally accepted term is that you identified your plants into morphospecies based on morphological criteria. Please use this term instead of “sorted by lifeforms”.

Rep: We agree and used the term morphospecies.

I have many concerns here that needs clarifications: no vouchers were collected nor photographed, and no herbarium was built I therefore presume? If yes, please details it (e.g., where it is stored, who identified it, with which books etc…), if no, this is a bit of concern… Some Diospyros spp., Homalium spp., Drypetes spp., and other Beilschmiedia spp. for instance can be hard to properly identified in the field, even by our best botanists.

Rep: In 2001, Projet grands singes of the Centre for Research and Conservation of the Royal Zoological Society of Antwerp created a field research station in a tropical forest of Cameroon where the present research was conducted. Over the years, many research projects involving botanical data collection have been successfully conducted and have resulted in many scientific publications (e.g., Willie et al., 2013; Willie et al., 2014; Tédonzong et al., 2018). During these projects, plant vouchers were collected and submitted for identification to the National Herbarium of Cameroon. As a result, a checklist of plants occurring in the area (local names with corresponding scientific names) was created to facilitate botanical research in the area. Therefore, there was no further need to collect or photograph vouchers during the present study unless a new plant species was discovered. We should also mention that whenever a local name on the checklist correspond to more than one scientific name, descriptive French words are added to the local name to create unique local names corresponding to unique scientific names. Hence, these unique local names make it possible to differentiate between different species. These unique local names are known by the local botanist who used them when identifying plants included in the present study. This local botanist has been involved in previous botanical research projects that resulted in the existing checklist of plants.

How did you remember the morphospecies of liana after the 20th morphospecies and more (or lifeform, as you say)? Did you take pictures of them? Did you standardize them across the sites to make it comparable? Did you build-up your own field-guide?

Rep: As mentioned earlier, we used an existing checklist of plants with both local and scientific names. Therefore, we did not build a new field guide. Based on scientific names, it was possible to search the literature and obtain further information regarding the biology or ecology of each species.

Was it the same parataxonomist that identified them? Why is he/she not a co-author if he/she is that much of an authority (a practice more and more accepted against “parachute science”: Stefanoudis et al., 2021, Current Science)?

Rep: Yes, the same local botanist identified all stems. He has been involved in many projects before and shows excellent mastery of plants occurring in the study area. The contribution of the local botanist during plant identification in the field is acknowledged in the acknowledgment section of the paper where his name features. We believe acknowledging his contribution this way is fair, and we do not think he can be added as co-author.

Which proportion are identified to genus/species-level, which one to morphospecies? Many of these local terms also refer to genus-level: how did you choose the scientific name back from the field (i.e., “Asas” in this region of Cameroon, for instance, is a generic term which refers to many Macaranga spp.)?

Rep: These numbers were added in L241-243 and please refer to our answers above.

Please, detail a bit more your methodology: I am ready to accept it, as I understand the reality of the field, but you need to convince me that the number of species that you provide make any sense. So far, I am not convinced, as I am not even sure what was truly done in the field.

Rep: Some details from the answers provided in this rebuttal letter have been added to the methodology to provide further clarification. See L235-243

L369. You probably mean “taller”, not larger.

Rep: We used the word taller L232

L378. Which data? Be precise please.

Rep: We clarified what data is non-normal L248-249

L401. I was waiting for that number for long time.

Rep: We moved this paragraph to L218-221.

Tables and Figures

Fig. 1. The map is very poorly done and barely readable. Please, I strongly recommend you to provide a better one, by approaching a colleague with good GIS skills for instance. Here are some flaws in my opinion:
- La Belgique barely visible.
- Crosses representing Latitude/Longitude are useless. The numbers are also provided along both axes, but the used coordinate system isn’t mentioned anywhere.
- Intriguing “0”s are floating in the longitude/Latitude axes. The decimals are not visible, and I suspect that such large number of decimals lead to a precision <1 m, which is virtually impossible in the field.
- A smaller map of the Dja Reserve embedded in the Figure would help a reader familiar with the study site (your audience) to immediately get where you work.
- I think that 2 layers for rivers were used: the Dja one and the river ones. In this case, I think that they don’t really overlap well or that their grains are different: I am confused how a “small river” would serpentine around the Dja River like that. I just think that the Dja river is represented twice in your map. Or am I wrong?
- All writings and symbols are way too small, the lines too. Please, make it readable.
- Is the “N” of North draw with a pen? Not a flaw, just odd.

Rep: A clearer map based on your remarks was created (Fig 1).

References

Willie J, Petre CA, Tagg N, Lens L. Density of herbaceous plants and distribution of western gorillas in different habitat types in south‐east Cameroon. African Journal of Ecology. 2013;51(1):111-121. doi: 10.1111/aje.12014

Willie J, Tagg N, Petre CA, Pereboom Z, Lens L. Plant selection for nest building by western lowland gorillas in Cameroon. Primates. 2014;55:41-49. doi: 10.1007/s10329-013-0363-5

Tédonzong LRD, Willie J, Keuko AMP, Kuenbou JK, Njotah G, Tchamba MN, ..., Lens L. Using abundance and habitat variables to identify high conservation value areas for threatened mammals. Biodiversity and conservation. 2018;27:1115-1137. doi: 10.1007/s10531-017-1483-9
